# *N*-monoarylacetothioureas as potent urease inhibitors: synthesis, SAR, and biological evaluation

**DOI:** 10.1080/14756366.2019.1706503

**Published:** 2019-12-27

**Authors:** Wei-Yi Li, Wei-Wei Ni, Ya-Xi Ye, Hai-Lian Fang, Xing-Ming Pan, Jie-Ling He, Tian-Li Zhou, Juan Yi, Shan-Shan Liu, Mi Zhou, Zhu-Ping Xiao, Hai-Liang Zhu

**Affiliations:** a National Demonstration Center for Experimental Chemistry Education, Hunan Engineering Laboratory for Analyse and Drugs Development of Ethnomedicine in Wuling Mountains, Jishou University, Jishou, PR China; b State Key Laboratory of Pharmaceutical Biotechnology, Nanjing University, Nanjing, PR China

**Keywords:** Urease inhibitor, *N*-monoarylacetothiourea, *Helicobacter pylori*, SPR, molecular dynamics

## Abstract

A urease inhibitor with good *in vivo* profile is considered as an alternative agent for treating infections caused by urease-producing bacteria such as *Helicobacter pylori*. Here, we report a series of *N*-monosubstituted thioureas, which act as effective urease inhibitors with very low cytotoxicity. One compound (**b19**) was evaluated in detail and shows promising features for further development as an agent to treat *H. pylori* caused diseases. Excellent values for the inhibition of **b19** against both extracted urease and urease in intact cell were observed, which shows IC_50_ values of 0.16 ± 0.05 and 3.86 ± 0.10 µM, being 170- and 44-fold more potent than the clinically used drug AHA, respectively. Docking simulations suggested that the monosubstituted thiourea moiety penetrates urea binding site. In addition, **b19** is a rapid and reversible urease inhibitor, and displays nM affinity to urease with very slow dissociation (*k*_off_=1.60 × 10^−3^ s^−1^) from the catalytic domain.

## Introduction

*Helicobacter pylori*, a gram-negative and microaerophilic bacterium, colonises the gastric mucosa of over 50% of the global population^1^. In some individuals, chronic infection can induce a significant inflammatory response, which triggers a loss of gastric epithelial cells, resulting in gastric, duodenal ulcers and approximately 90% of cases of intestinal-type gastric carcinoma^2^. With the help of urease (EC 3.5.1.5), a nickel dependent metalloenzyme with an ability to catalyse the hydrolysis of urea to ammonia and carbamates, *H. pylori* creates a local neutral environment for survival by continuously releasing ammonia into succus gastricus^3^. In addition, Eaton et al. and Karita et al. demonstrated that urease-negative mutant of the *H. pylori* strain was unable to colonise the gastric mucosa under the acidic conditions of the stomach^4^^,^^5^. Therefore, urease is considered as a virulence factor playing an essential role for establishment of *H. pylori* colonisation in human. Therefore, urease inhibitors could serve as drugs for treating *H. pylori* caused disease such as gastritis and peptic ulcers^6^.

In the past decades, thousands of urease inhibitors have been reported, and they were designed exclusively by either attacking the metallocenter or mimicking the substrate of ureases^7–10^. However, urease has a highly specific substrate urea, which makes it very challenging for the development of urease inhibitors. In spite of enormous efforts being made, only acetohydroxamic acid (AHA) has so far been approved by FDA for the treatment of urinary tract infections. Novel urease inhibitors with high potency are urgently needed. We therefore have focussed our efforts on this field for several years, and some potent urease inhibitors with structural diversity were reported such as catechols, diarylethylenes, flavonoids, arylamides, and hydroxamic acids^11–15^.

Thiourea derivatives, substrate analogues of urease, were reported as potential urease inhibitors^16–18^. In chemical structure, they are all *N*,*N*′-disubstituted thioureas, resulting in a different binding mode from that of urea^18^^,^^19^, which may be caused by the high hindrance around thiourea score. Considering that the urea binding pocket is narrow and small, we believe that the thiourea moiety of *N*-monosubstituted thioureas could reach the bottom of the urea-binding pocket and chelate the two Ni atoms due to a tinny head. Based on this hypothesis and as a continuation of urease inhibitor screening, a series of *N*-arylacetothioureas were designed and synthesised. Some resulted compounds showed excellent potency against *H. pylori* urease.

## Materials and methods

### Biology materials

Protease inhibitors (Complete, Mini, EDTA-free) were purchased from Roche Diagnostics GmbH (Mannheim, Germany) and Brucella broth was from Becton Dickinson and Company (Sparks, MD). Sheep sterile and defibrinated blood were from Hyclone (Logan, UT).

### Bacteria

*H. pylori* (ATCC 43504; American Type Culture Collection, Manassas, VA) was grown in Brucella broth supplemented with 10% sheep sterile and defibrinated blood for 24 h at 37 °C under microaerobic conditions (5% O_2_, 10% CO_2_, and 85% N_2_), as our previously described literature^13^^,^^14^.

### Preparation of *H. pylori* urease

For urease inhibition assays, 50 mL broth cultures (2.0 × 10^8^ CFU/mL) were centrifuged (5000×*g*, 4 °C) to collect the bacteria, and after washing twice with phosphate-buffered saline (pH 7.4), the *H. pylori* precipitation was stored at –80 °C for 8 h, and then was returned to room temperature, and after addition of 3 mL of distilled water and protease inhibitors, sonication was performed for 60 s. Following centrifugation (15,000×*g*, 4 °C), the supernatant was desalted through Sephadex G-25 column (PD-10 columns, Amersham Pharmacia Biotech, Uppsala, Sweden). The resultant crude urease solution was added to an equal volume of glycerol and stored at 4 °C until use in the experiment.

### Measurement of urease inhibitory activity

Urease activity was determined in the 96-well assay plate by measuring ammonia production using the indophenol method as described by Weatherburn^20^. Briefly, to each well, 50 µL of urea (10 mM) in phosphate buffer solution was added in a mixture of 25 µL (10 U) of *H. pylori* urease and 25 µL of the test compound, which was incubated at 37 °C for 0.5 h. Fifty µL of phenol reagent (containing 127 mM phenol and 0.168 mM sodium nitroprusside) and 50 µL of alkali reagent (containing 125 mM NaOH and 0.168 mM NaOCl) were added in turn. The resulted mixture was incubated at 37 °C for another 0.5 h for colouration developing. The increasing absorbance was measured at 630 nm after 50 min using a microplate reader (Molecular Devices, San Jose, CA). Percentage inhibitions were calculated from the following formula (Equation (1)). Experiments were performed in triplicate and AHA was used as reference drug, and the IC_50_ value was determined as the concentration of compound that give 50% inhibition of maximal activity. As for the urease assay of intact cells, 25 µL (10 U) of *H. pylori* urease was replaced by 25 µL of cell suspension (4.0 × 10^7^ CFU/mL).

### Determination of minimal inhibitory concentrations

The minimum inhibitory concentration (MIC) values were determined using the broth microdilution protocol according to the methods of the Clinical and Laboratory Standards Institute (CLSI)^21^.

### Ligand affinity study

The binding kinetics of selected compounds were assayed via surface plasmon resonance (SPR) using an OPEN SPR instrument (Nicoya Lifesciences, Kitchener, Canada). First, urease dissolved (50 µg/mL) in PBS buffer (1 mM KH_2_PO_4_, 155 mM NaCl, 3 mM Na_2_HPO_4_-12H_2_O, pH 7.4), was immobilised to a CM5 chip using a standard amine coupling procedure^22^. Then, SPR measurements were carried out in PBS, and stock solutions were diluted in the same buffer. Data were collected with OpenSPR control software. Experiments were performed by monitoring the refractive index changes as a function of time under constant flow rate of 20 µL/min. The relative amount of inhibitor bound to the urease was determined by measuring the net increase in refractive index over time compared to control running buffer. There is an inline subtraction of reference surface during the run. This change is usually reported in response units (RU). Sensograms were processed and analysed using TraceDrawer software. The binding curves were fit to determine the equilibrium dissociation constant (*K*_D_).

### Enzyme kinetic study

Based on the indophenol method, the velocity of ammonia production (*V*) was measured in the presence of concentration gradients of urea ([*S*]) for every specific concentration of selected compounds ([*I*]). Nonlinear fitting curves to data of *V* and [*S*] were used to determine the type of enzyme inhibition based on the general kinetics equation (Equation (1)). Subsequently, the resulted fitting constants ($\frac{K_{m}}{V_{\max}}(1+\frac{\left[I\right]}{K_{i}})$ or $\frac{1}{V_{\max}}(1+\frac{\left[I\right]}{K_{i}^{\prime }})$) and [*I*] were linearly fitted to give kinetic parameters $K_{i}$ and $K_{i}^{'},$ respectively. The experimental assay was performed against a pure urease (Jack bean urease) for the consideration of precision.

(1) $$V=\frac{V_{\max}[S]}{K_{m}\left(1+\frac{\left[I\right]}{K_{i}}\right)+\left(1+\frac{\left[I\right]}{{K^{\prime }}_{i}}\right)[S]}$$

### Protocol of docking study

Molecular docking of compounds **b19** into the structure of *H. pylori* urease complex structure was carried out using SYBYL-X version 2.1.1 software suite (Tripos, Inc., St. Louis, MO)^23^. The X-ray structure of urease from *H. pylori* was downloaded from the Protein Data Bank (PDB code: 1e9y)^24^ and was modified by adding hydrogen atoms and removing water as well as cocrystallised substrate (AHA). The active site was defined as all the amino acid residues confined within a 5 Å radius sphere centred about AHA, and the composite structure without original ligand was utilised as the *in silico* model for docking studies. Default parameters and values within the minimisation dialogue were used except where otherwise mentioned. The docked conformations of ligands were evaluated and ranked using Surflex*-*dock and four scoring functions implemented in the CSCORE software module within the SYBYL-X environment. The CSCORE module allowed consensus scoring that integrated multiple well-known scoring functions such as ChemScore, D-Score, G-Score, and PMF-Score to evaluate docked ligand conformations.

### Molecular dynamic simulations protocol

Molecular dynamics was performed using Desmond 4.2 with the standard RESPA integration and 2 fs time step. The TIP3P water model was used and exhibited well when combining with the OPLS force field. Each simulation system including the prepared protein, the inhibitor, and several Cl^–^ added to achieve charge neutrality was immersed in a cubic box (10 Å). First, all the prepared systems were minimised until a gradient threshold (25 kcal/mol/Å) was reached by the steepest-descent (SD) method, and then coupled to the Berenson thermostat with 300 K reference temperature and the Berendsen barostat with 1.01325 bar reference pressure. The calculation of long-range electrostatics was based on the Particle-Mesh Ewald method. The cut-off for coulomb interaction was set at 9.0 Å. After equilibrated, all the systems began to run in the NPT ensemble for 6 ns. The relative RMSD and root mean square fluctuation (RMSF) were calculated based on the analysis of the MD trajectories by the VMD (version 1.9.1) and xmgrace tool.

### Cytotoxicity assay

The stock solutions of the selected compounds (250 µg/mL, in PBS) were prepared in medium. L-02 cells and P69 cells were grown in medium (90% RPMI-1640 medium, 10% foetal bovine serum, 1% penicillin/streptomycin) and maintained at 37 °C in a humidified atmosphere containing 5% CO_2_, respectively. Cells were seeded in 96-well cell culture plates. On the day when seeding, the cells were exposed to 250 µg/mL of compounds and further cultured for 72 h at 37 °C. Cell proliferation was then determined using the thiazolyl blue tetrazolium bromide (MTT) assay.

### Chemistry

All chemicals (reagent grade) used were purchased from Sinopharm Chemical Reagent Co., Ltd (Shanghai, China). Melting points (uncorrected) were determined on a XT4 MP apparatus (Taike Corp., Beijing, China). EI mass spectra were obtained on Agilent 6120 mass spectrometer, and ^1^H NMR spectra were recorded on a Bruker AV-600 spectrometer at 25 °C with TMS and solvent signals allotted as internal standards. Chemical shifts were reported in ppm (*δ*). Elemental analyses were performed on a Foss Heraeus CH*N-*O-Rapid instrument and were within ±0.4% of the theoretical values.

### General procedure for the preparation of compounds b

A selected arylacetic acid (10 mmol) was solved in SOCl_2_ (50 mL), which was heated to 90 °C and stirred for 1–2 h until the reaction was completed. The crude product (**a1**–**a29**) was furnished after removal of SOCl_2_ under reduced pressure. To the resulting residue, 50 mL of toluene was added and stirred for 30 min at room temperature. Thiourea (40 mmol) was subsequently added, and the reaction solution was heated to 100 °C for 1.5–2 h. After the end of the reaction was established by TLC, the solvent was removed under vacuum, and excess saturated NaHCO_3_ solution was added. The resulted mixture was extracted with ethyl acetate, dried over MgSO_4_, filtered, and concentrated under vacuum. The product was purified by a silica gel column using ethyl acetate and petroleum ether as eluent to afford compound **b** (**b1**–**b29**) in moderate to high yield as white powder (all the 1H NMR and 13C NMR Spectra of Compoundsb1-29 in Supporting Information).

*N*-*Phenylacetourea (****b1****)*. White powder, 47%, mp: 110.9–112.1 °C, ^1^H NMR (600 MHz, CDCl_3_) *δ* (ppm): 3.71 (s, 2H, CH_2_); 7.28 (d, *J* = 6.8 Hz, 2H, Ar); 7.35 (t, *J* = 7.3 Hz, 1H, Ar); 7.39 (t, *J* = 7.3 Hz, 2H, Ar); 7.44 (s, 1H, NH_2_); 9.29 (s, 1H, NH_2_); 9.88 (s, 1H, NH); ^13^C NMR (150 MHz, CDCl_3_): 43.97; 128.06; 129.22; 129.38; 132.19; 171.72; 182.01; MS (ESI) *m/z* 195 (M + H)^+^; Anal. Calcd for C_9_H_10_N_2_OS: C, 55.65; H, 5.19; N, 14.42; S, 16.51; found: C, 55. 59; H, 5.19; N, 14.44; S, 16.53.

*N*-*(3-Bromophenylaceto)urea (****b2****).* White powder, 53%, mp: 207.4–208.8 °C, ^1^H NMR (600 MHz, CDCl_3_) *δ* (ppm): 3.58 (s, 2H, CH_2_); 6.98 (s, 1H, NH); 7.13 (dt, *J* = 7.3 Hz, *J* = 1.3 Hz, 1H, Ar); 7.20 (t, *J* = 7.8 Hz, 1H, Ar); 7.37 (t, *J* = 1.9 Hz, 1H, Ar); 7.42 (ddd, *J* = 8.0 Hz, *J* = 2.0 Hz, *J* = 1.1 Hz, 1H, Ar); 8.63 (s, 1H, NH_2_); 9.70 (s, 1H, NH_2_); ^13^C NMR (150 MHz, CDCl_3_): 42.21; 121.94; 129.04; 130.25; 130.95; 132.69; 137.54; 172.26; 182.04.

*N*-*(3,4-Dichlorophenylaceto)urea (****b3****).* White powder, 58%, mp: 202.1–203.4 °C, ^1^H NMR (600 MHz, DMSO-d_6_) *δ* (ppm): 3.75 (s, 2H, CH_2_); 7.28 (dd, *J* = 2.1 Hz, *J* = 10.3 Hz, 1H, Ar); 7.57 (d, *J* = 2.0 Hz, 1H, Ar); 7.59 (d, *J* = 8.0 Hz, 1H, Ar); 9.44 (s, 1H, NH_2_); 9.51 (s, 1H, NH_2_); 11.33 (s, 1H, NH); ^13^C NMR (150 MHz, DMSO-d_6_): 41.61; 130.12; 130.46; 130.87; 131.21; 132.08; 135.91; 171.9; 181.99.

*N*-*(3-Hydroxyphenylaceto)urea (****b4****).* White powder, 51%, mp: 160.3–161.5 °C, ^1^H NMR (600 MHz, DMSO-d_6_) *δ* (ppm): 3.60 (s, 2H, CH_2_); 6.65 (d, *J* = 7.1 Hz, 1H, Ar); 6.69 (d, *J* = 8.5 Hz, 1H, Ar); 6.70 (s, 1H, Ar); 7.10 (t, *J* = 7.8 Hz, 1H, Ar); 9.38 (s, 1H, OH); 9.41 (s, 1H, NH_2_); 9.58 (s, 1H, NH_2_); 11.26 (s, 1H, NH); ^13^C NMR (150 MHz, DMSO-d_6_): 42.87; 114.35; 116.60; 120.34; 129.80; 136.16; 157.77; 172.85; 182.18.

*N-(3-Methoxyphenylaceto)urea (****b5****)*. White powder, 59%, mp: 184.5–186.1 °C, ^1^H NMR (600 MHz, DMSO-d_6_) *δ* (ppm): 3.67 (s, 2H, CH_2_); 3.73 (s, 3H, OCH_3_); 6.83 (dd, *J* = 8.3 Hz, *J* = 2.8 Hz, 1H, Ar); 6.85 (d, *J* = 7.5 Hz, 1H, Ar); 6.88 (t, *J* = 1.8 Hz, 1H, Ar); 7.23 (td, *J* = 7.9 Hz, *J* = 1.6 Hz, 1H, Ar); 9.42 (s, 1H, NH_2_); 9.57 (s, 1H, NH_2_); 11.28 (s, 1H, NH); ^13^C NMR (150 MHz, DMSO-d_6_): 42.89; 55.44; 112.73; 115.63; 121.99; 129.87; 136.31; 159.66; 172.71; 182.16.

*N*-*(2-Fluorophenylaceto)urea (****b6****).* White powder, 60%, mp: 157.2–159.0 °C, ^1^H NMR (600 MHz, DMSO-d_6_) *δ* (ppm): 3.75 (s, 2H, CH_2_); 7.14 (t, *J* = 9.1 Hz, 1H, Ar); 7.19 (t, *J* = 7.5 Hz, 1H, Ar); 7.24 (s, 1H, NH_2_); 7.29 (t, *J* = 7.7 Hz, 1H, Ar); 7.36 (dd, *J* = 14.1 Hz, *J* = 7.2 Hz, 1H, NH_2_); 9.04 (s, 1H, NH_2_); 9.81 (s, 1H, NH); ^13^C NMR (150 MHz, DMSO-d_6_): 37.53; 115.88 (d, *J* = 21.5 Hz); 119.49 (d, *J* = 15.7 Hz); 124.80 (d, *J* = 3.7 Hz); 130.22 (d, *J* = 8.2 Hz); 131.55 (d, *J* = 3.6 Hz); 160.94 (d, *J* = 246.8 Hz); 170.31; 181.98.

*N*-*(3-Chlorophenylaceto)urea (****b7****).* White powder, 59%, mp: 136.6–137.4 °C, ^1^H NMR (600 MHz, DMSO-d_6_) *δ* (ppm): 3.74 (s, 2H, CH_2_); 7.25 (dt, *J* = 7.2 Hz, *J* = 1.6 Hz, 1H, Ar); 7.32–7.36 (m, 2H, Ar); 7.38 (t, *J* = 1.8 Hz, 1H, Ar); 9.44 (s, 1H, NH_2_); 9.54 (s, 1H, NH_2_); 11.32 (s, 1H, NH); ^13^C NMR (150 MHz, DMSO-d_6_): 42.26; 127.36; 128.66; 129.82; 130.64; 133.30; 137.26; 172.25; 182.05.

*N*-*(4-Hydroxyphenylaceto)urea (****b8****).* White powder, 57%, mp: 177.1–178.3 °C, ^1^H NMR (600 MHz, DMSO-d_6_) *δ* (ppm): 3.56 (s, 2H, CH_2_); 6.70 (d, *J* = 8.0 Hz, 1H, Ar); 7.07 (d, *J* = 8.0 Hz, 1H, Ar); 9.32 (s, 1H, OH); 9.39 (s, 1H, NH_2_); 9.58 (s, 1H, NH_2_); 11.20 (s, 1H, NH); ^13^C NMR (150 MHz, DMSO-d_6_): 40.03; 115.62; 124.96; 130.74; 156.77; 173.39; 182.23.

*N*-*(3-Trifluoromethylphenylaceto)urea (****b9****).* White powder, 51%, mp: 204.3–205.8 °C, ^1^H NMR (600 MHz, DMSO-d_6_) *δ* (ppm): 3.86 (s, 2H, CH_2_); 7.57 (d, *J* = 7.5 Hz, 1H, Ar); 7.60 (d, *J* = 7.8 Hz, 1H, Ar); 7.64 (d, *J* = 7.0 Hz, 1H, Ar); 7.68 (s, 1H, Ar); 9.44 (s, 1H, NH_2_); 9.53 (s, 1H, NH_2_); 11.36 (s, 1H, NH); ^13^C NMR (150 MHz, DMSO-d_6_): 42.28; 124.12 (q, *J* = 3.8 Hz); 124.67 (q, *J* = 272.2 Hz); 126.63 (q, *J* = 3.8 Hz); 129.46 (q, *J* = 31.5 Hz); 129.82; 134.18; 136.23; 172.22; 182.03.

*N*-*(3,4-Dimethoxyphenylaceto)urea (****b10****).* White powder, 50%, mp: 146.3–147.6 °C, ^1^H NMR (600 MHz, DMSO-d_6_) *δ* (ppm): 3.61 (s, 2H, CH_2_); 3.72 (s, 3H, OCH_3_); 3.73 (s, 3H, OCH_3_); 6.81 (dd, *J* = 8.2 Hz, *J* = 2.0 Hz, 1H, Ar); 6.89 (d, *J* = 8.3 Hz, 1H, Ar); 6.90 (d, *J* = 2.0 Hz, 1H, Ar); 9.40 (s, 1H, NH_2_); 9.58 (s, 1H, NH_2_); 11.23 (s, 1H, NH); ^13^C NMR (150 MHz, DMSO-d_6_): 42.44; 55.89; 55.96; 112.26; 113.65; 121.87; 127.15; 148.29; 148.98; 173.10; 182.19.

*N*-*(4-Methoxyphenylaceto)urea (****b11****).* White powder, 56%, mp: 143.5–144.7 °C, ^1^H NMR (600 MHz, DMSO-d_6_) *δ* (ppm): 3.62 (s, 2H, CH_2_); 3.73 (s, 3H, OCH_3_); 6.89 (d, *J* = 8.6 Hz, 2H, Ar); 7.21 (d, *J* = 8.6 Hz, 2H, Ar); 9.40 (s, 1H, NH_2_); 9.58 (s, 1H, NH_2_); 11.25 (s, 1H, NH); ^13^C NMR (150 MHz, DMSO-d_6_): 41.98; 55.49; 114.28; 126.76; 130.84; 158.72; 173.22; 182.21.

*N*-*(4-Bromophenylaceto)urea (****b12****).* White powder, 53%, mp: 174.9–176.2 °C, ^1^H NMR (600 MHz, CDCl_3_) *δ* (ppm): 3.56 (s, 2H, CH_2_); 6.99 (s, 1H, NH); 7.07 (d, *J* = 8.3 Hz, 2H, Ar); 7.44 (d, *J* = 8.3 Hz, 2H, Ar); 8.67 (s, 1H, NH_2_); 9.70 (s, 1H, NH_2_); ^13^C NMR (150 MHz, CDCl_3_): 43.41; 122.37; 131.04; 131.14; 132.42; 170.59; 181.93.

*N*-*(2-Chlorophenylaceto)urea (****b13****).* White powder, 55%, mp: 148.8–151.2 °C, ^1^H NMR (600 MHz, DMSO-d_6_) *δ* (ppm): 3.91 (s, 2H, CH_2_); 7.29–7.32 (m, 2H, Ar); 7.37–7.40 (m, 1H, Ar); 7.42–7.45 (m, 1H, Ar); 9.42 (s, 1H, NH_2_); 9.52 (s, 1H, NH_2_); 11.38 (s, 1H, NH); ^13^C NMR (150 MHz, DMSO-d_6_): 40.84; 127.60; 129.44; 129.47; 132.92; 133.04; 134.17; 171.73; 182.01; MS (ESI) *m/z* 229 (M + H)^+^; Anal. Calcd for C_9_H_9_ClN_2_OS: C, 47.27; H, 3.97; Cl, 15.50; N, 12.25; S, 14.02; found: C, 47.21; H, 3.97; Cl, 15.52; N, 12.25; S, 14.03.

*N*-*(3-Fluorophenylaceto)urea (****b14****).* White powder, 58%, mp: 126.0–127.4 °C, ^1^H NMR (600 MHz, DMSO-d_6_) *δ* (ppm): 3.75 (s, 2H, CH_2_); 6.95–7.23 (m, 3H, Ar); 7.30–7.52 (m, 1H, Ar); 9.43 (s, 1H, NH_2_); 9.54 (s, 1H, NH_2_); 11.32 (s, 1H, NH); ^13^C NMR (150 MHz, DMSO-d_6_): 42.37 (d, *J* = 1.8 Hz); 114.20 (d, *J* = 20.8 Hz); 116.74 (d, *J* = 21.4 Hz); 126.07 (d, *J* = 2.7 Hz); 130.67 (d, *J* = 8.3 Hz); 137.52 (d, *J* = 8.0 Hz); 162.45 (d, *J* = 243.2 Hz); 172.28; 182.07.

*N*-*(2-Bromophenylaceto)urea (****b15****).* White powder, 54%, mp: 179.0–179.5 °C, ^1^H NMR (600 MHz, DMSO-d_6_) *δ* (ppm): 3.92 (s, 2H, CH_2_); 7.22 (td, *J* = 7.7 Hz, *J* = 1.7 Hz, 1H, Ar); 7.35 (td, *J* = 7.4 Hz, *J* = 1.8 Hz, 1H, Ar); 7.38 (dd, *J* = 7.6 Hz, *J* = 1.9 Hz,1H, Ar); 7.60 (dd, *J* = 8.6 Hz, *J* = 2.3 Hz, 1H, Ar); 9.41 (s, 1H, NH_2_); 9.52 (s, 1H, NH_2_); 11.38 (s, 1H, NH); ^13^C NMR (150 MHz, DMSO-d_6_): 43.28; 125.06; 128.14; 129.63; 132.72; 133.00; 134.83; 171.69; 182.01.

*N*-*(4-Methylphenylaceto)urea (****b16****).* White powder, 55%, mp: 171.2–172.5 °C, ^1^H NMR (600 MHz, DMSO-d_6_) *δ* (ppm): 2.27 (s, 3H, CH_3_); 3.65 (s, 2H, CH_2_); 7.12 (d, *J* = 7.9 Hz, 2H, Ar); 7.17 (d, *J* = 7.9 Hz, 2H, Ar); 9.40 (s, 1H, NH_2_); 9.57 (s, 1H, NH_2_); 11.26 (s, 1H, NH); ^13^C NMR (150 MHz, DMSO-d_6_): 21.13; 42.45; 129.41; 129.66; 131.84; 136.44; 173.04; 182.19.

*N*-*(2-Methoxyphenylaceto)urea (****b17****).* White powder, 61%, mp: 135.4–136.9 °C, ^1^H NMR (600 MHz, DMSO-d_6_) *δ* (ppm): 3.69 (s, 2H, CH_2_); 3.75 (s, 3H, OCH_3_); 6.89 (td, *J* = 7.4 Hz, *J* = 1.0 Hz, 1H, Ar); 6.97 (dd, *J* = 8.2 Hz, *J* = 2.8 Hz, 1H, Ar); 7.17 (dd, *J* = 7.5 Hz, *J* = 1.7 Hz, 1H, Ar); 7.25 (td, *J* = 7.8 Hz, *J* = 1.5 Hz, 1H, Ar); 9.36 (s, 1H, NH_2_); 9.57 (s, 1H, NH_2_); 11.17 (s, 1H, NH); ^13^C NMR (150 MHz, DMSO-d_6_): 37.94; 55.90; 111.16; 120.66; 123.22; 128.94; 131.69; 157.66; 172.95; 182.10.

*N*-*(2-Methylphenylaceto)urea (****b18****).* White powder, 60%, mp: 135.6–137.1 °C, ^1^H NMR (600 MHz, DMSO-d_6_) *δ* (ppm): 2.23 (s, 3H, CH_3_); 3.76 (s, 2H, CH_2_); 7.11–7.15 (m, 1H, Ar); 7.15–7.17 (m, 2H, Ar); 7.19 (dd, *J* = 6.7 Hz, *J* = 1.7 Hz, 1H, Ar); 9.41 (s, 1H, NH_2_); 9.57 (s, 1H, NH_2_); 11.29 (s, 1H, NH_2_); ^13^C NMR (150 MHz, DMSO-d_6_): 19.74; 40.69; 126.30; 127.52; 130.41; 130.68; 133.67; 137.20; 172.80; 182.11.

*N*-*(4-Chlorophenylaceto)urea (****b19****).* White powder, 57%, mp: 184.1–186.6 °C, ^1^H NMR (600 MHz, DMSO-d_6_) *δ* (ppm): 3.72 (s, 2H, CH_2_); 7.31 (d, *J* = 8.4 Hz, 2H, Ar); 7.39 (d, *J* = 8.4 Hz, 2H, Ar); 9.43 (s, 1H, NH_2_); 9.54 (s, 1H, NH_2_); 11.31 (s, 1H, NH); ^13^C NMR (150 MHz, DMSO-d_6_): 42.02; 128.77; 131.77; 132.12; 133.88; 172.48; 182.09.

*N*-*(2-Nitrophenylaceto)urea (****b20****).* White powder, 59%, mp: 177.6–178.4 °C, ^1^H NMR (600 MHz, DMSO-d_6_) *δ* (ppm): 4.17 (s, 2H, CH_2_); 7.55 (dd, *J* = 7.6, *J* = 1.4 Hz, 1H, Ar); 7.59 (td, *J* = 7.8, *J* = 1.5 Hz, 1H, Ar); 7.73 (td, *J* = 7.5, *J* = 1.3 Hz, 1H, Ar); 8.10 (dd, *J* = 8.2, *J* = 1.3 Hz, 1H, Ar); 9.41 (s, 2H, NH_2_); 11.41 (s, 1H, NH); ^13^C NMR (150 MHz, DMSO-d_6_): 41.21; 125.24; 129.32; 129.88; 134.41; 134.45; 148.92; 171.52; 181.93.

*N*-*(2,4-Dichlorophenylaceto)urea (****b21****).* White powder, 59%, mp: 166.6–167.2 °C, ^1^H NMR (600 MHz, DMSO-d_6_) *δ* (ppm): 3.92 (s, 2H, CH_2_); 7.41 (dd, *J* = 8.3 Hz, *J* = 2.0 Hz, 1H, Ar); 7.43 (d, *J* = 8.2 Hz, 1H, Ar); 7.61 (d, *J* = 2.2 Hz, 1H, Ar); 9.43 (s, 1H, NH_2_); 9.48 (s, 1H, NH_2_); 11.40 (s, 1H, NH); ^13^C NMR (150 MHz, DMSO-d_6_): 40.23; 127.74; 128.94; 132.23; 133.08; 134.19; 135.17; 171.32; 181.93.

*N-(4-Nitrophenylaceto)urea (****b22****).* White powder, 58%, mp: 199.1–200.9 °C, ^1^H NMR (600 MHz, DMSO-d_6_) *δ* (ppm): 3.91 (s, 2H, CH_2_); 7.57 (d, *J* = 8.7 Hz, 2H, Ar); 8.20 (d, *J* = 8.7 Hz, 2H, Ar); 9.46 (s, 1H, NH_2_); 9.50 (s, 1H, NH_2_); 11.40 (s, 1H, NH); ^13^C NMR (150 MHz, DMSO-d_6_): 42.43; 123.86; 131.38; 142.81; 147.04; 171.72; 181.99.

*N*-*(4-Fluorophenylaceto)urea (****b23****).* White powder, 57%, mp: 165.4–166.5 °C, ^1^H NMR (600 MHz, DMSO-d_6_) *δ* (ppm): 3.71 (s, 2H, CH_2_); 7.15 (t, *J* = 8.9 Hz, 2H, Ar); 7.32 (dd, *J* = 8.5 Hz, *J* = 5.7 Hz, 2H, Ar); 9.42 (s, 1H, NH_2_); 9.55 (s, 1H, NH_2_); 11.30 (s, 1H, NH); ^13^C NMR (150 MHz, DMSO-d_6_): 41.87; 115.57 (d, *J* = 21.1 Hz); 131.04 (d, *J* = 3.2 Hz); 131.78 (d, *J* = 8.0 Hz); 161.74 (d, *J* = 242.7 Hz); 172.75; 182.12.

*N*-*(2-Naphthylaceto)urea (****b24****).* White powder, 52%, mp: 202.6–204.4 °C, ^1^H NMR (600 MHz, DMSO-d_6_) *δ* (ppm): 3.90 (s, 1H); 7.45 (dd, *J* = 8.4 Hz, *J* = 1.7 Hz, 1H, Ar); 7.49 (dd, *J* = 6.8 Hz, *J* = 1.6 Hz, 1H, Ar); 7.51 (dd, *J* = 7.0 Hz, *J* = 1.8 Hz, 1H, Ar); 7.81 (d, *J* = 1.8 Hz, 1H, Ar); 7.88 (d, *J* = 8.1 Hz, 2H, Ar); 7.89 (d, *J* = 8.7 Hz, 1H, Ar); 9.45 (s, 1H, NH_2_); 9.60 (s, 1H, NH_2_); 11.40 (s, 1H, NH); ^13^C NMR (150 MHz, DMSO-d_6_): 42.96; 126.28; 126.68; 127.94; 127.98; 128.18; 128.29; 128.34; 132.41; 132.59; 133.38; 172.85; 182.17.

*N*-*(Diphenylaceto)urea (****b25****).* White powder, 54%, mp: 169.5–172.7 °C, ^1^H NMR (600 MHz, DMSO-d_6_) *δ* (ppm): 5.40 (s, 1H, CH); 7.23–7.32 (m, 6H, Ar); 7.33–7.38 (m, 4H, Ar); 9.52 (s, 1H, NH_2_); 9.61 (s, 1H, NH_2_); 11.49 (s, 1H, NH); ^13^C NMR (150 MHz, DMSO-d_6_): 56.77; 127.70; 129.03; 129.04; 139.00; 173.35; 182.12.

*N-(Biphenyl-4-yl)acetourea (****b26****).* White powder, 52%, mp: 187.3–189.6 °C, ^1^H NMR (600 MHz, DMSO-d_6_) *δ* (ppm): 3.76 (s, 2H); 7.36 (t, *J* = 7.4 Hz, 1H, Ar); 7.39 (d, *J* = 8.1 Hz, 2H, Ar); 7.46 (t, *J* = 7.7 Hz, 2H, Ar); 7.62 (d, *J* = 8.1 Hz, 2H, Ar); 7.65 (d, *J* = 6.8 Hz, 2H, Ar); 9.44 (s, 1H, NH_2_); 9.59 (s, 1H, NH_2_); 11.35 (s, 1H, NH); ^13^C NMR (150 MHz, DMSO-d_6_): 42.49; 127.07; 127.18; 127.85; 129.38; 130.41; 134.15; 139.31; 140.33; 172.83; 182.18.

*N-(1-Naphthylaceto)urea (****b27****).* White powder, 57%, mp: 195.5–197.9 °C, ^1^H NMR (600 MHz, DMSO-d_6_) *δ* (ppm): 4.24 (s, 2H, CH_2_); 7.46–7.49 (m, 2H, Ar); 7.53 (td, *J* = 7.4 Hz, *J* = 1.2 Hz, 1H, Ar); 7.57 (td, *J* = 8.3 Hz, *J* = 1.5 Hz, 1H, Ar); 7.84–7.89 (m, 1H, Ar); 7.94 (dd, *J* = 8.1, *J* = 1.4 Hz, 1H); 8.02 (d, *J* = 7.8 Hz, 1H); 9.43 (s, 1H, NH_2_); 9.54 (s, 1H, NH_2_); 11.48 (s, 1H, NH); ^13^C NMR (150 MHz, DMSO-d_6_): 40.20; 124.51; 125.99; 126.24; 126.74; 128.07; 128.74; 128.95; 131.49; 132.32; 133.81; 172.78; 182.12.

*N-(2-(benzo[d][1,3]dioxol-5-yl)aceto)urea (****b28****).* White powder, 56%, mp: 153.5–155.0 °C, ^1^H NMR (600 MHz, DMSO-d_6_) *δ* (ppm): 3.60 (s, 2H, CH_2_); 5.99 (s, 2H, OCH_2_O); 6.75 (dd, *J* = 8.0 Hz, *J* = 1.7 Hz, 1H, Ar); 6.85 (d, *J* = 7.7 Hz, 1H, Ar); 6.86 (d, *J* = 1.5 Hz, 1H, Ar); 9.41 (s, 1H, NH_2_); 9.56 (s, 1H, NH_2_); 11.23 (s, 1H, NH); ^13^C NMR (150 MHz, DMSO-d_6_): 42.43; 101.34; 108.60; 110.21; 122.95; 128.40; 146.64; 147.63; 172.95; 182.16.

*N-(3,4-Dihydroxyphenylaceto)urea (****b29****).* White powder, 56%, mp: 153.5–155.0 °C, ^1^H NMR (600 MHz, DMSO-d_6_) *δ* (ppm): 3.48 (s, 2H, CH_2_); 6.53 (dd, *J* = 8.1 Hz, *J* = 2.1 Hz, 1H, Ar); 6.65 (d, *J* = 8.0 Hz, 1H, Ar); 6.68 (d, *J* = 2.1 Hz, 1H, Ar); 8.79 (s, 1H, OH); 8.88 (s, 1H, OH); 9.39 (s, 1H, NH_2_); 9.60 (s, 1H, NH_2_); 11.17 (s, 1H, NH); ^13^C NMR (150 MHz, DMSO-d_6_): 42.29; 115.90; 117.04; 120.52; 125.52; 144.77; 145.53; 173.40; 182.25.

## Results and discussion

### Chemistry

**Scheme 1.**
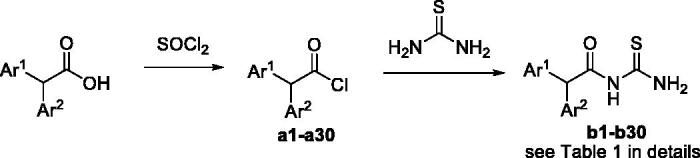
Synthesis of arylacetothioureas.

A typical synthetic route towards arylacetothioureas is described in Scheme 1. The arylacetyl chloride intermediate was synthesised from an appropriate arylacetic acid by reaction with SOCl_2_. Amidation was followed with thiourea to afford the corresponding compound **b**.

### Inhibitory activity against cell-free urease

All synthesised arylacetothioureas were evaluated for their inhibitory activity against extracted *H. pylori* urease. As shown in Table 1, a compound bearing naphthyl (**b24** and **b27**), benzo-1,3-dioxole (**b28**) or biphenyl (**b26**) moiety exhibited very low potency, even showed inactivity. In contrast, some compounds from phenylacetothioureas showed excellent potency with IC_50_ values lower than that of the positive control AHA. Out of these compounds, **b19** was the most active inhibitor with IC_50_ of 0.16 ± 0.05 µM, showing 158-fold more potency than AHA. Replacement of the chloro group of compound **b19** by other substitutions such as fluoro, bromo, methyl, and methoxy attenuated urease inhibition by 27- to 968-fold, which suggested that only suitable steric volume is tolerated. Movement of the chlorine from the *para* position (**b19**) to the *meta* position (**b7**) produced a 397-fold decrease in potency. In the case of *meta*-substituted derivatives, a different result was observed. Replacement of chloro group with fluoro (**b14**), bromo (**b2**), or methoxy (**b5**) group gives a comparable activity. However, hydroxyl-substituted analogue (**b4**) showed an about fourfold increase in potency in comparison with **b7**, which may be attributed to a possible hydrogen-bond building ability of the hydroxyl group. In comparison with the *meta*-substituted derivatives, the *ortho*-analogous resulted in no significant change in potency. It is to be noted that compound containing a strong electron-withdrawing nitro group (**b20**) resulted in a significant decrease in potency with IC_50_ value over 180 µM. In the case of double substituent on the phenyl ring, compound with 3,4-dihyroxyl group resulted in IC_50_ values of 18.4 µM, being the most active in this series and showing more potent than the positive control AHA.

**Table 1.**
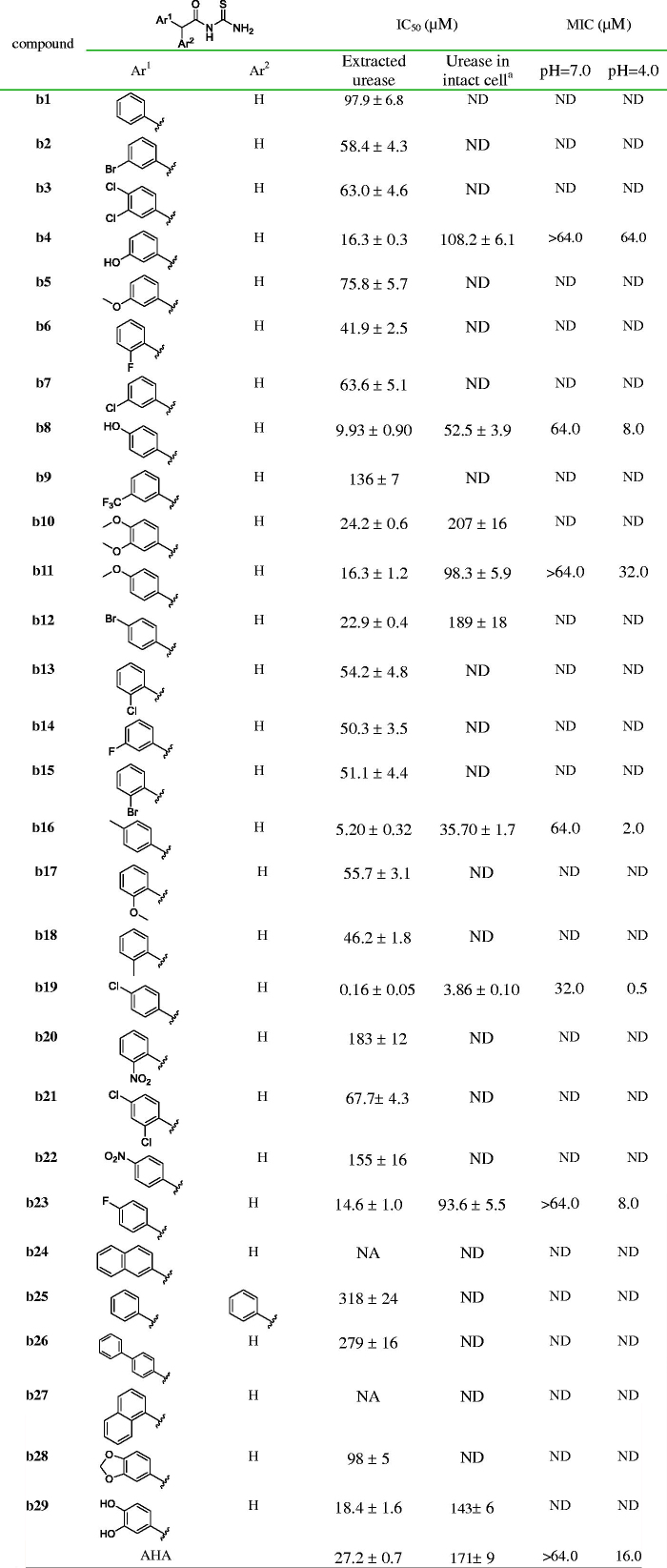
Structure, inhibitory activity (IC_50_), antibacterial activity (MIC_50_) against *H. pylori* urease of compounds **b1**–**b29**.

^a^ ND: no determination.

### Inhibitory activity against urease in intact cell

Encouraged by the results of the extracted urease assay, compounds showing higher potency than AHA were selected to determine inhibitory activities against urease in intact *H. pylori* cell, and the results are shown in Table 1. A 5- to 24-fold increase of IC_50_ values against urease in intact cell was observed in comparison with those of extracted urease. Three compounds (**b8**, **b16**, and **b19**) were found to be much more potent than AHA, showing IC_50_ of 52.5 ± 3.9, 35.7 ± 1.7, and 3.86 ± 0.10 µM. Not surprisingly, the most potent compound **b19** found in the extracted urease assay was also exhibited the highest activity against urease in intact *H. pylori* cell.

### Antibacterial activity

To get an insight of the possibility of the identified urease inhibitors for further drug developing, compounds showing higher potency in enzyme assays than AHA were selected to test the potential growth inhibition against *H. pylori*, and the results are shown in Table 1. The assayed compounds showed no or very weak antibacterial activity against *H. pylori* at neutral pH. However, at low pH (about 4.0), compounds with significant inhibition against urease in intact *H. pylori* cell showed impressive antibacterial effect against *H. pylori*. This is consistent with that revealed by Eaton et al. and Karita et al. as above mentioned^4^^,^^5^. In general, these data indicated a potential for *in vivo* efficacy of compounds such as **b19** and **b16** in clearing *H. pylori* infection.

### Ligand affinity

**Figure 1.**
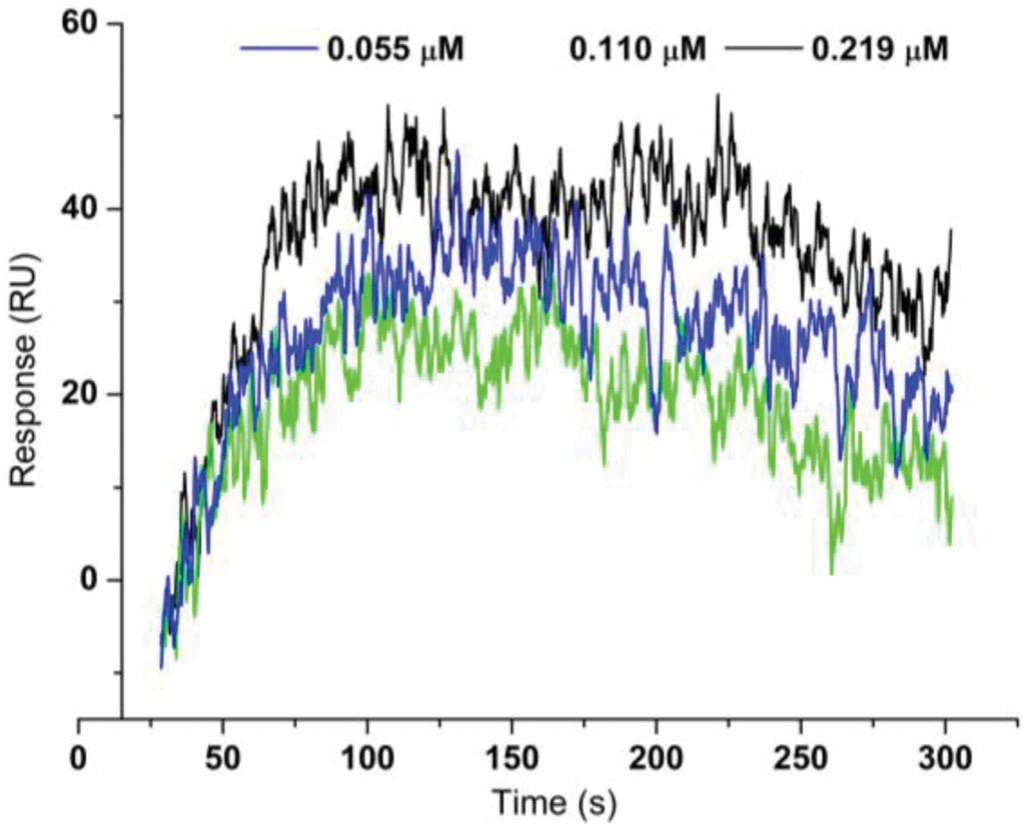
Sensograms of interactions between **b19** and urease.

Compounds with IC_50_ lower than 20 µM against extracted *H. pylori* urease were selected for ligand-enzyme binding interaction based on SPR, which allows for the determination of affinities and kinetic parameters in a single experiment^25^. In the present paper, the pure Jack bean urease from commercial was used for this assay because of non-availability of pure *H. pylori* urease. The binding affinities are shown in Table 2 and the representative SPR plots are shown in Figure 1. The most active compound **b19** displayed the highest binding affinity to urease with a *K*_D_ value of 4.50 ± 0.16 nM. For efficient and tight ligand binding, the rate constant *k*_off_ is of particular interest, because *k*_off_ has a potential to differentiate indistinguishable compounds with similar affinities^26^. The results revealed that **b19** displayed very slow dissociation from the catalytic domain of urease with a *k*_off_ 1.73 × 10^−3^ s^−1^, indicating that **b19** could be prioritised for further optimisation.

**Table 2.** The binding affinity data for urease–thioureas interactions.

Interaction	*K*_on_ (M^–1^·s^–1^)	*K*_off_ (s^–1^)	*K*_D_ (nM)
Urease–**b4**	6.08 × 10^4^ ± 2.26 × 10^2^	3.89 × 10^–2^ ± 2.93 × 10^–4^	644.0 ± 9.2
Urease–**b8**	9.38 × 10^6^ ± 3.18 × 10^4^	2.92 × 10^–3^ ± 0.61 × 10^–5^	311.2 ± 10.3
Urease–**b11**	5.72 × 10^5^ ± 4.35 × 10^3^	3.81 × 10^–2^ ± 7.60 × 10^–4^	666.7 ± 13.7
Urease–**b16**	5.01 × 10^5^ ± 6.09 × 10^3^	8.10 × 10^–2^ ± 1.38 × 10^–4^	162.0 ± 3.8
Urease–**b19**	3.82 × 10^6^ ± 5.79 × 10^4^	1.73 × 10^–3^ ± 2.57 × 10^–5^	4.50 ± 0.16
Urease–**b23**	5.43 × 10^4^ ± 3.92 × 10^2^	3.09 × 10^–2^ ± 1.81 × 10^–4^	569.2 ± 21.6
Urease–**b29**	1.98 × 10^4^ ± 1.75 × 10^2^	1.55 × 10^–2^ ± 0.44 × 10^–4^	782.5 ± 21.3

### Inhibition kinetics

**Figure 2.**
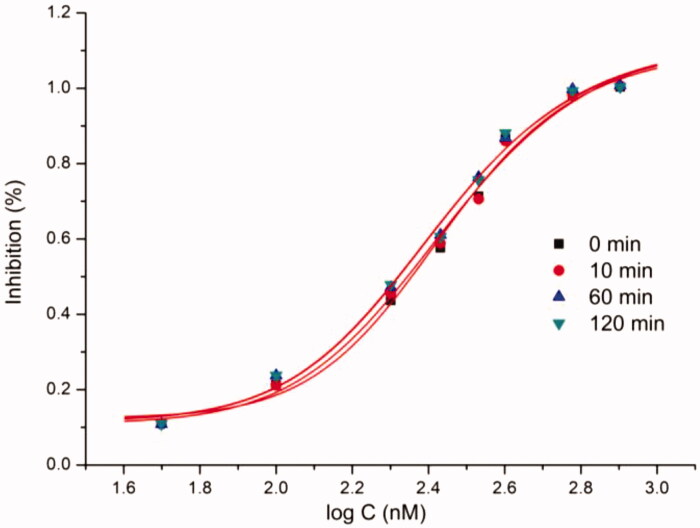
Characterisation of urease inhibition by compound **b19** for different preincubation time.

Compounds with IC_50_ lower than 20 µM against extracted *H. pylori* urease were selected to perform kinetic assay for further insight of the inhibition mechanism. Mazzei et al. confirmed that urease shared the mechanism of catalysis and inhibition regardless of the biological sources^27^^,^^28^. For excluding the possible interference, a pure urease (Jack bean urease) was therefore used for kinetic assays. As an example, Figure 2 describes the preincubation-time dependence of urease inhibition by **b19**. The suppressed urease activity maintained at relatively constant values with the increasing preincubation time under different concentrations of **b19**, resulting in nearly equal IC_50_ values. The obtained results indicate that **b19** can rapidly bind to the active site and inhibit urease in a time-independent manner.

**Figure 3.**
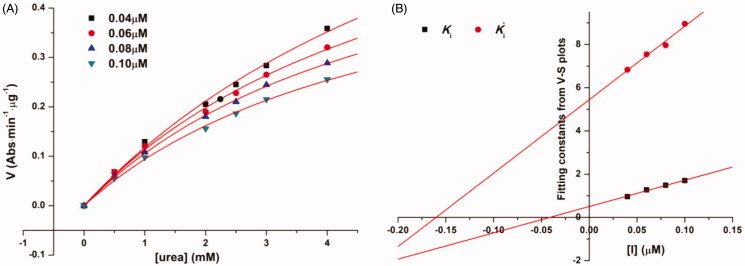
A velocity (*V*) was nonlinearly fitted against the concentrations of urea [*S*] in the presence of a specific concentration of compound **b19**. (B) (1) $K_{i}:$ the fitted constants ($\frac{K_{m}}{V_{\max}}(1+\frac{\left[I\right]}{K_{i}})$) from the corresponding *V*–*S* plot were plotted against concentrations of **b19** ([*I*]); (2) $K_{i}^{'}:$ the fitted constants ($\frac{1}{V_{\max}}(1+\frac{\left[I\right]}{K_{i}^{\prime }})$) from the corresponding *V*–*S* plot were plotted against concentrations of **b19**.

Figure 3 shows the nonlinear fitting curves (*V* vs. [*S*]) of the most potential urease inhibitor **b19** based on the general kinetics equation (Equation (1), and the corresponding plots by linearly fitting the fitting constants from *V*–[*S*] curves against [*I*], which provided the kinetic parameters $K_{i}$ and $K_{i}^{'}$ of **b19**. Herein, $K_{i}$ is the dissociation constant for “urease-inhibitor → urease and inhibitor” and $K_{i}^{'}$ is the dissociation constant for “urease-urea-inhibitor → urease-urea and inhibitor”^1^. The calculated $K_{i}$ and $K_{i}^{'}$ of **b19** are 0.040 and 0.16 µM, respectively, suggesting: (1) **b19** is a reversible urease inhibitor ($K_{i}\to \infty$ and $K_{i}^{\prime }\to \infty$ indicate an irreversible inhibition); (2) **b19** has a mixed competitive mechanism ($K_{i}$ and $K_{i}^{'}$ are not →∞, and $K_{i}\;\neq \;K_{i}^{'}$). Similarly, kinetic parameters ($K_{i}$ and $K_{i}^{'}$) and inhibition types of other compounds were also determined and are shown in Table 3. The values of $K_{i}^{'}$ are larger than the corresponding $K_{i}$ for all tested compounds, suggesting that the complex of urease-urea-inhibitor is less stable than that of the urease-inhibitor and competitive inhibition has relatively higher weight in the mixed competitive mechanism.

**Table 3.** Data of inhibition mechanism.

Compound	Type of inhibition	$K_{i}$ (μM)	$K_{i}^{'}$ (μM)
**b4**	Reversible	4.34	8.52
**b8**	Reversible	2.54	11.3
**b11**	Reversible	3.94	12.7
**b16**	Reversible	1.28	2.84
**b19**	Reversible	0.040	0.16
**b23**	Reversible	3.53	9.67
**b29**	Reversible	8.21	21.5

### Molecular docking

**Figure 4.**
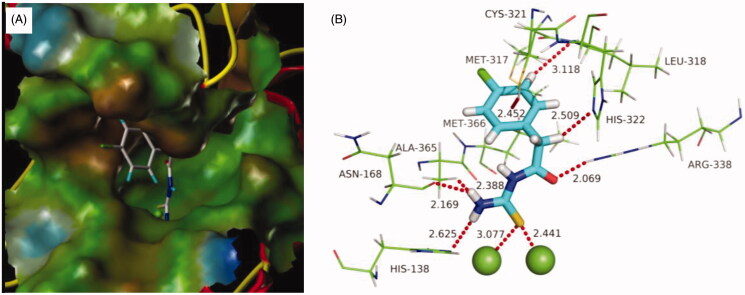
Predicted binding mode of ligand-urease (PDB code: 1e9y): (A) compound **b19** shown as white sticks and the enzyme shown as surface. (B) Compound **b19** shown as cyan sticks and enzyme shown as lines; Hydrogen bonds shown as red dashed lines.

With the aim to explore the structural determinants of the guidance for further SAR studies, molecular docking of the most potent inhibitor **b19** into urea binding site was performed, and the binding model is depicted in Figure 4. This model revealed that the monosubstituted thiourea moiety is nicely bound to urea binding site (Figure 4(A)), and is of primary importance for its network of interactions (Figure 4(B)): it coordinates the nickel ion and establishes hydrogen bonds with N of His138, O of Asn168, and Me of Ala365, respectively. On the other hand, the benzene ring of **b19** establishes appropriate hydrophobic contacts with the hydrophobic gap (Met317, Leu318, and Met366) under the movable flap, a helix-turn-helix motif composed of residues α313–α346. Moreover, benzene ring is further solid by an S–H···π with Cys321, and a C–H···N with Leu318. In addition, this model also suggested that the aceto moiety as a donor as well as an acceptor forms C–H···N and N–H···O hydrogen bonds with His322 and Arg338, respectively. The enzyme assay data and the molecular docking results indicated that compound **b19** is a potential inhibitor of urease.

### Molecular dynamics

**Figure 5.**
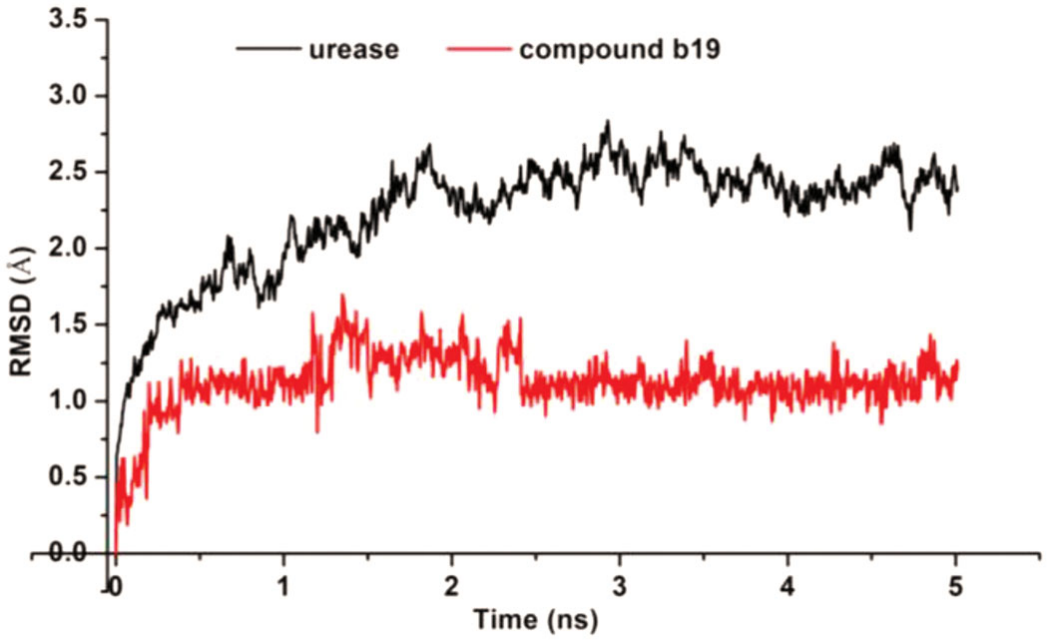
The RMSD values obtained during 6 ns of molecular dynamic simulation for urease and **b19**.

Molecular dynamic (MD) simulations were performed to understand the dynamic properties of urease–**b19** complexes, and to provide some evidence for the suppression site identified by molecular dockings. Figure 5 shows the evolutions of the root mean square deviation (RMSD) values versus time in reference to the energy minimised complex structure. RMSD change suggested that simulation attains an equilibrium position within 1 ns, and the average RMSD fluctuation values of **b19** were 1.1 Å after equilibrium was reached. The very low deviation from docked position indicated that molecular docking results of **b19** are reliable, which was also evidenced by the high binding affinity observed in enzyme and SPR assays.

### Cytotoxicity

The highly bioactive compounds with IC_50_ value against urease lower than that of AHA were further evaluated for its toxicity profile against human normal hepatic cell line L-02 and normal prostate cell line P69 using the colorimetric cell proliferation MTT assay. As shown in Table 4, most of the assayed compounds showed low cytotoxicity against these human normal cell lines with viability over 90% at concentration of 25 µg/mL. It is to be noted that the cell viability against the most potent compound **b19** of the two cell lines was more than 93%, suggesting the low toxicity to mammalian cells. On the whole, the new identified urease inhibitor **b19** showed lower cell toxicity than the positive control AHA.

**Table 4.** Cell Viability of selected compounds on L-02 and P69 at concentration of 25 μg/mL.

Compound	Viability (%)	
	L-02	P69
**b4**	93.7	88.1
**b8**	92.1	94.6
**b10**	108.1	95.3
**b11**	97.0	87.5
**b12**	91.5	89.4
**b16**	113.8	89.9
**b19**	96.8	93.6
**b23**	96.1	97.6
AHA	80.2	71.2

## Conculsions

In summary, we have developed a series of mono-substituted thioureas as potent *H. pylori* urease inhibitors. The most potent compound **b19** was identified as a reversible urease inhibitor, and significantly inhibits extracted urease and urease in intact cell with IC_50_ values of 0.16 ± 0.05 and 3.86 ± 0.10 µM, being 170- and 44-fold more potent than clinically used drug AHA, respectively. The SPR assay thus revealed that **b19** exhibits very high urease affinities in the low nM range, probably binding to the urea site and showing very slow dissociation from the catalytic domain. Urease inhibition assay, SPR assay, molecular docking studies and cell proliferation assay suggested that the mono-substituted thioureas are a new kind of urease inhibitors acting at the urea site, with **b19** having potential for further development as an agent to treat *H. pylori* caused diseases.

## Supplementary Material

Supplemental Material
