# Supplementary figures and images for: N-monoarylacetothioureas as potent urease inhibitors: synthesis, SAR, and biological evaluation
Source: J Enzyme Inhib Med Chem. 2019 Dec 27;35(1):404–13. doi: 10.1080/14756366.2019.1706503 (PMC6968641; doi:10.1080/14756366.2019.1706503)

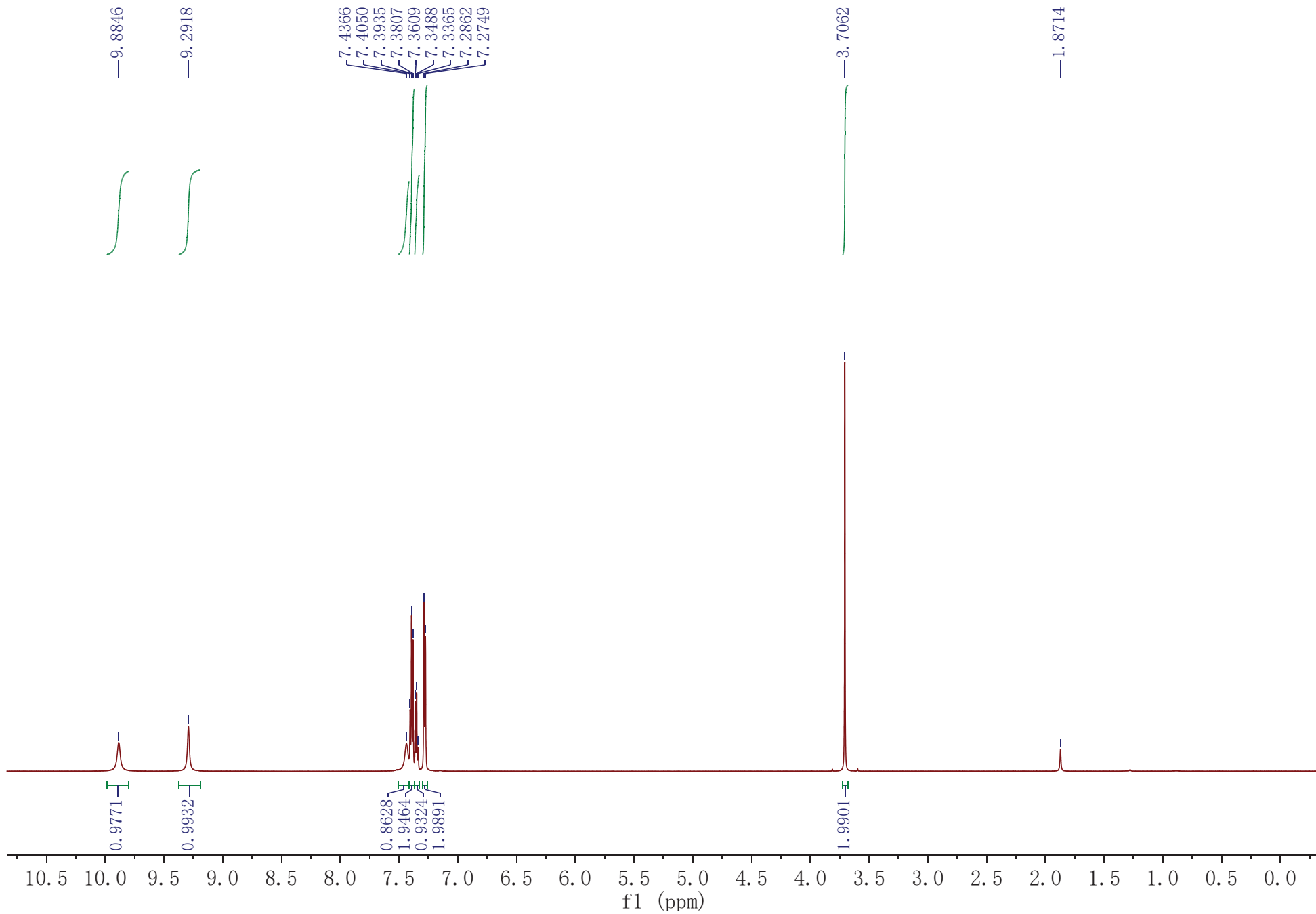

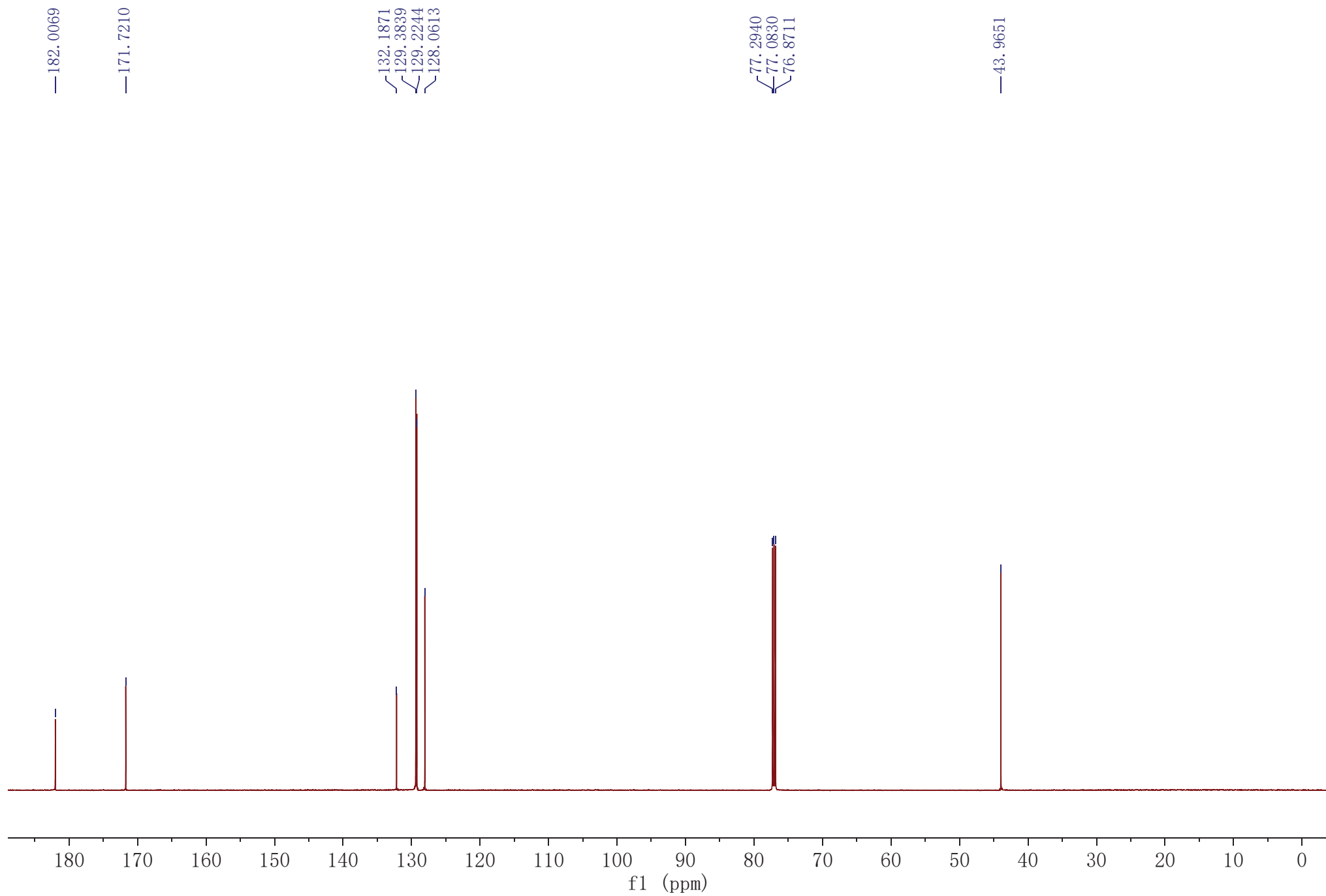

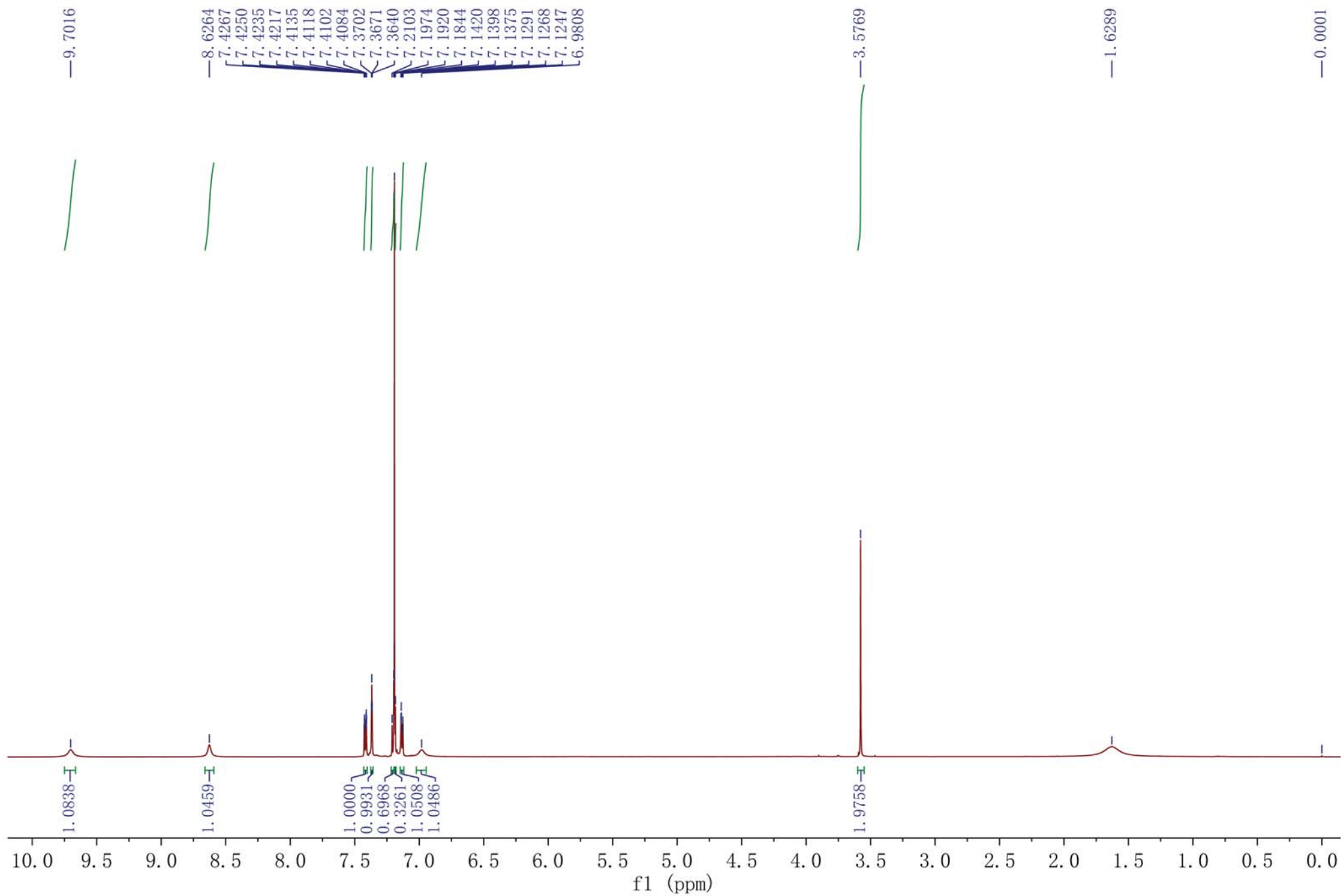

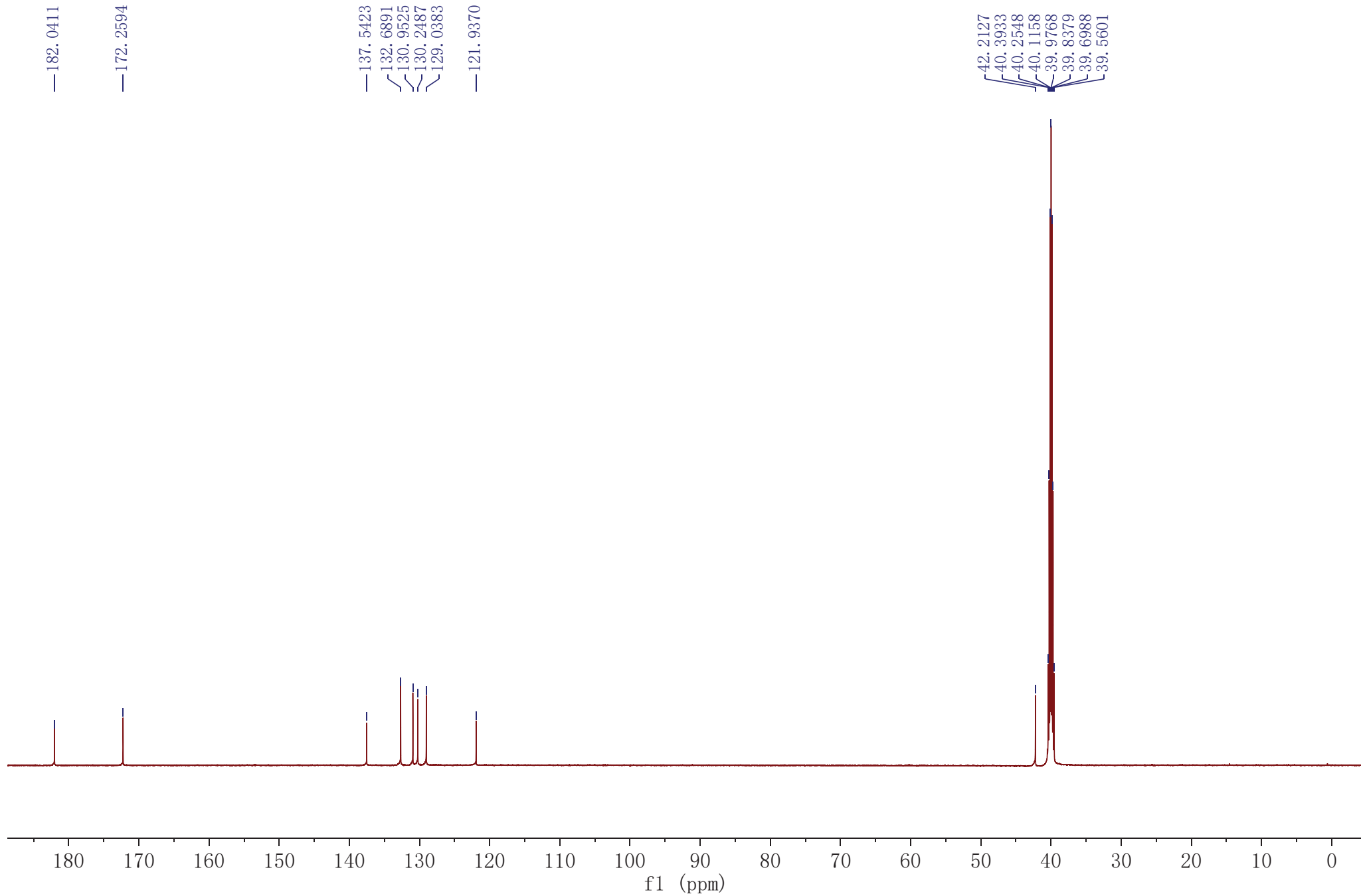

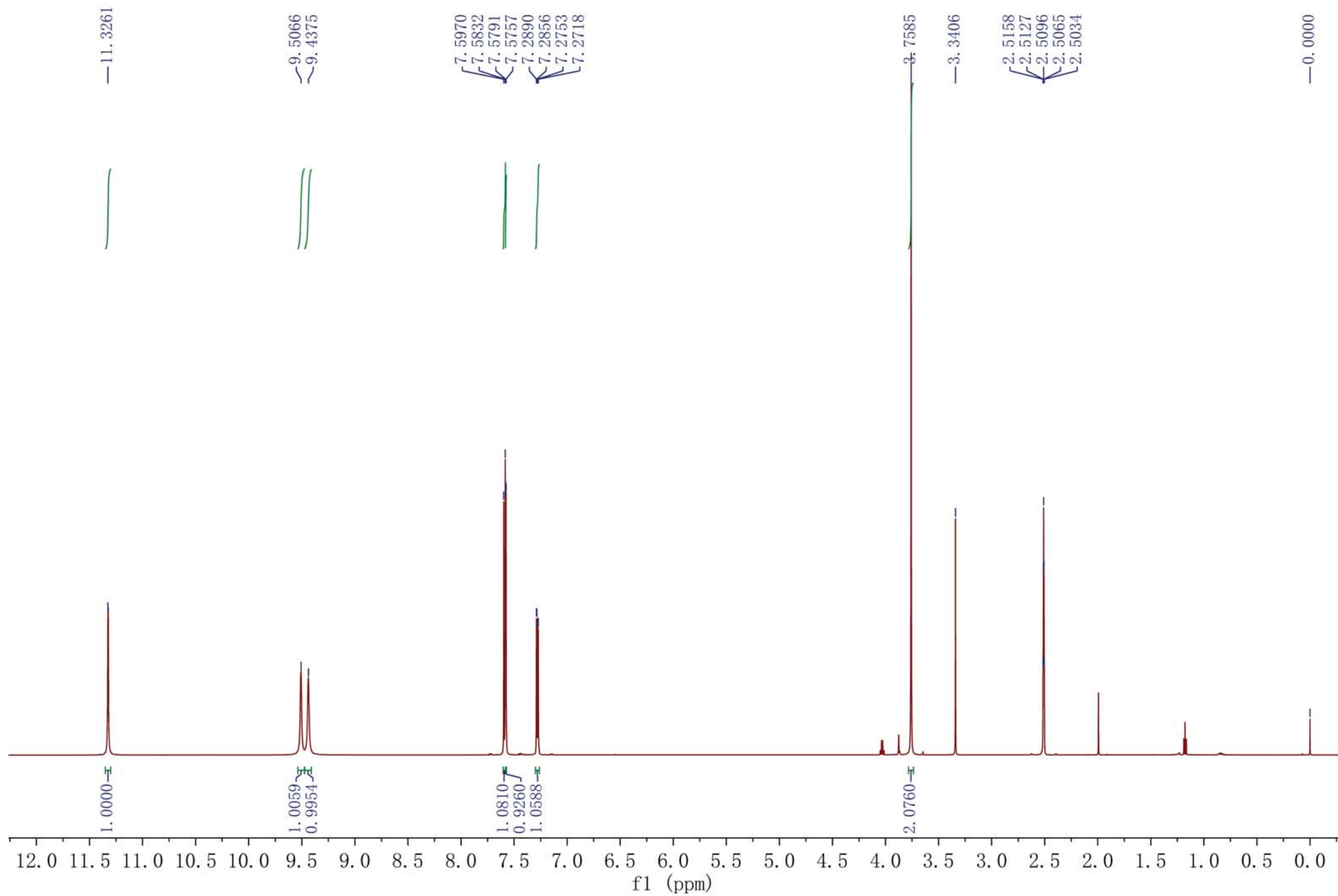

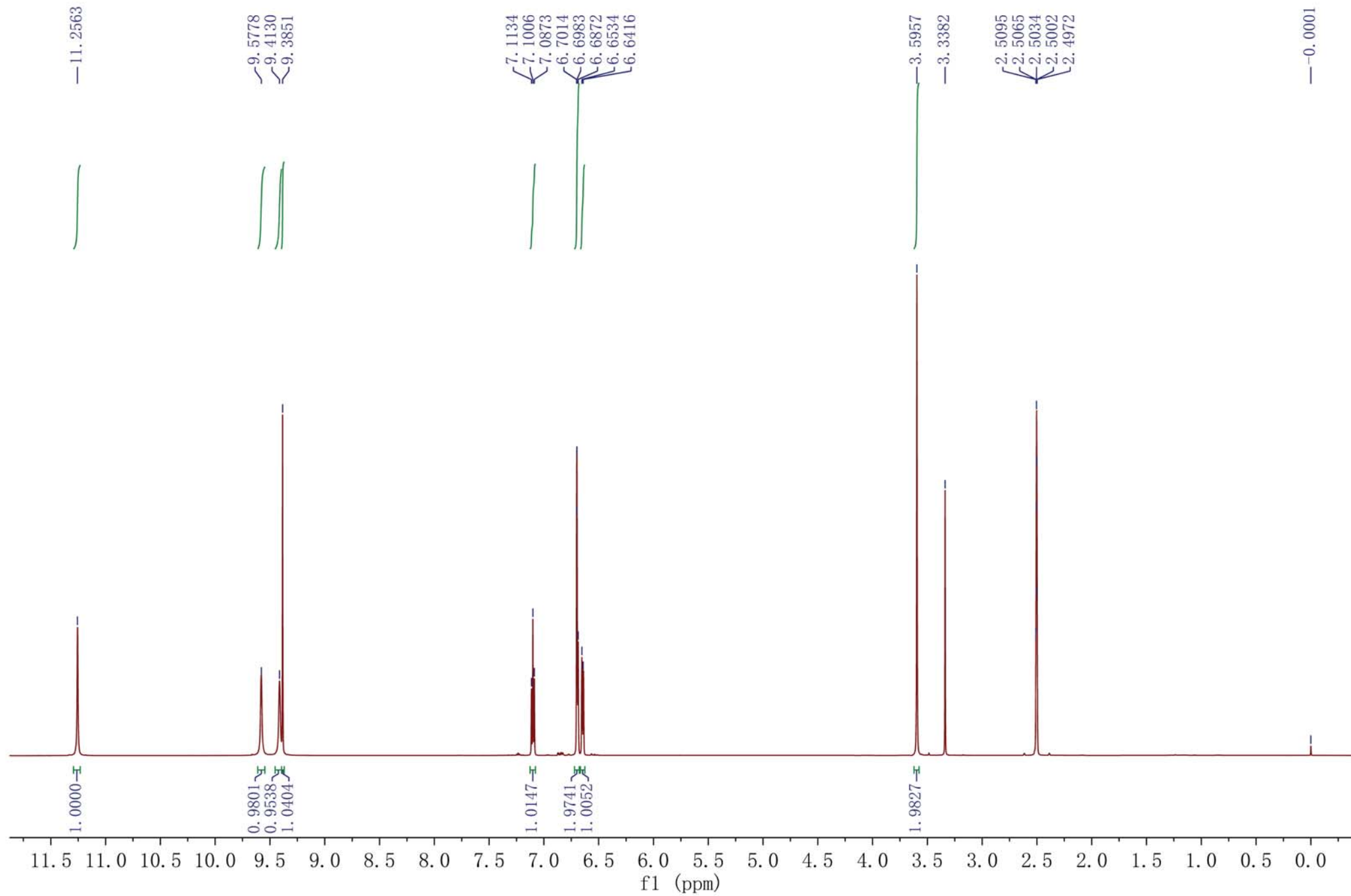

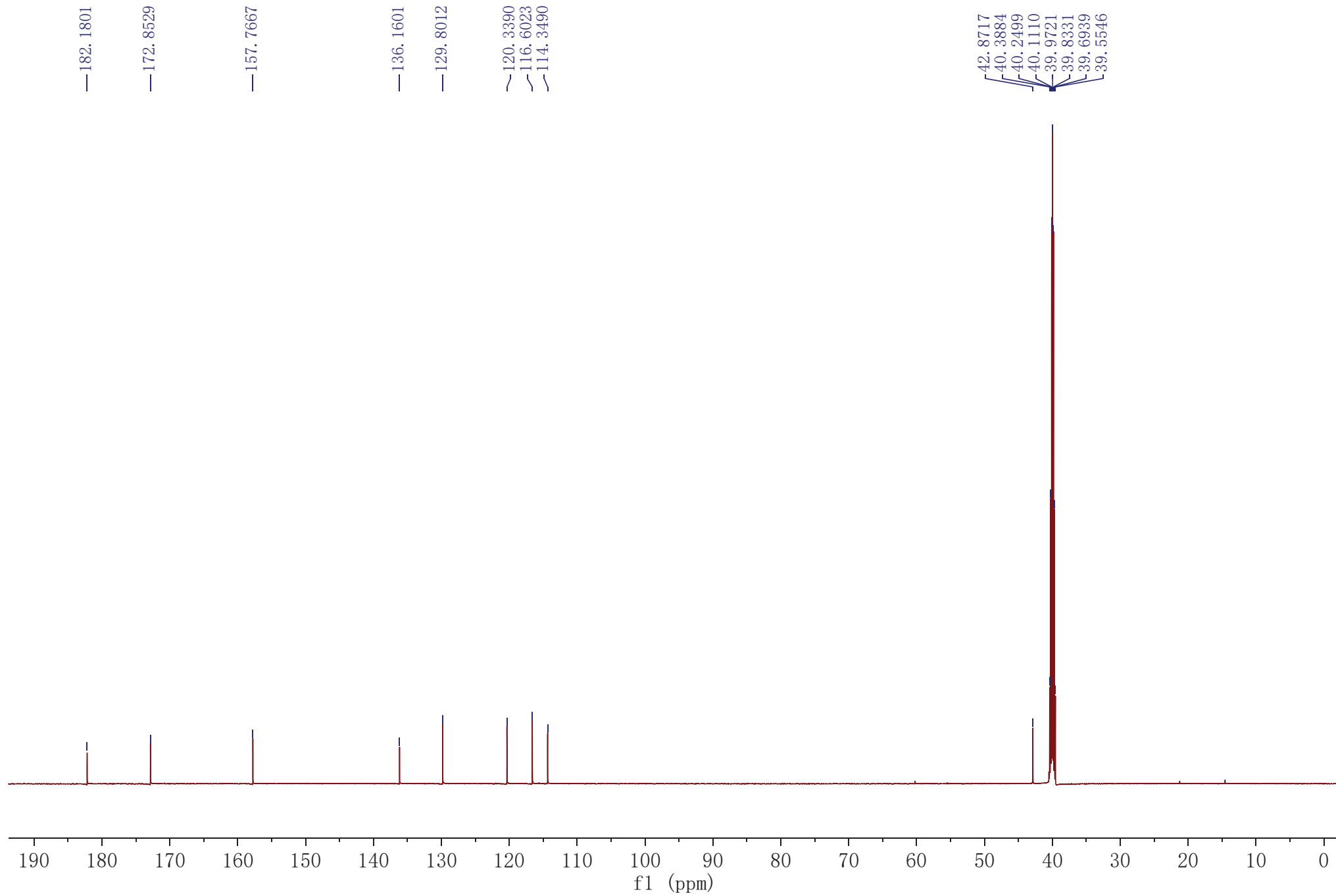

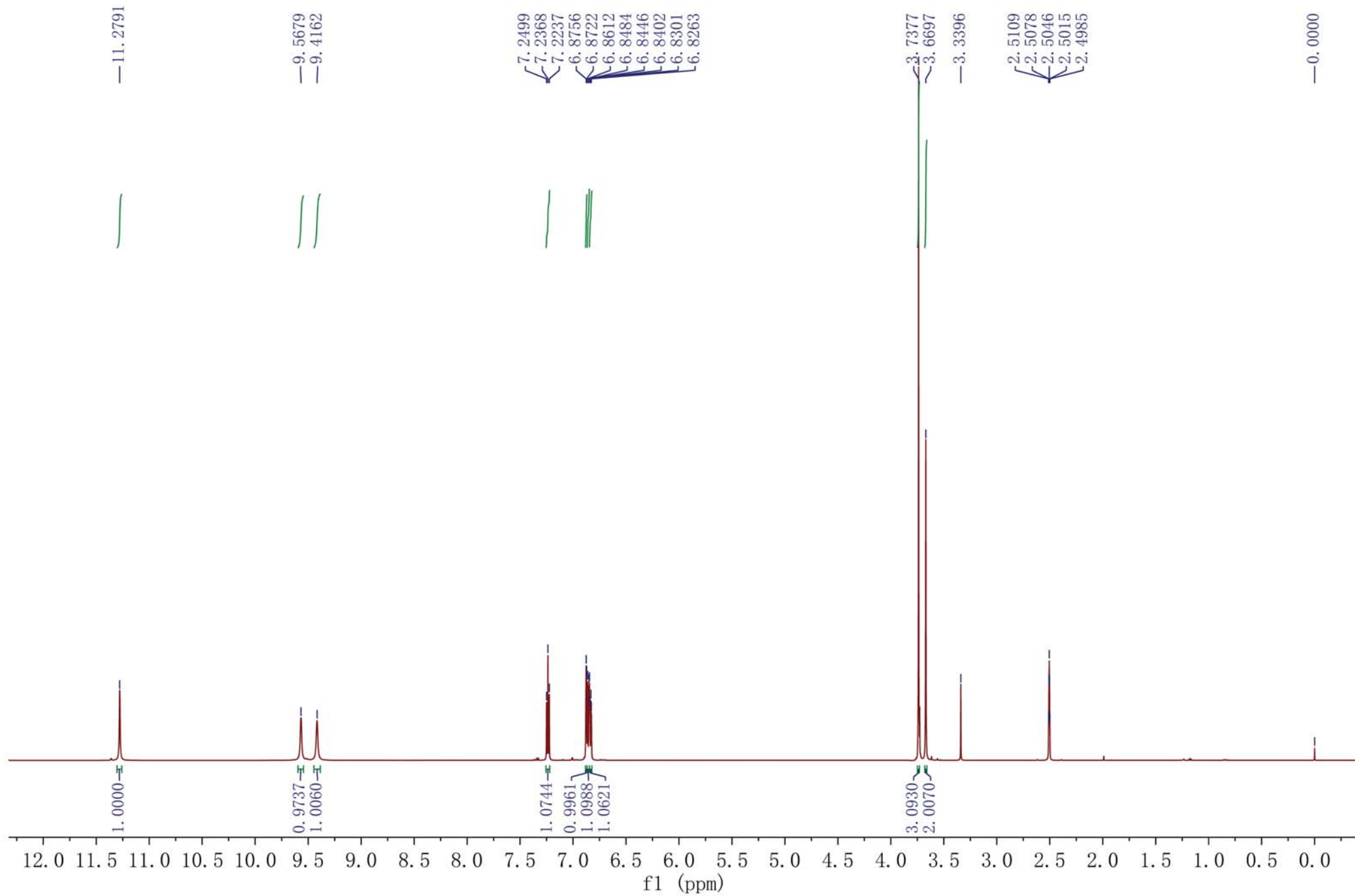

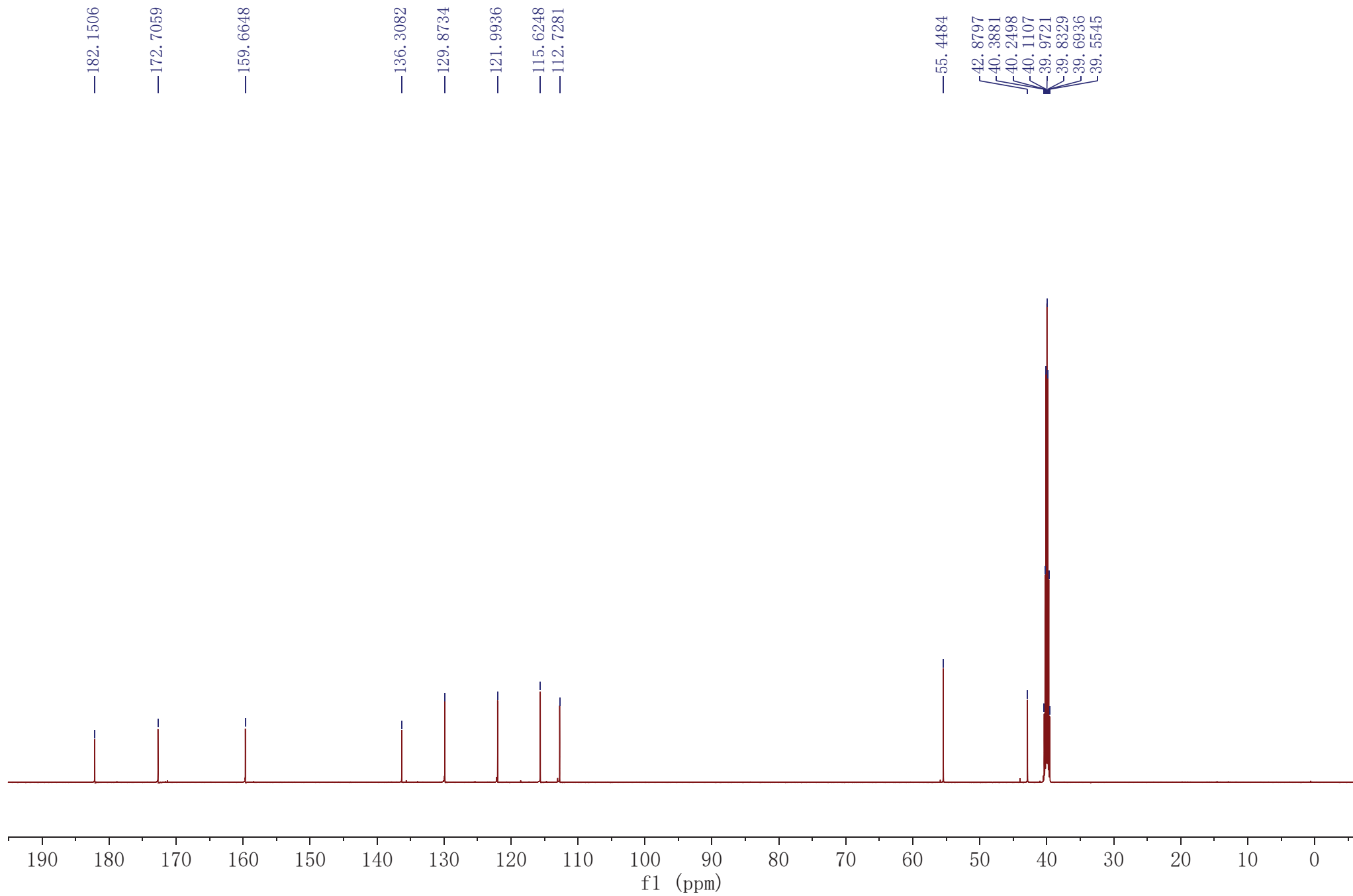

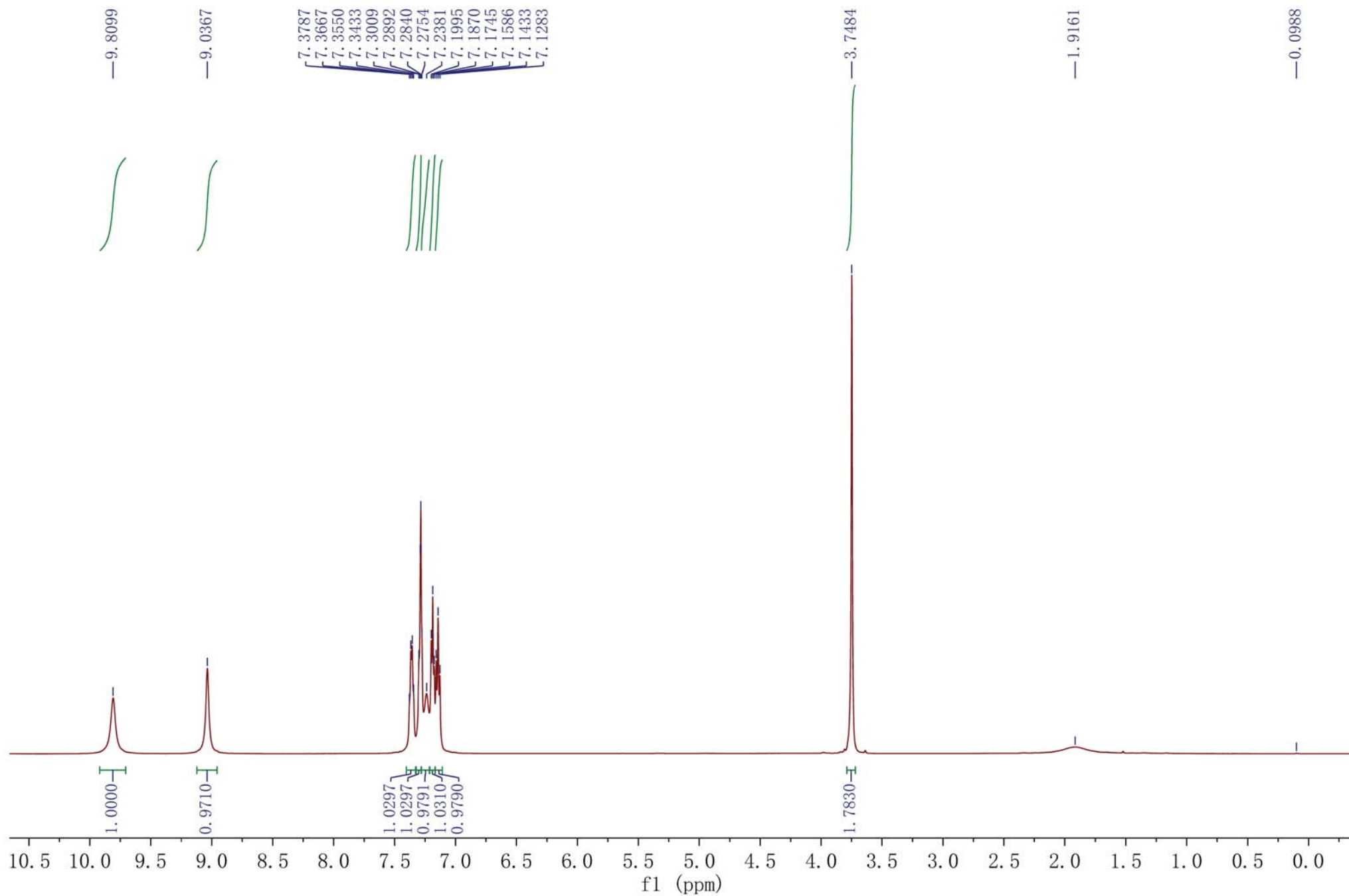

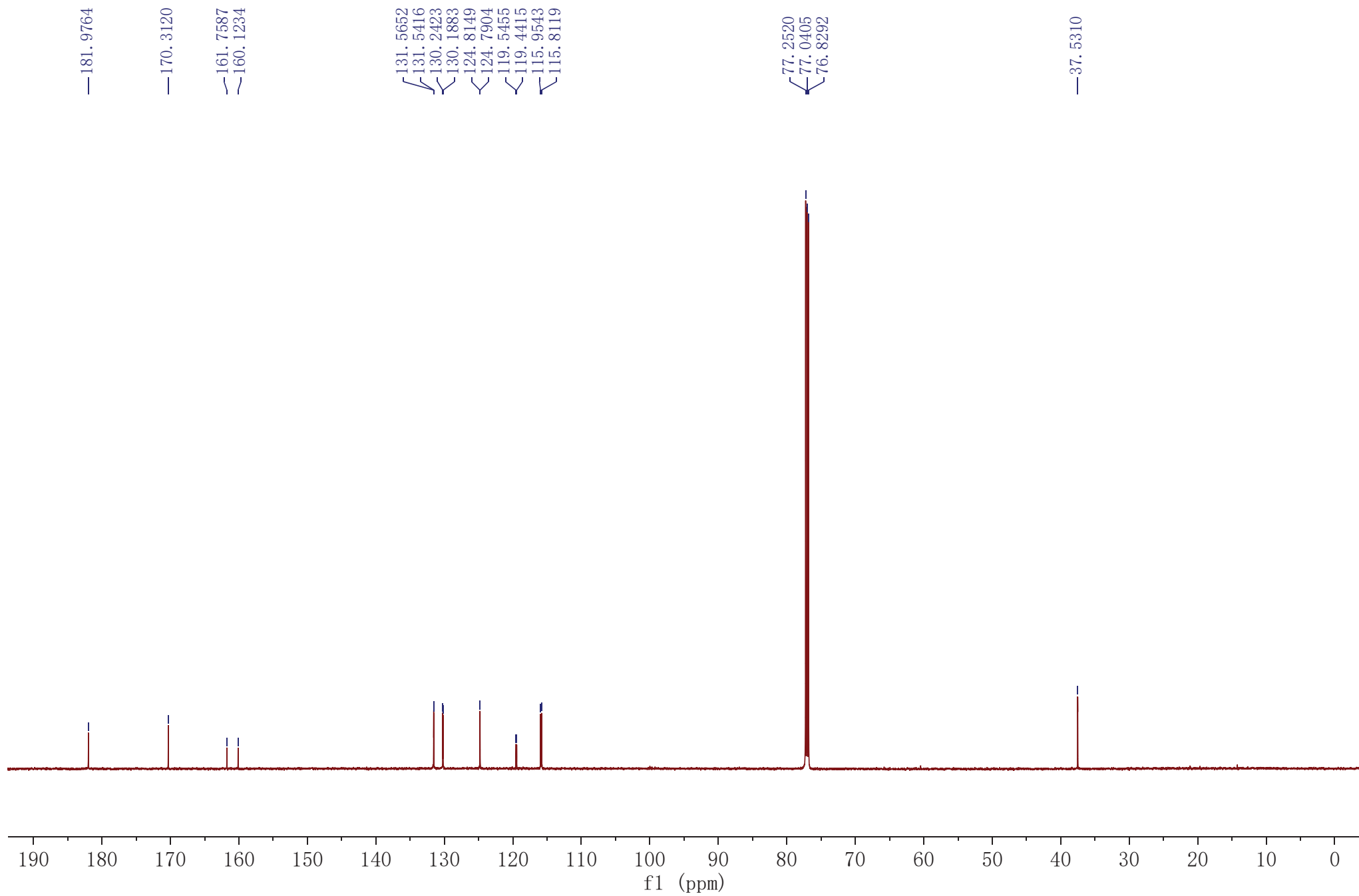

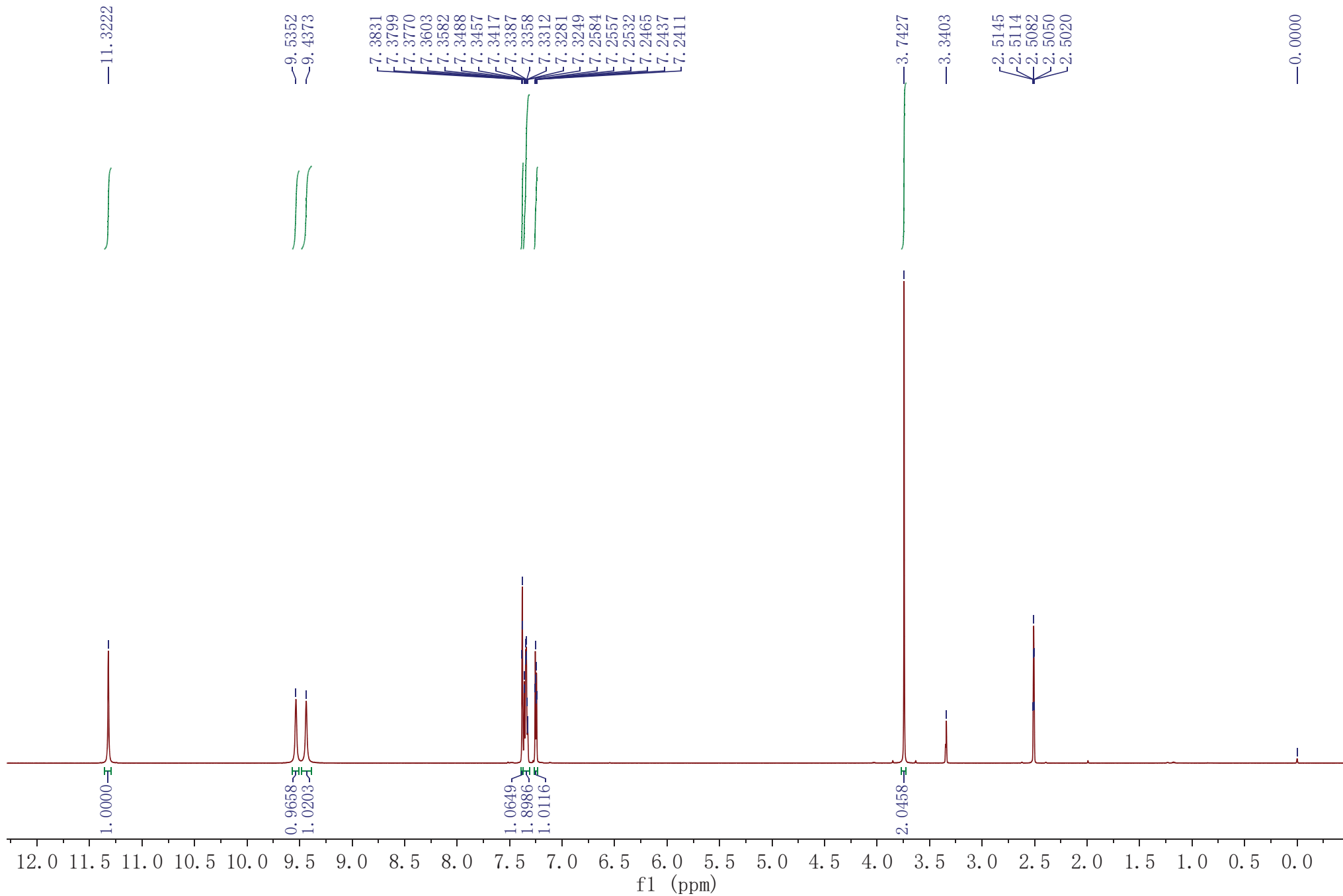

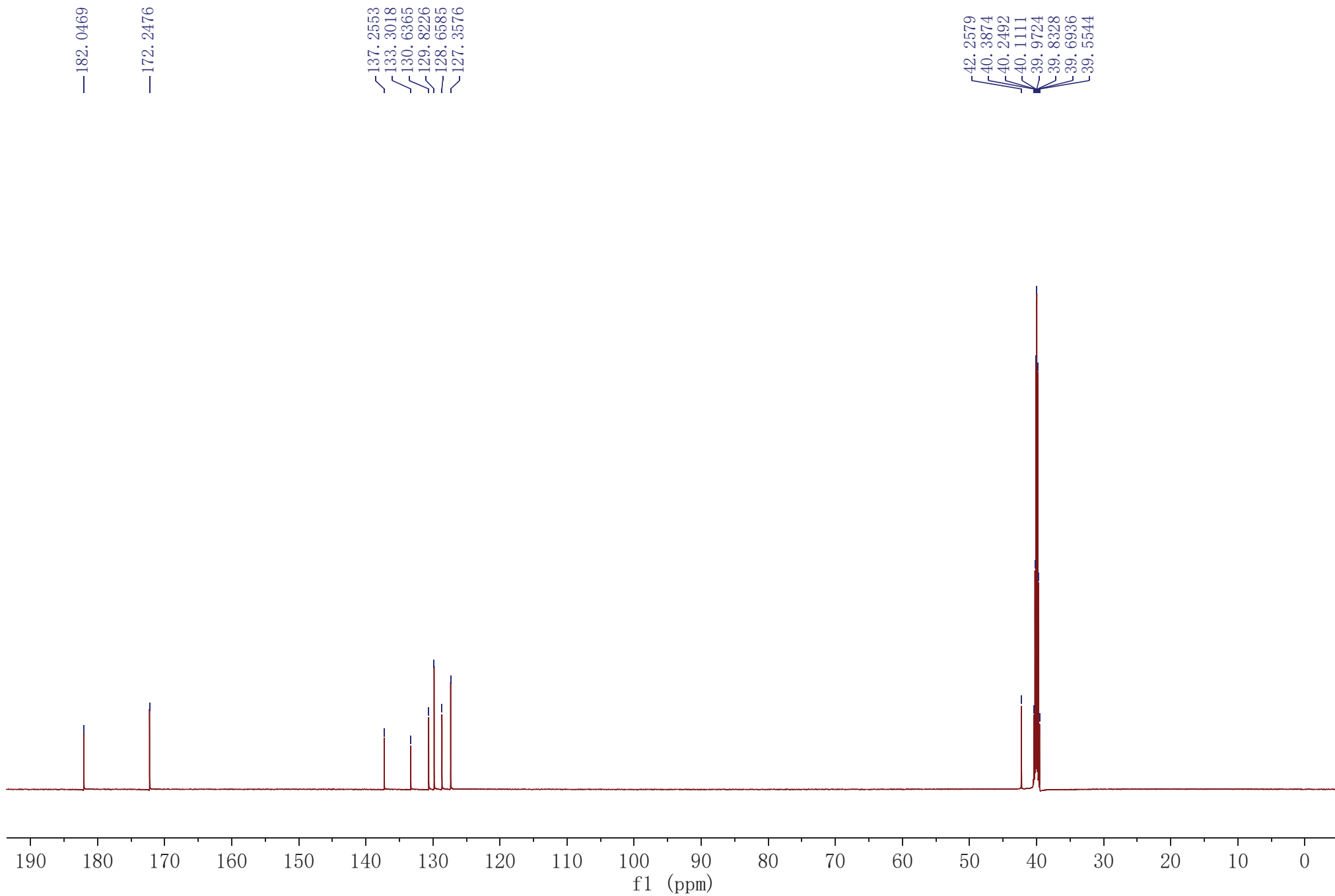

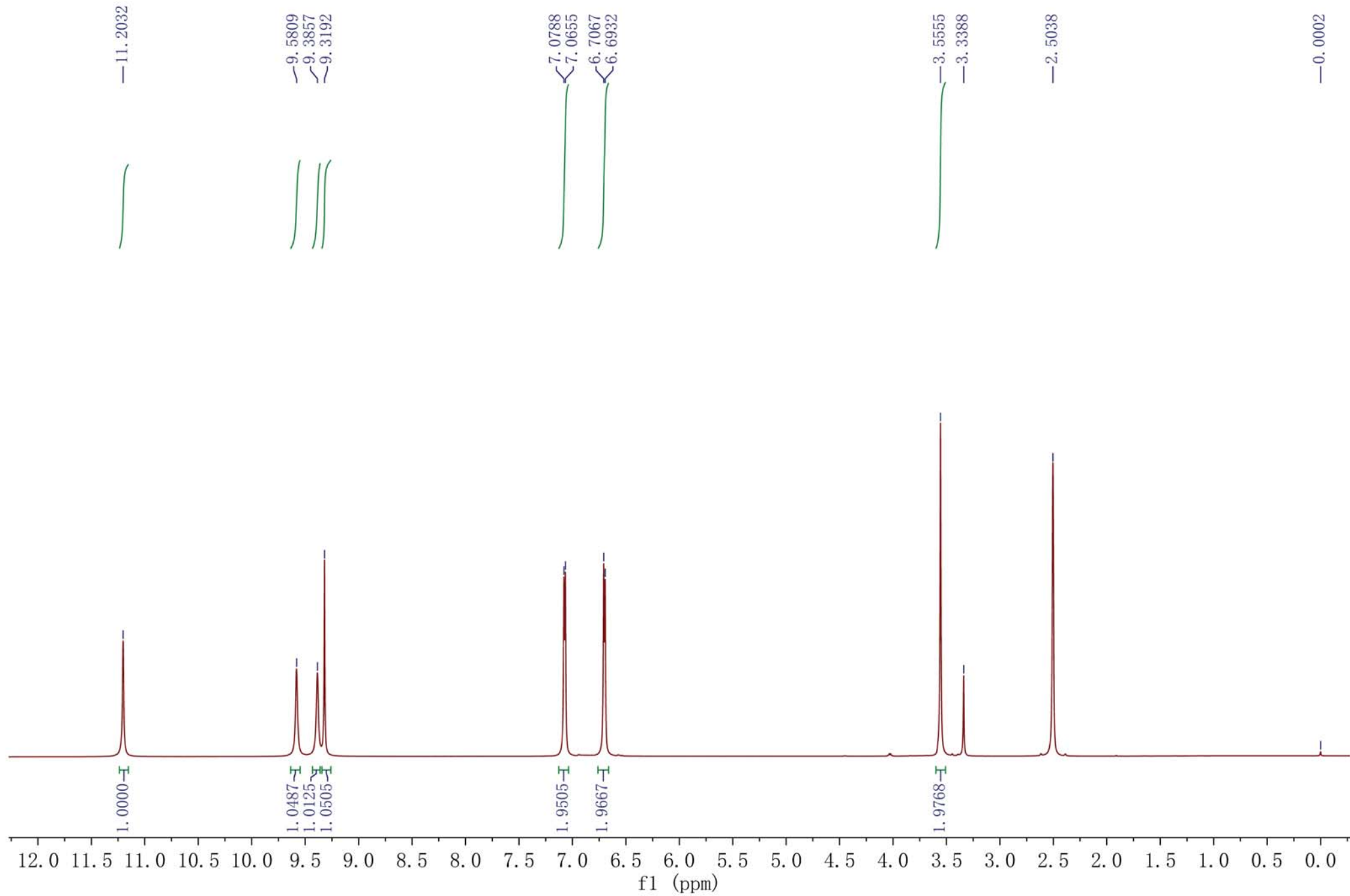

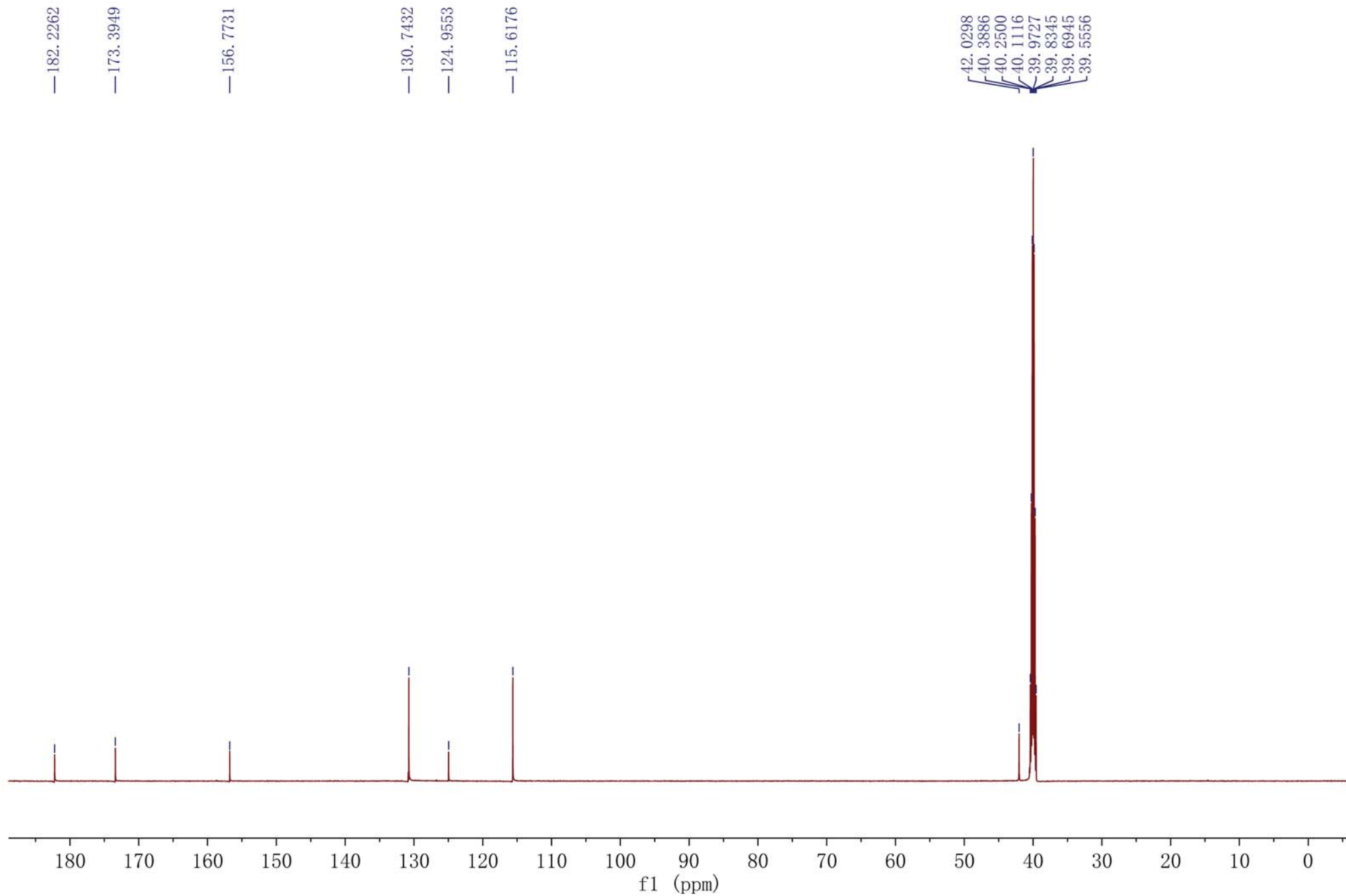

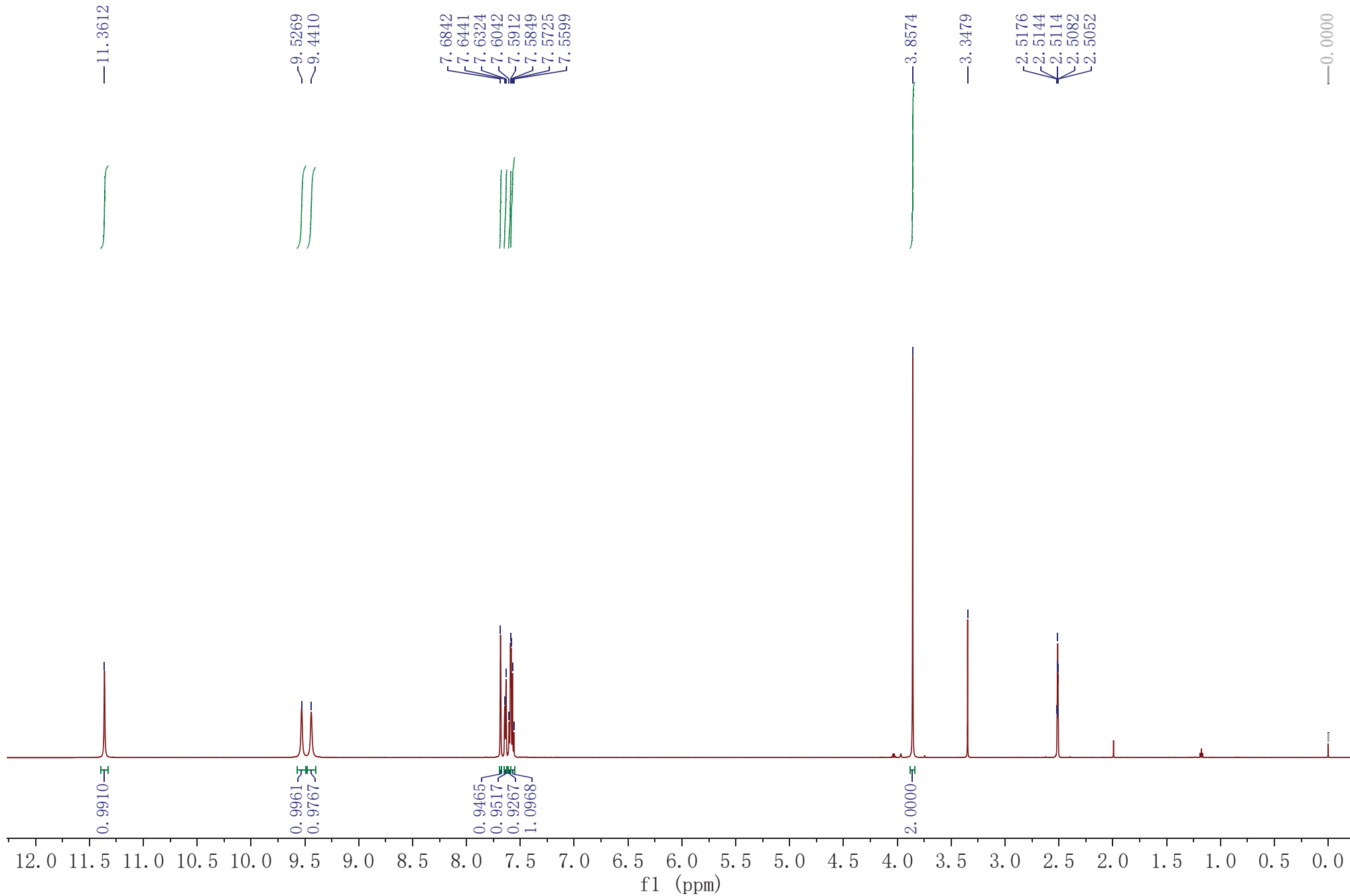

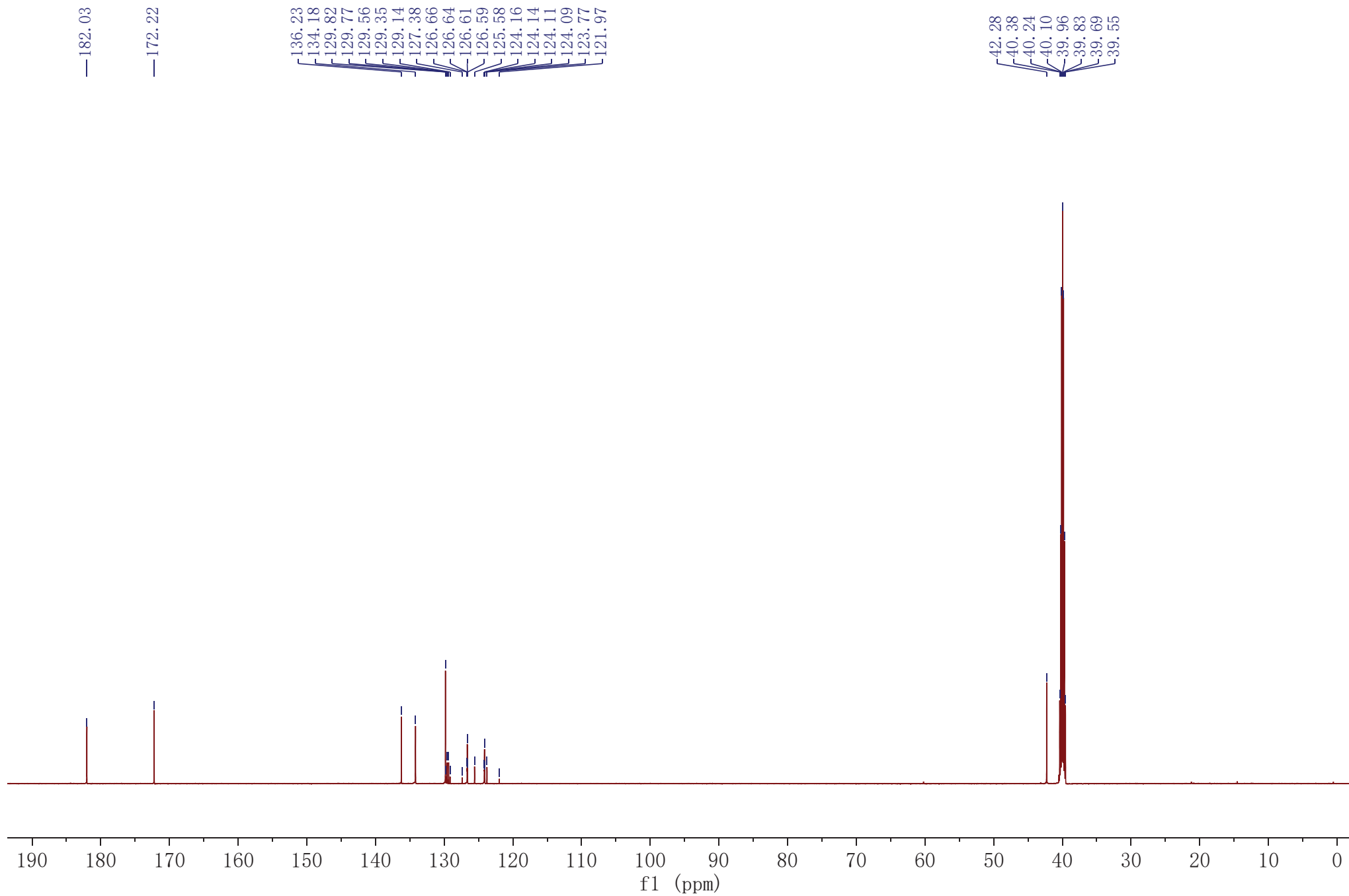

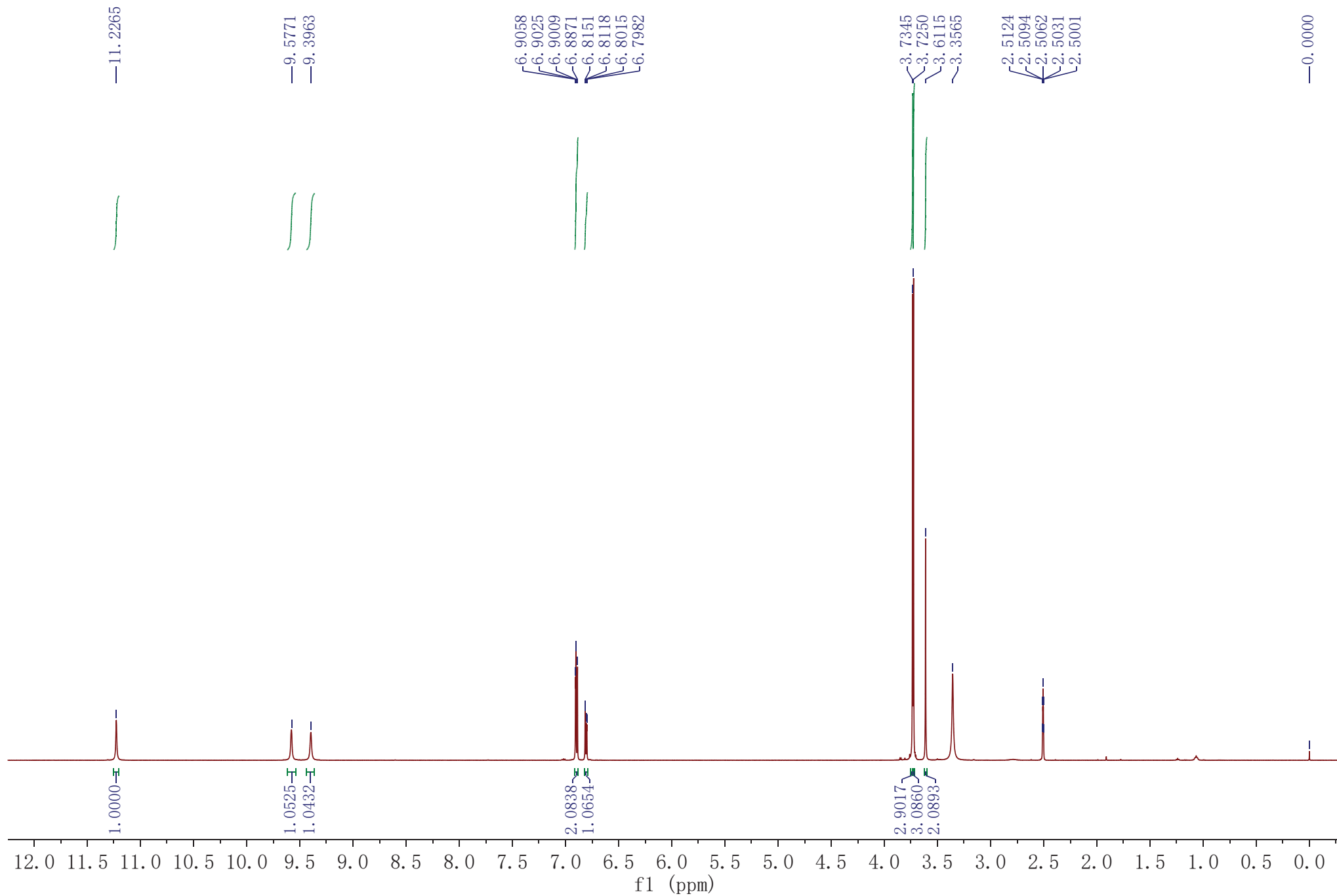

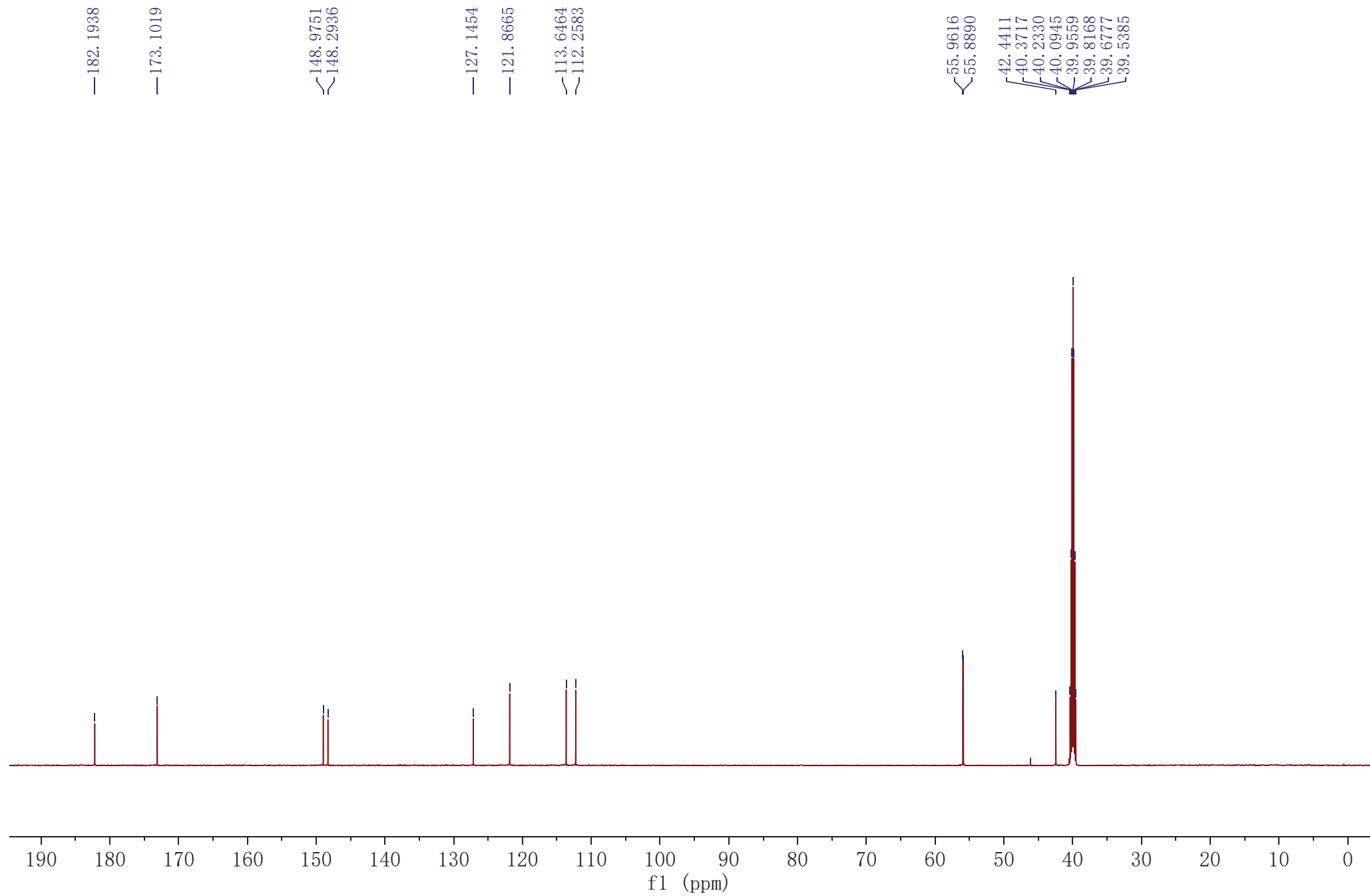

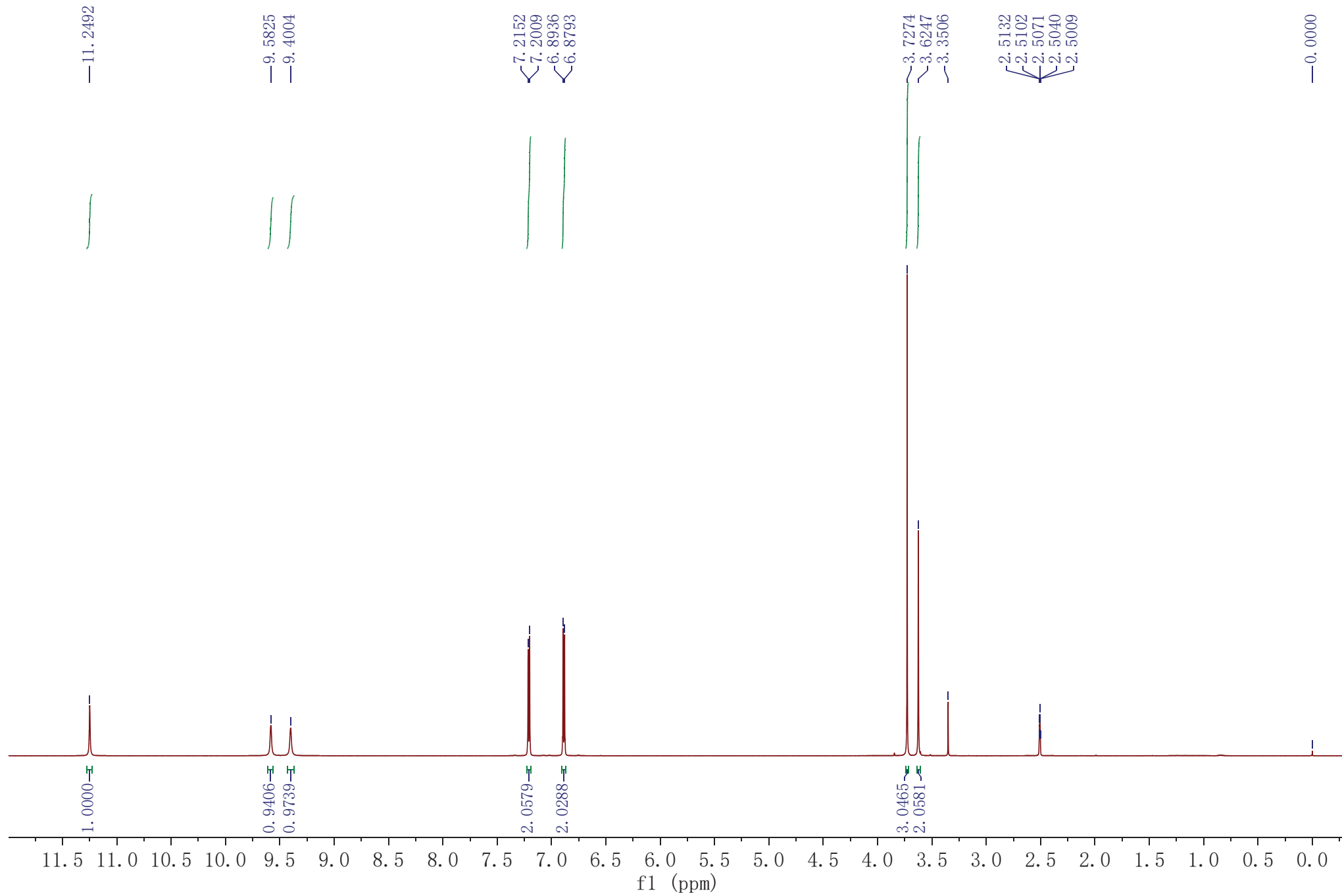

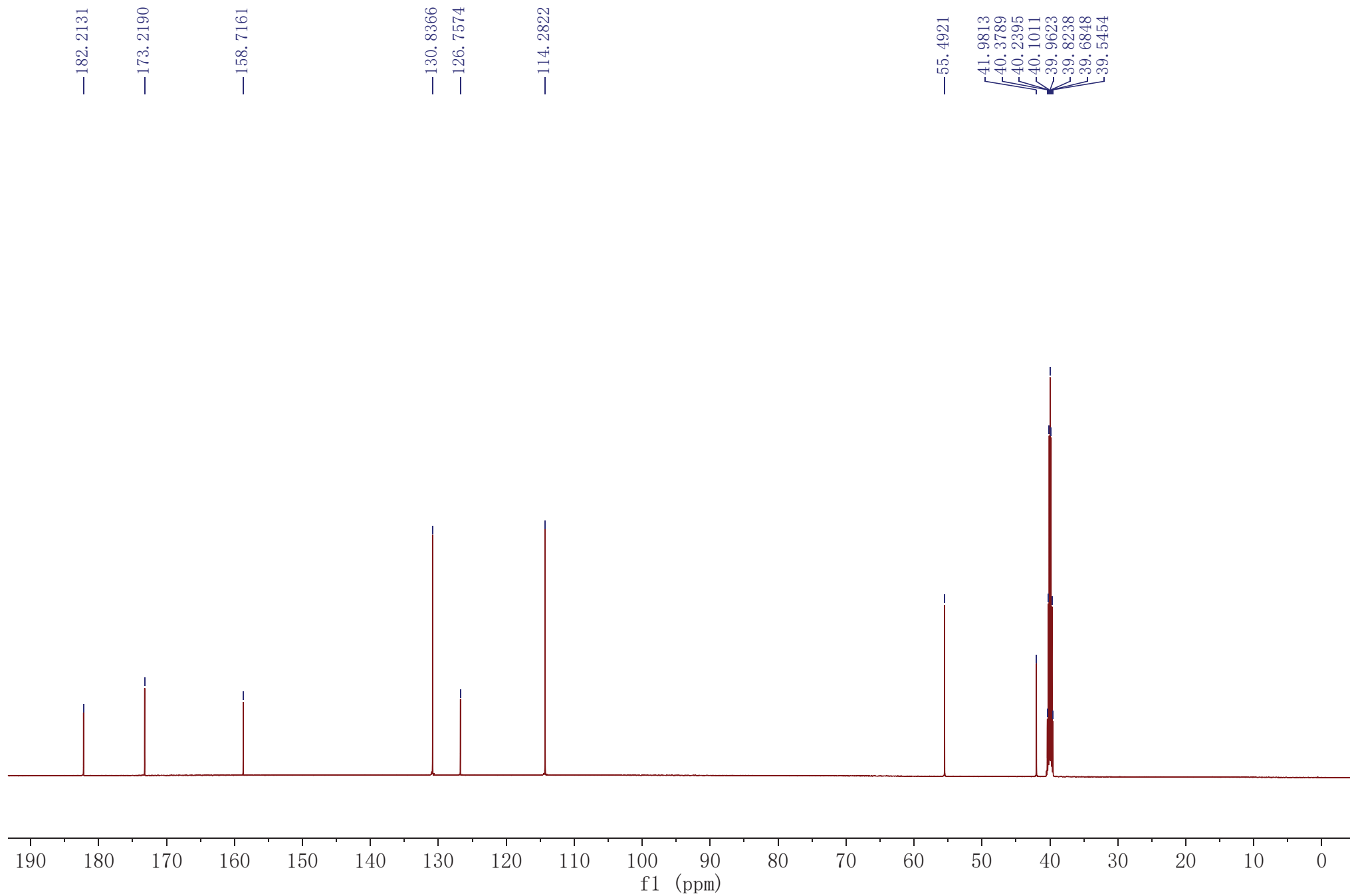

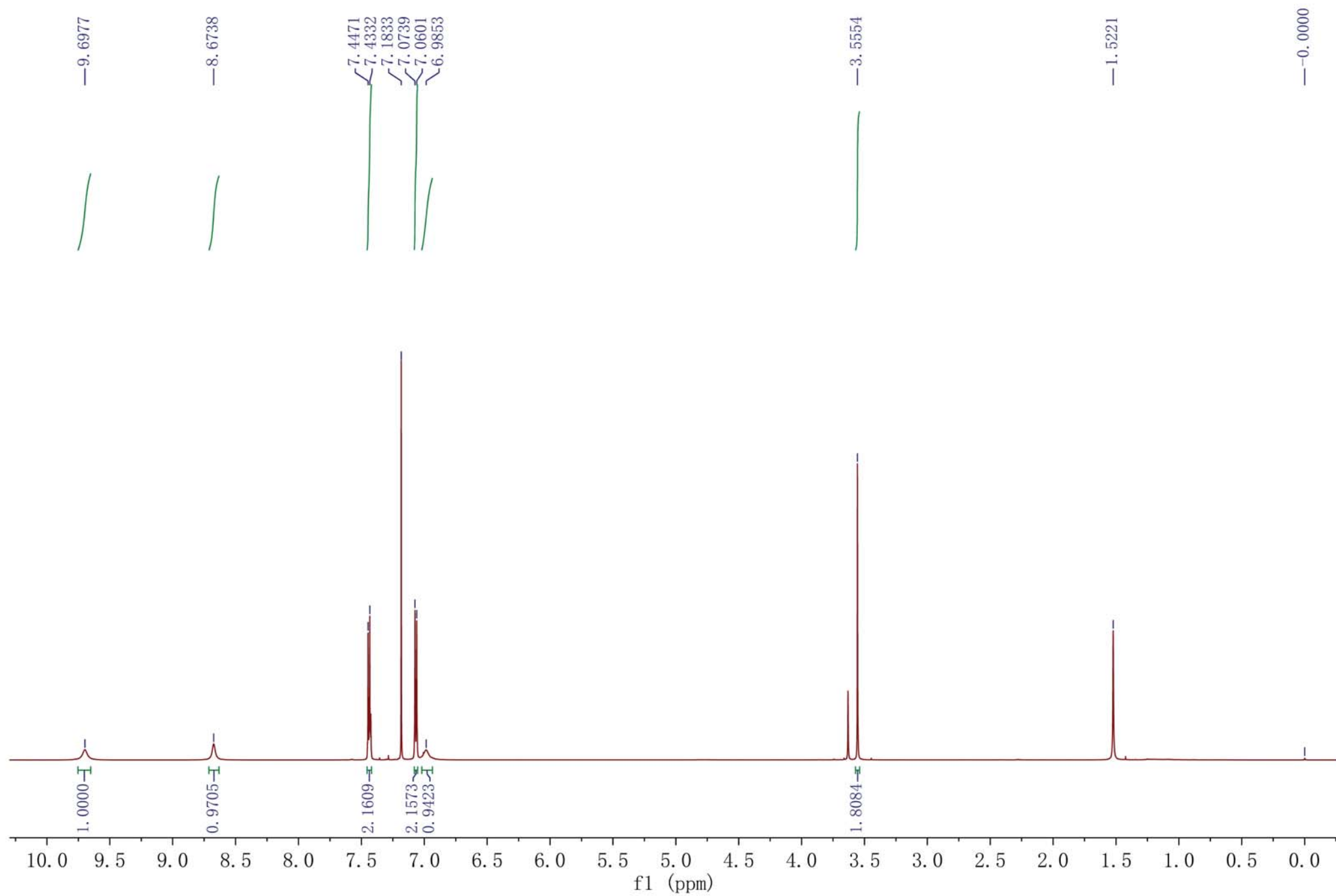

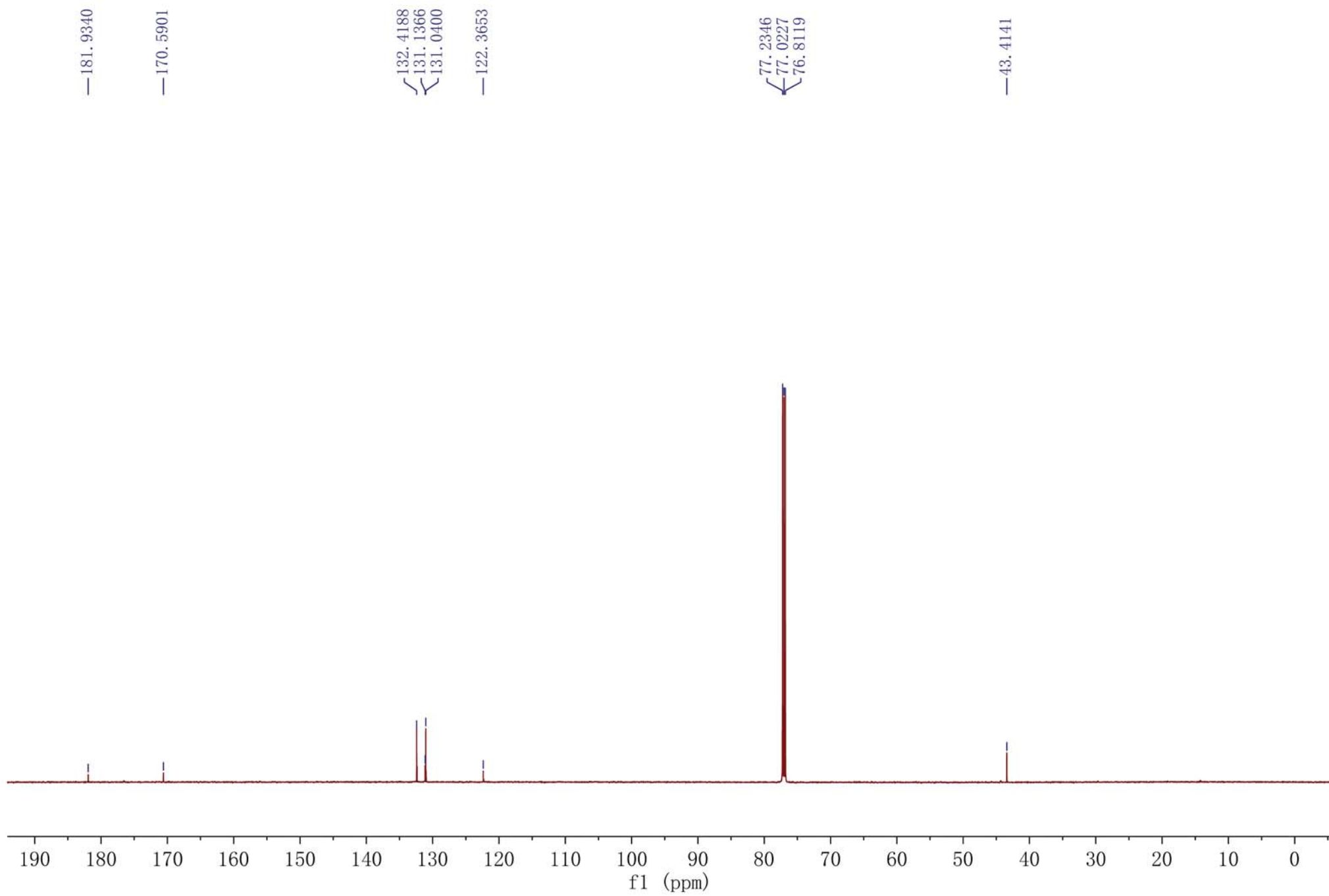

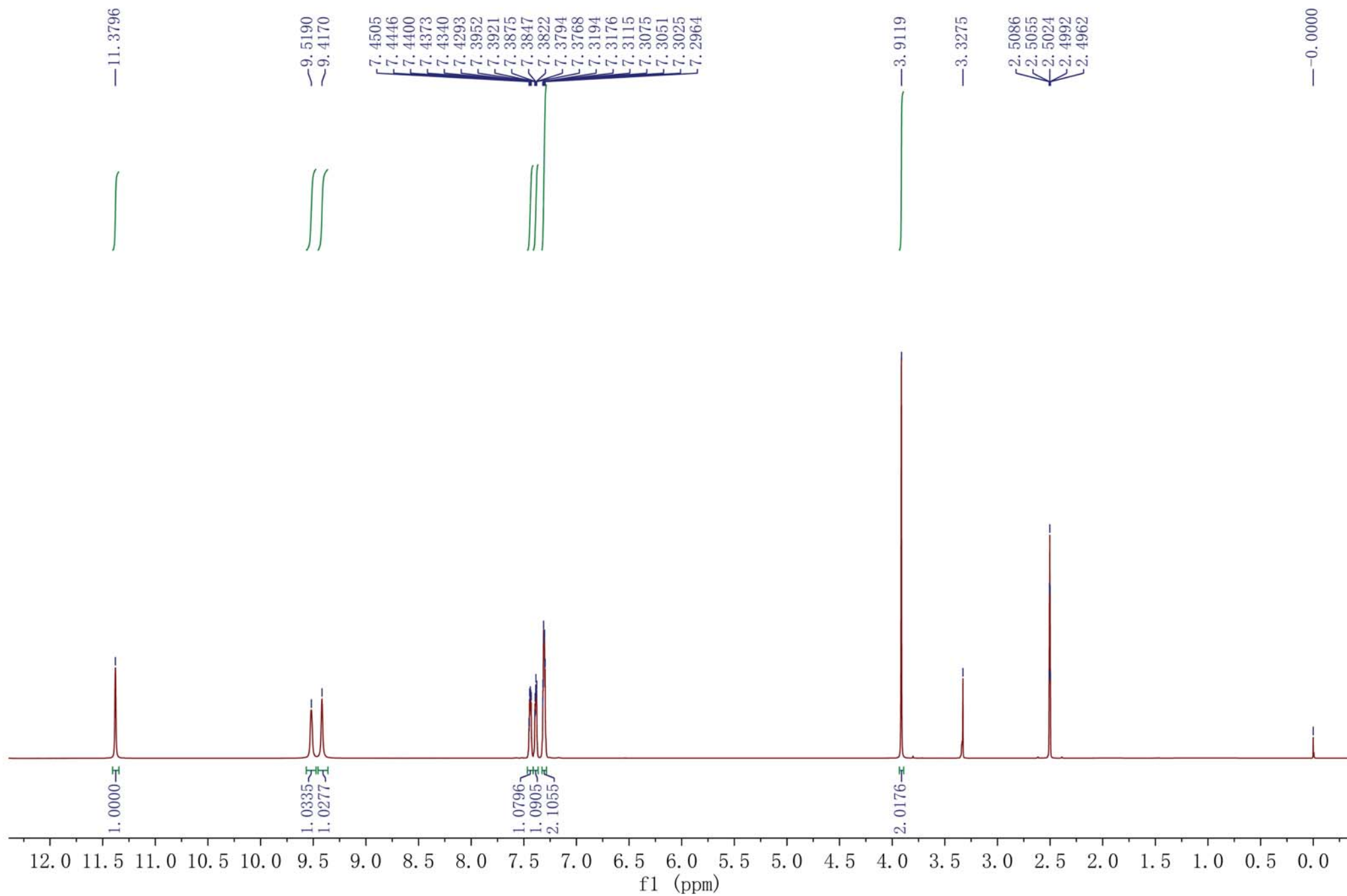

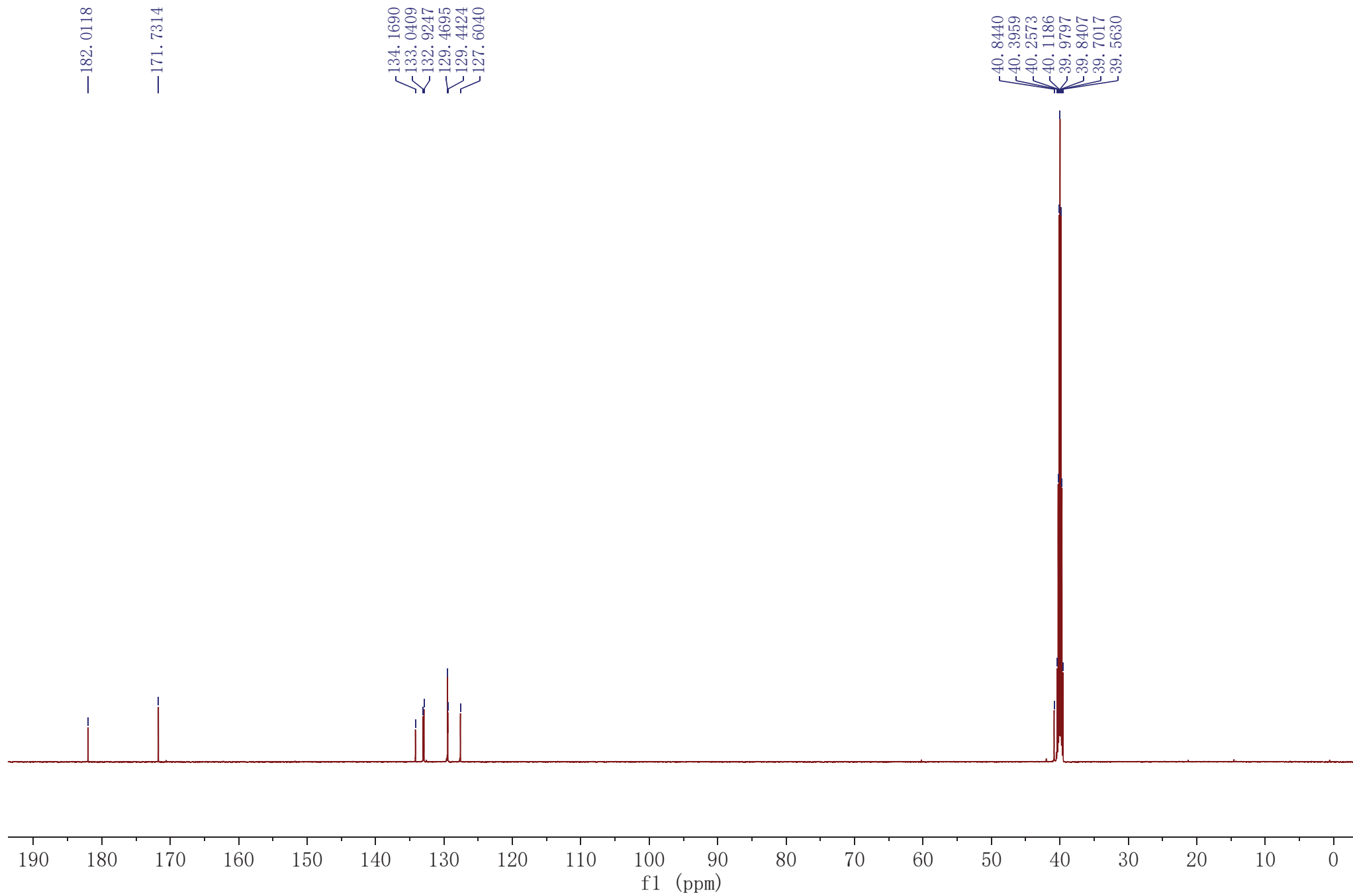

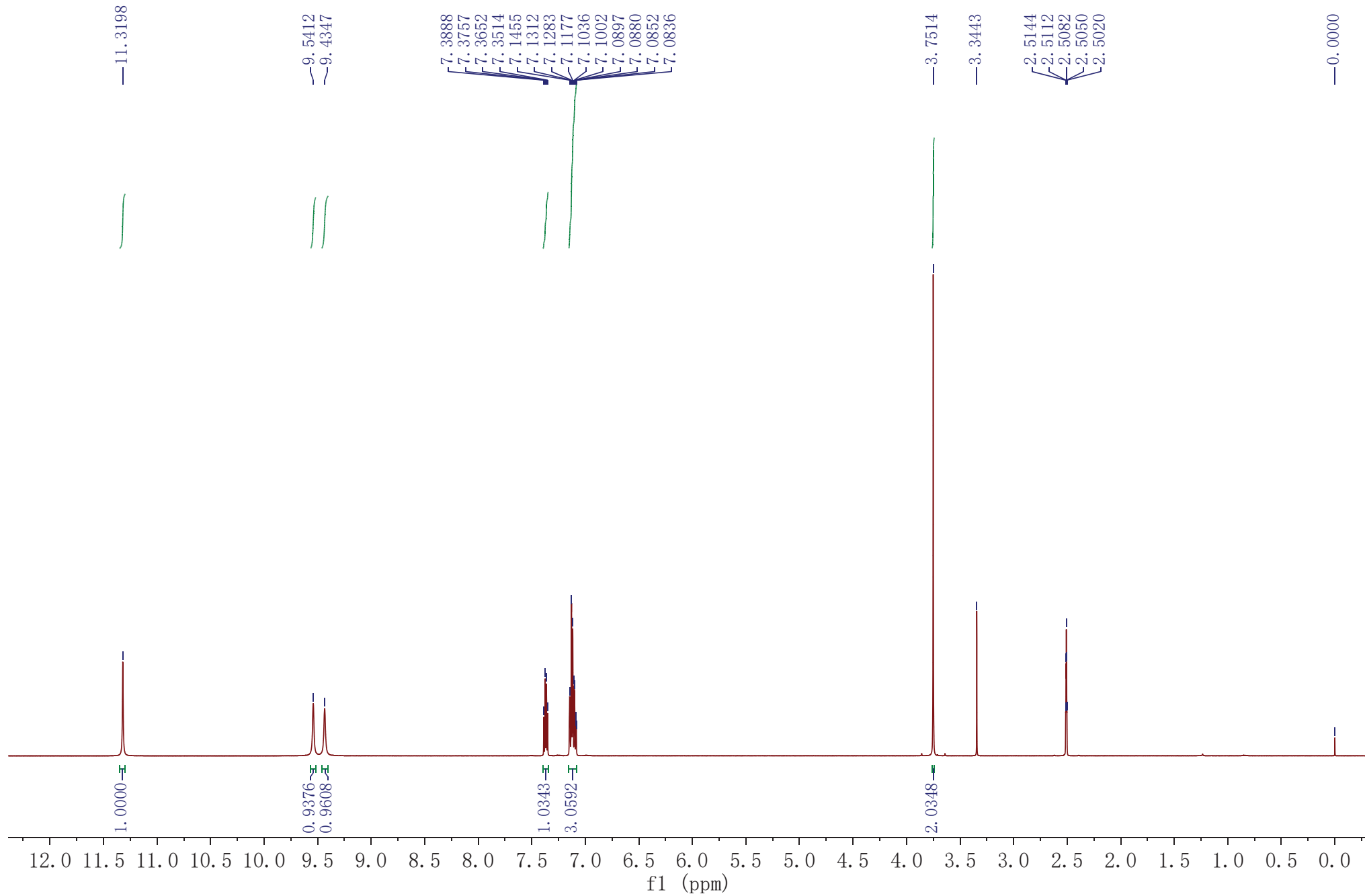

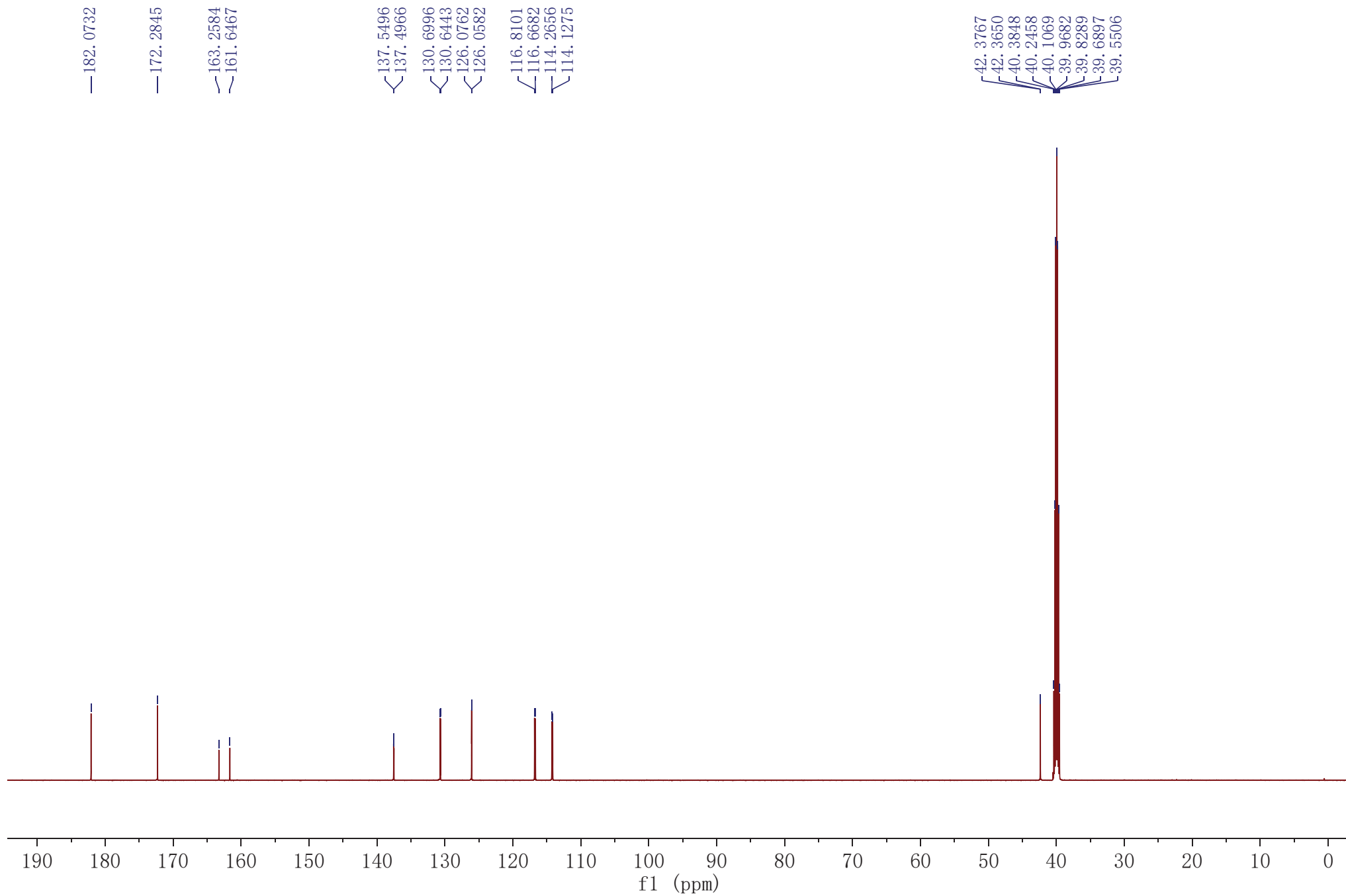

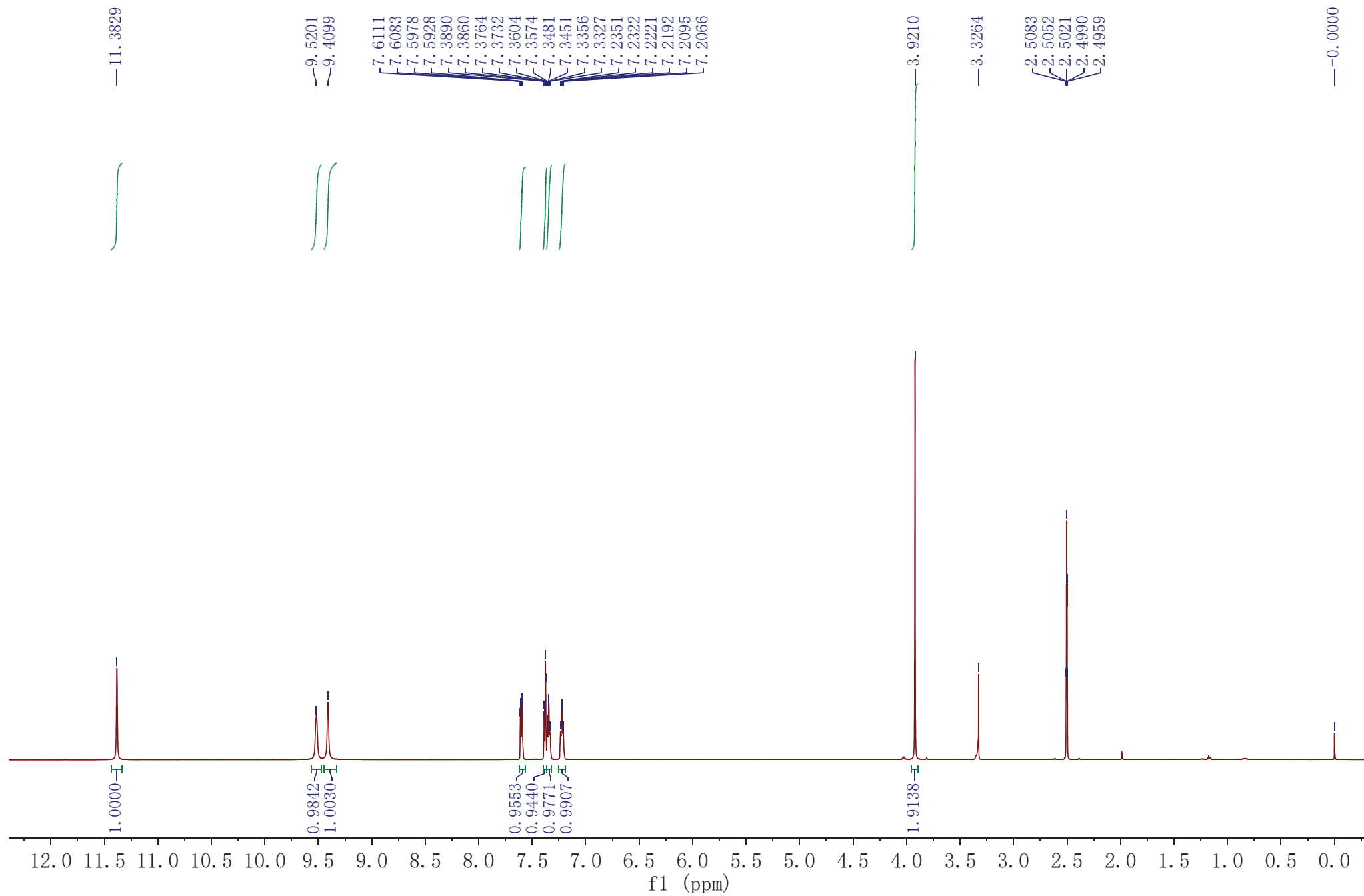

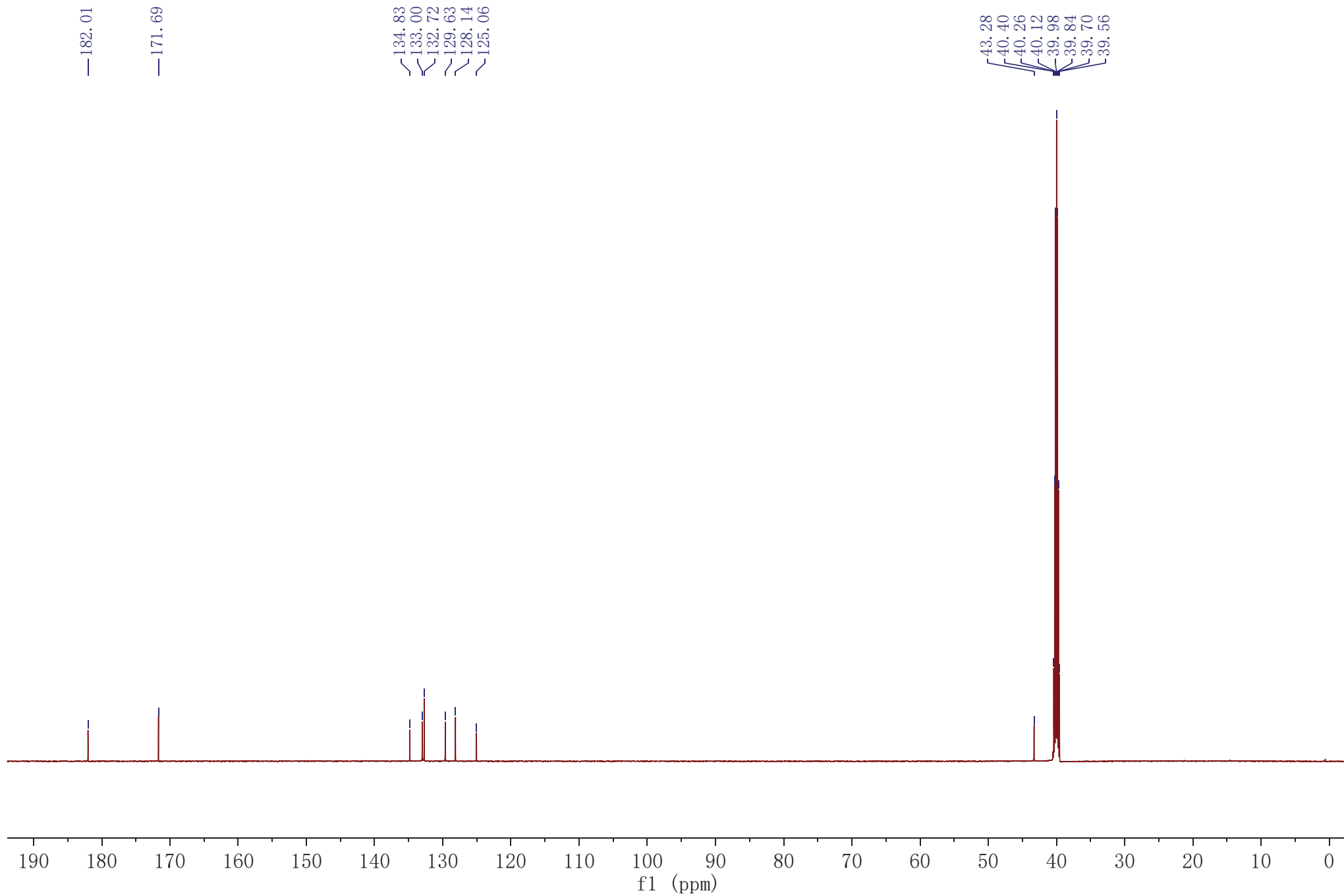

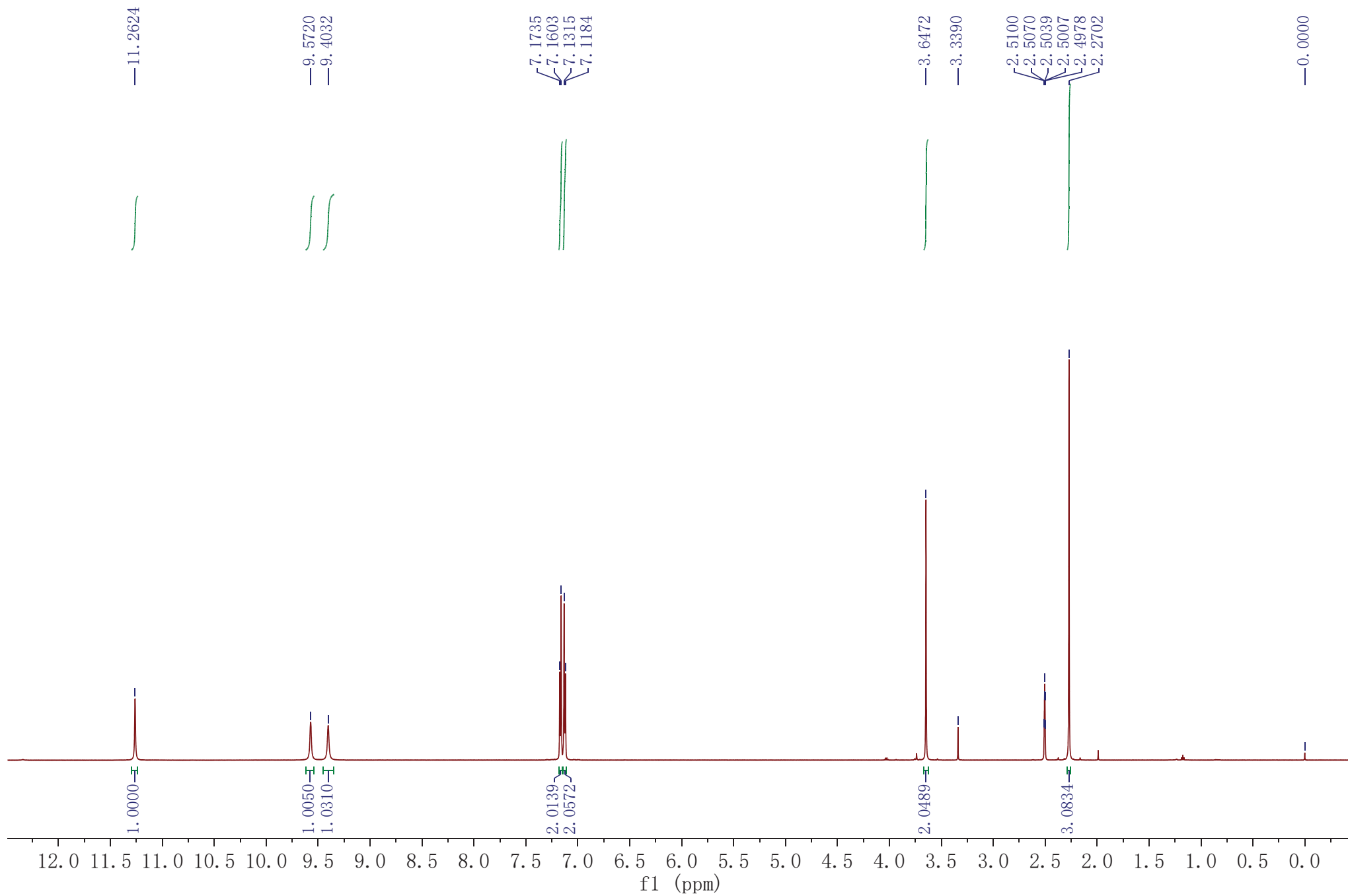

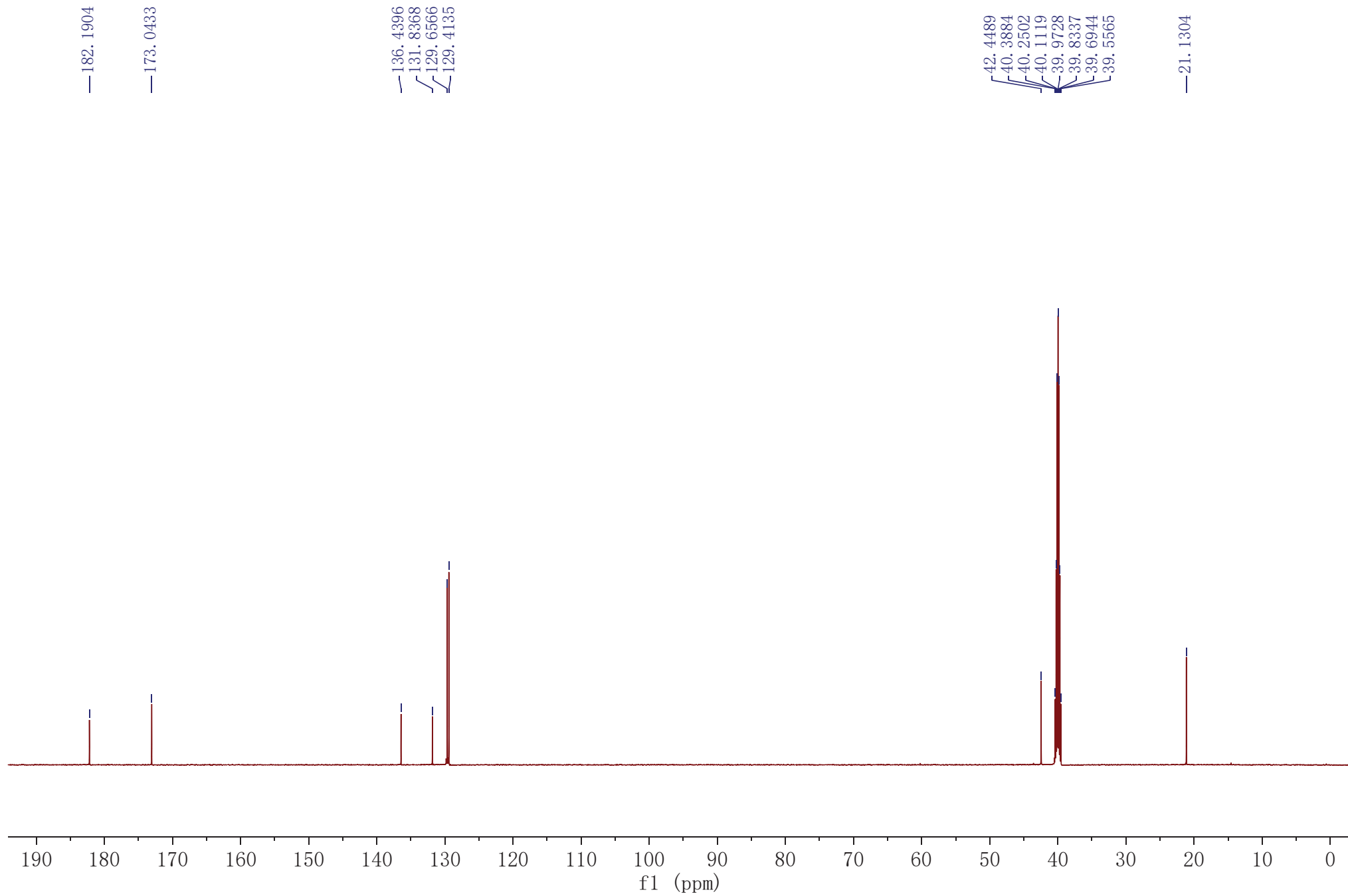

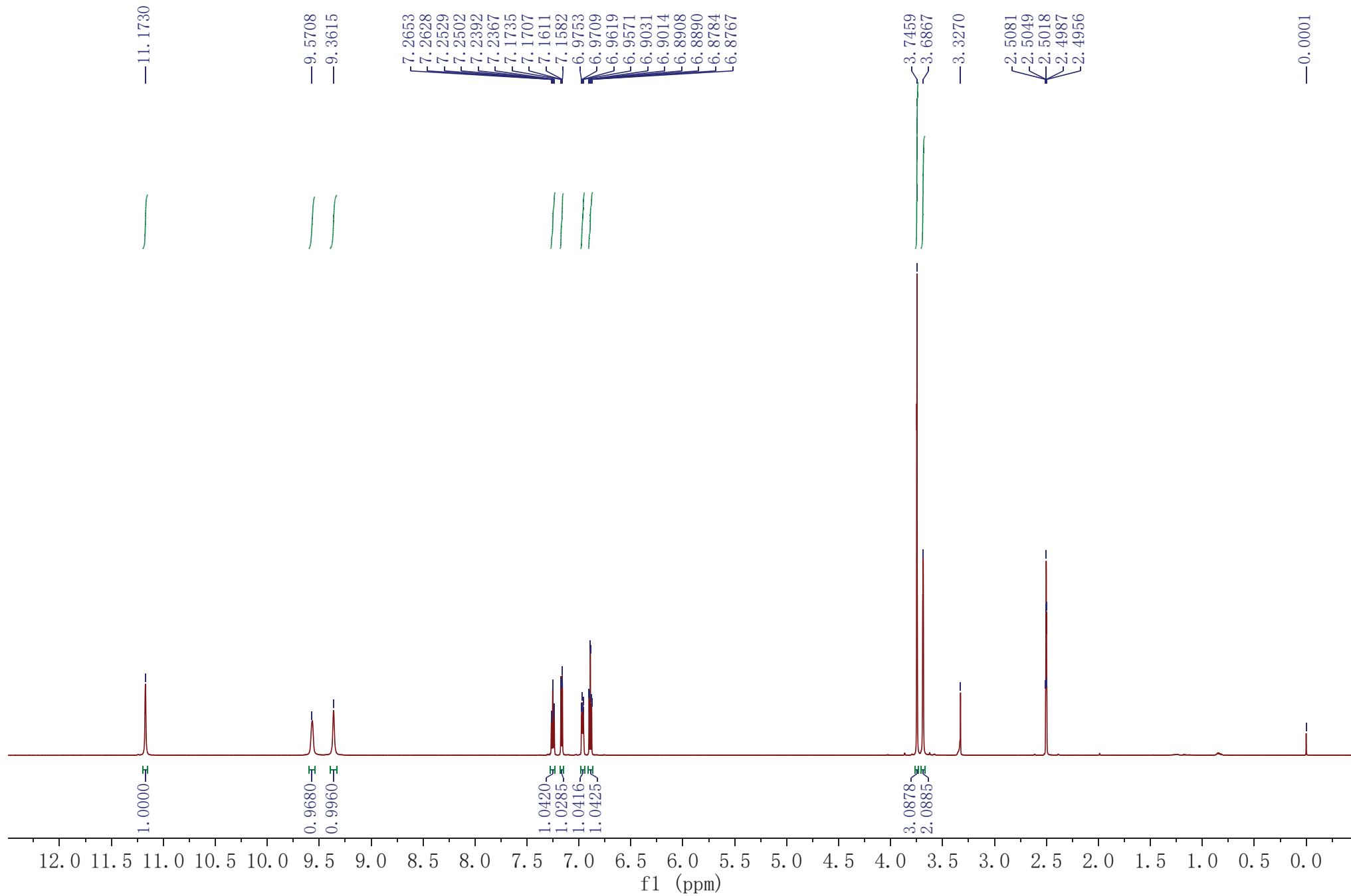

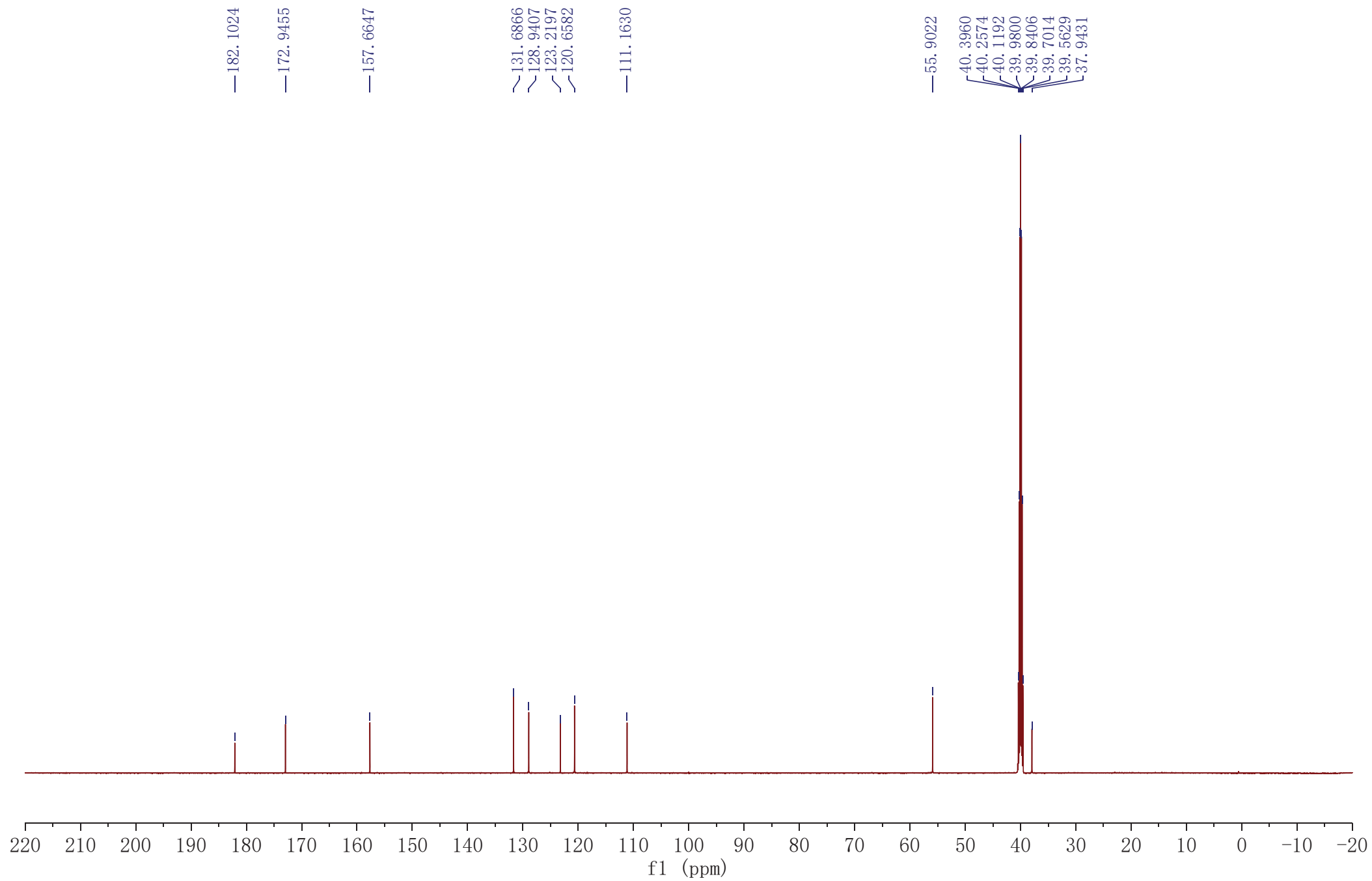

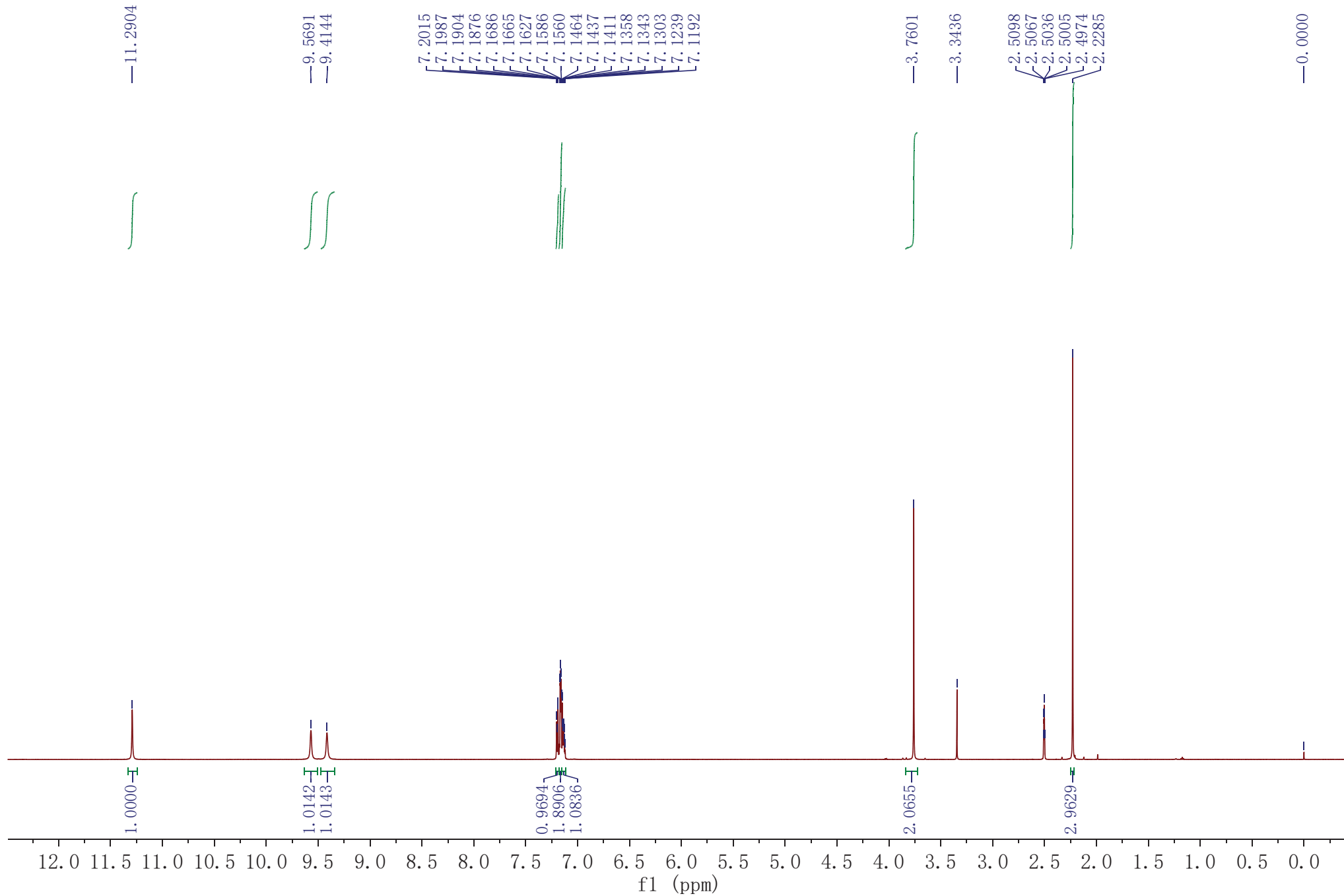

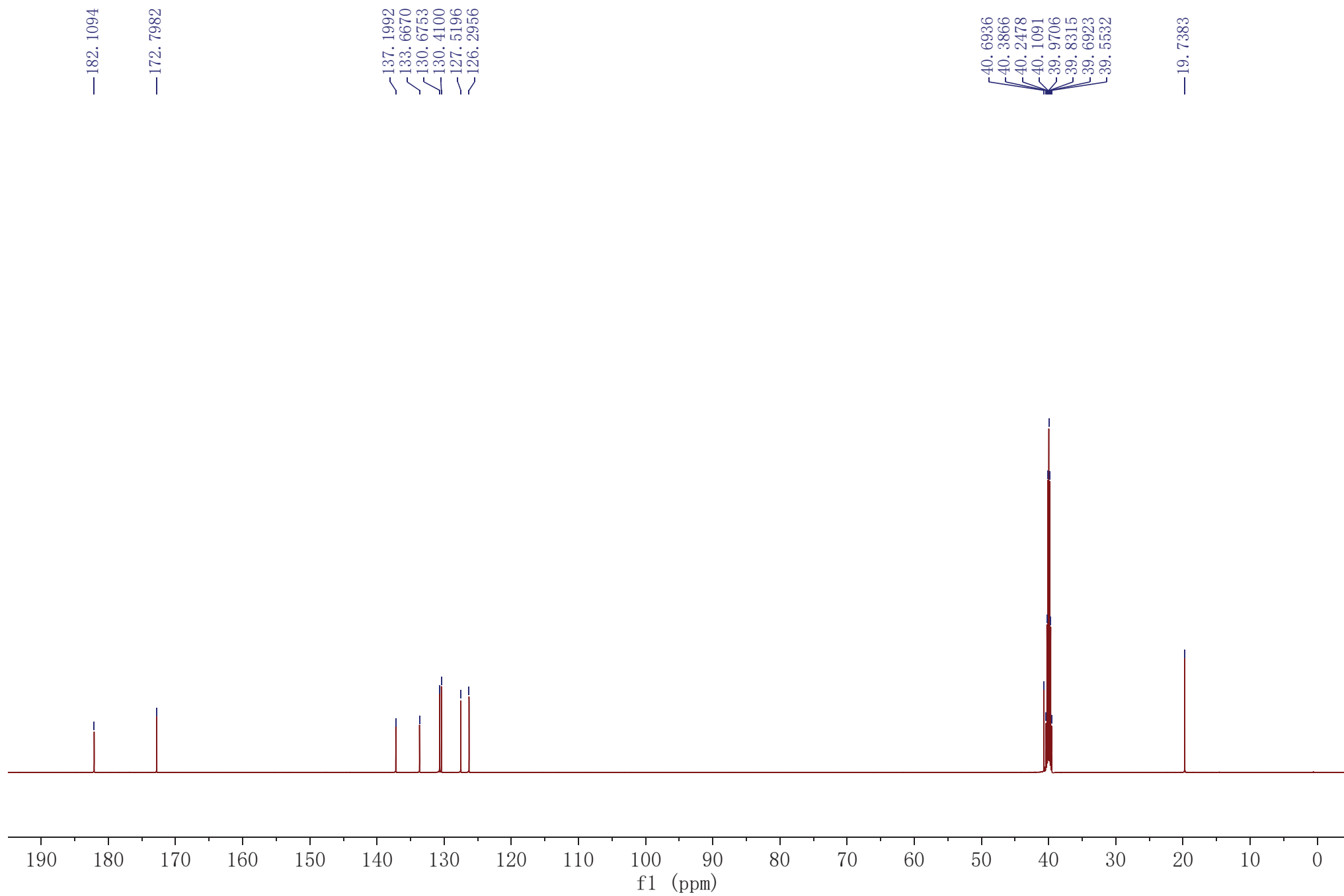

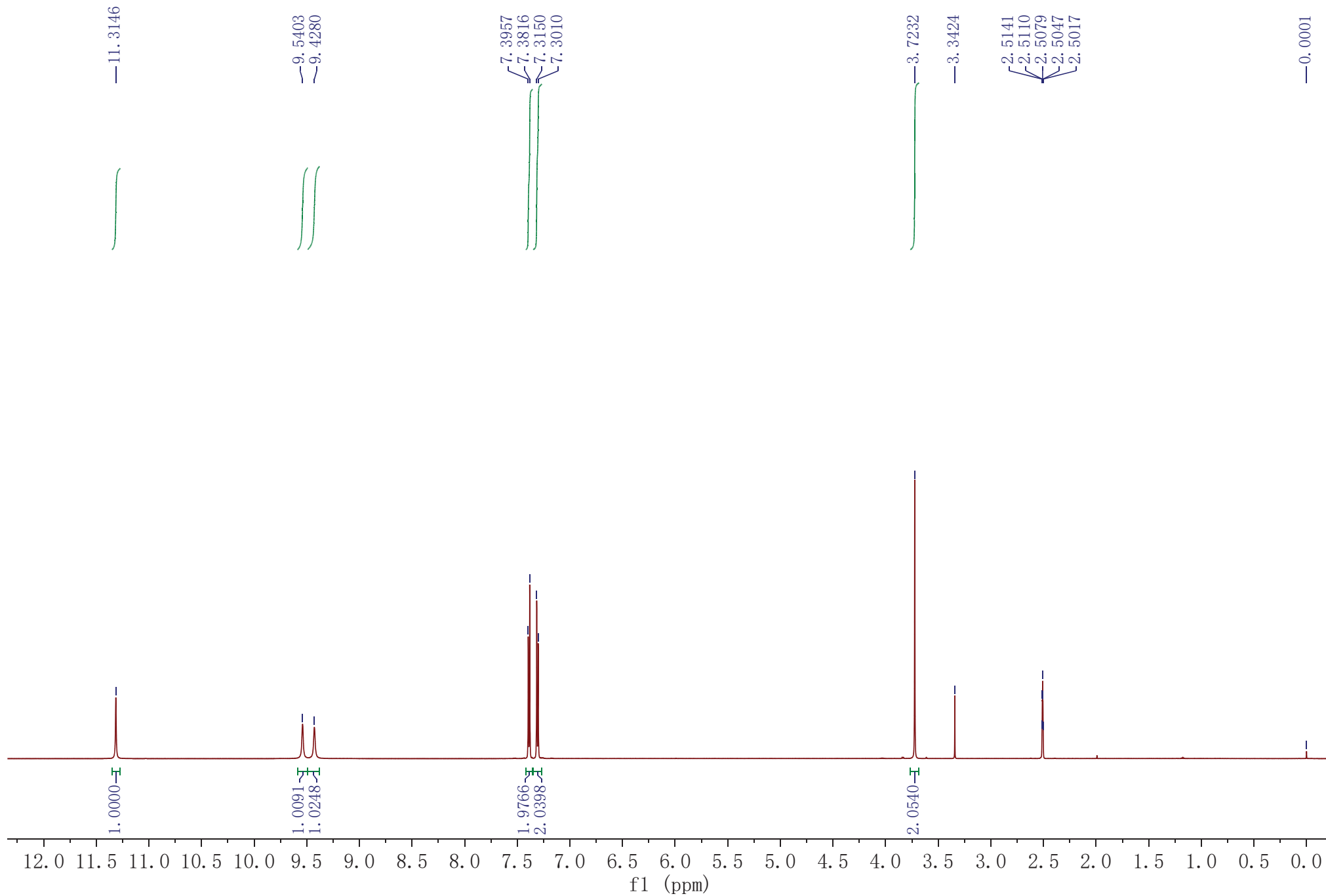

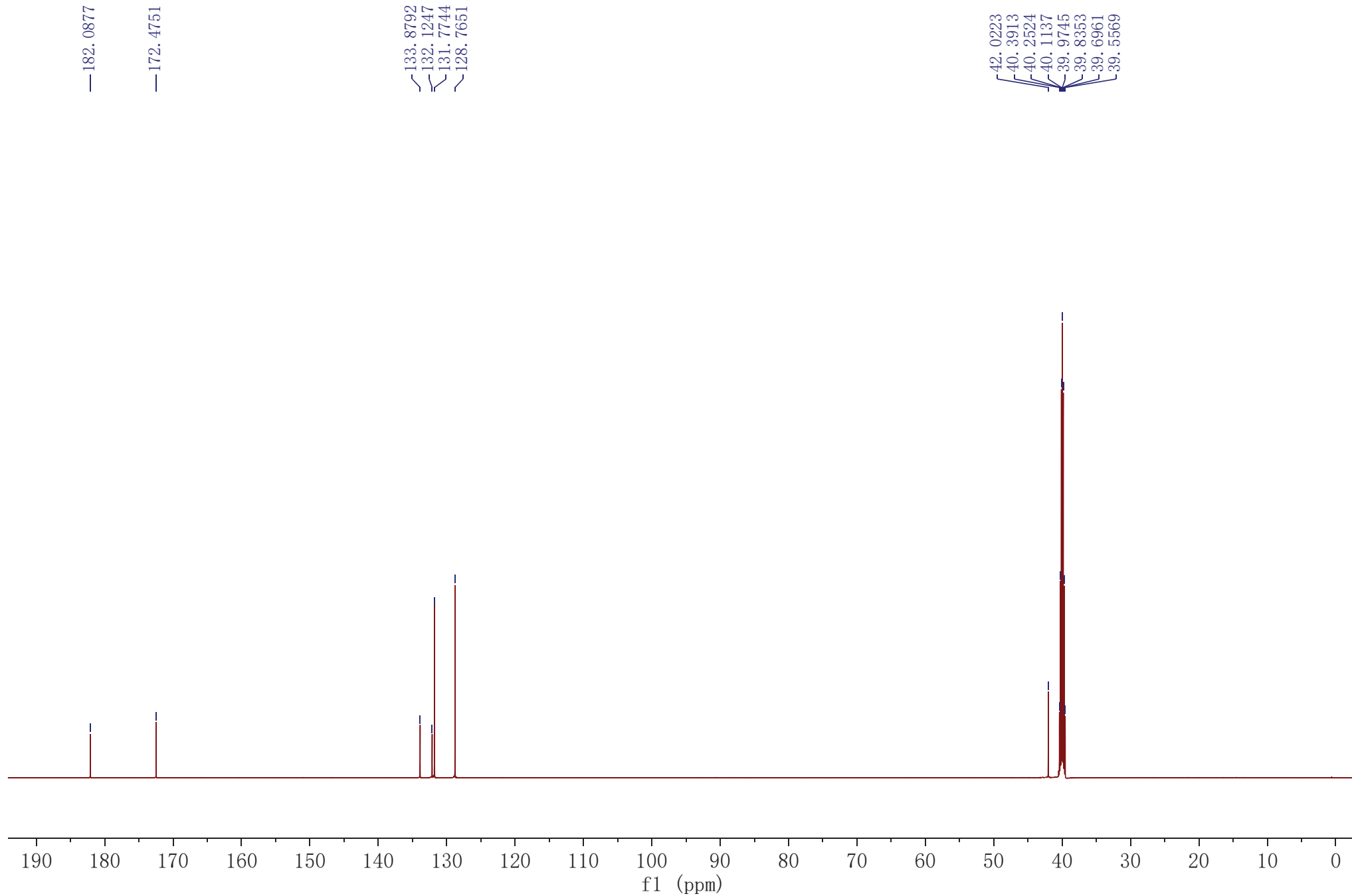

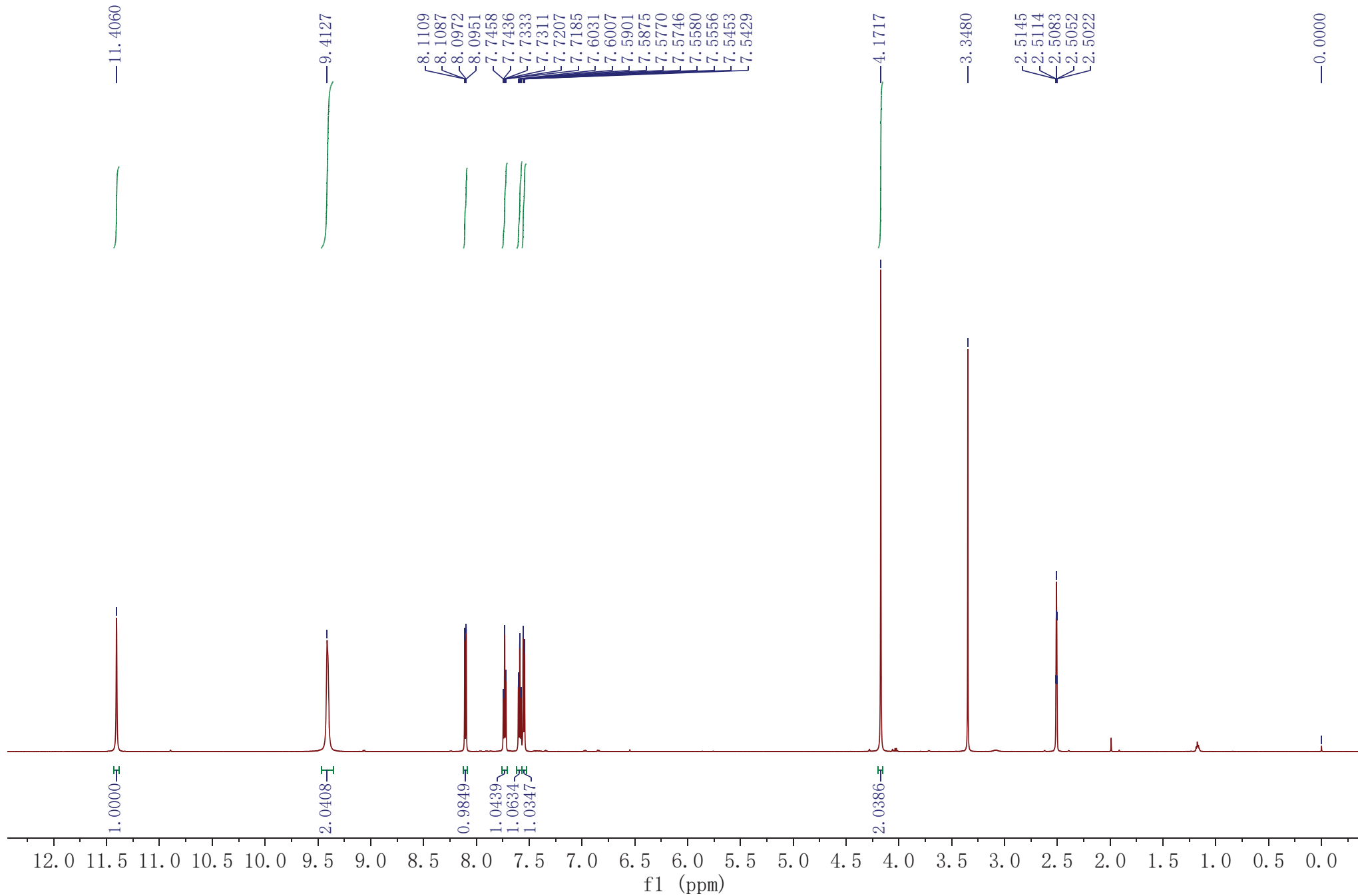

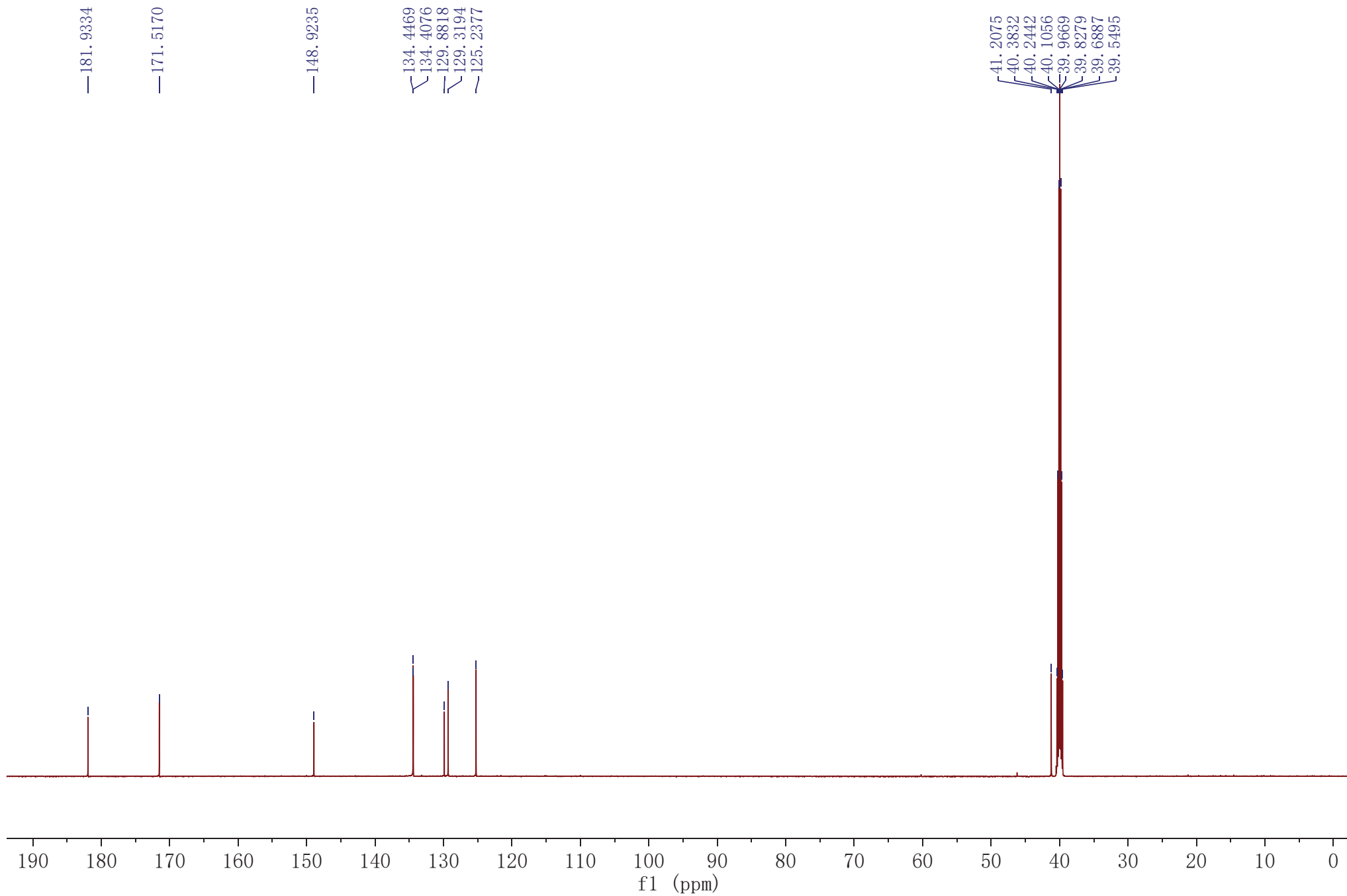

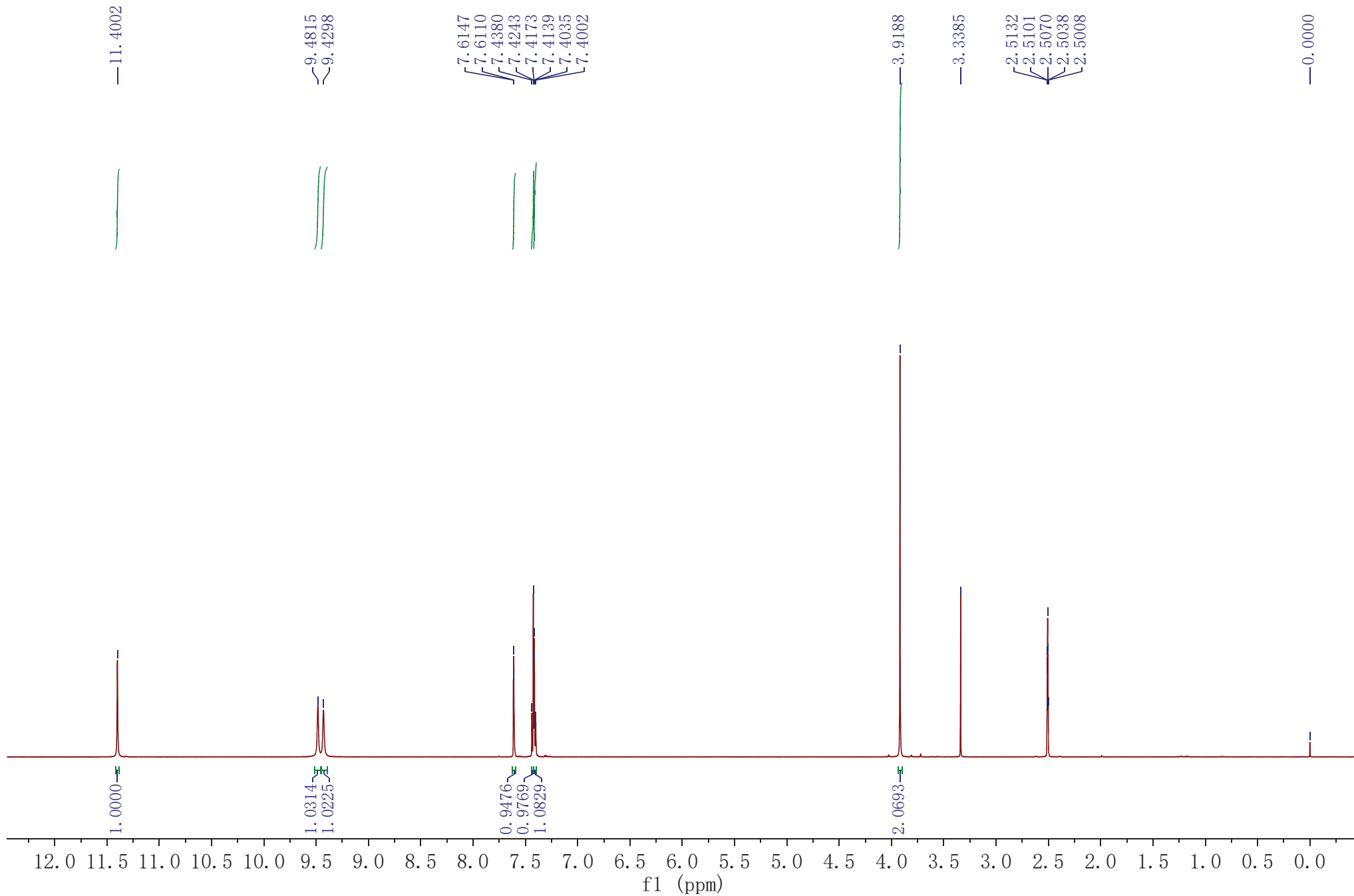

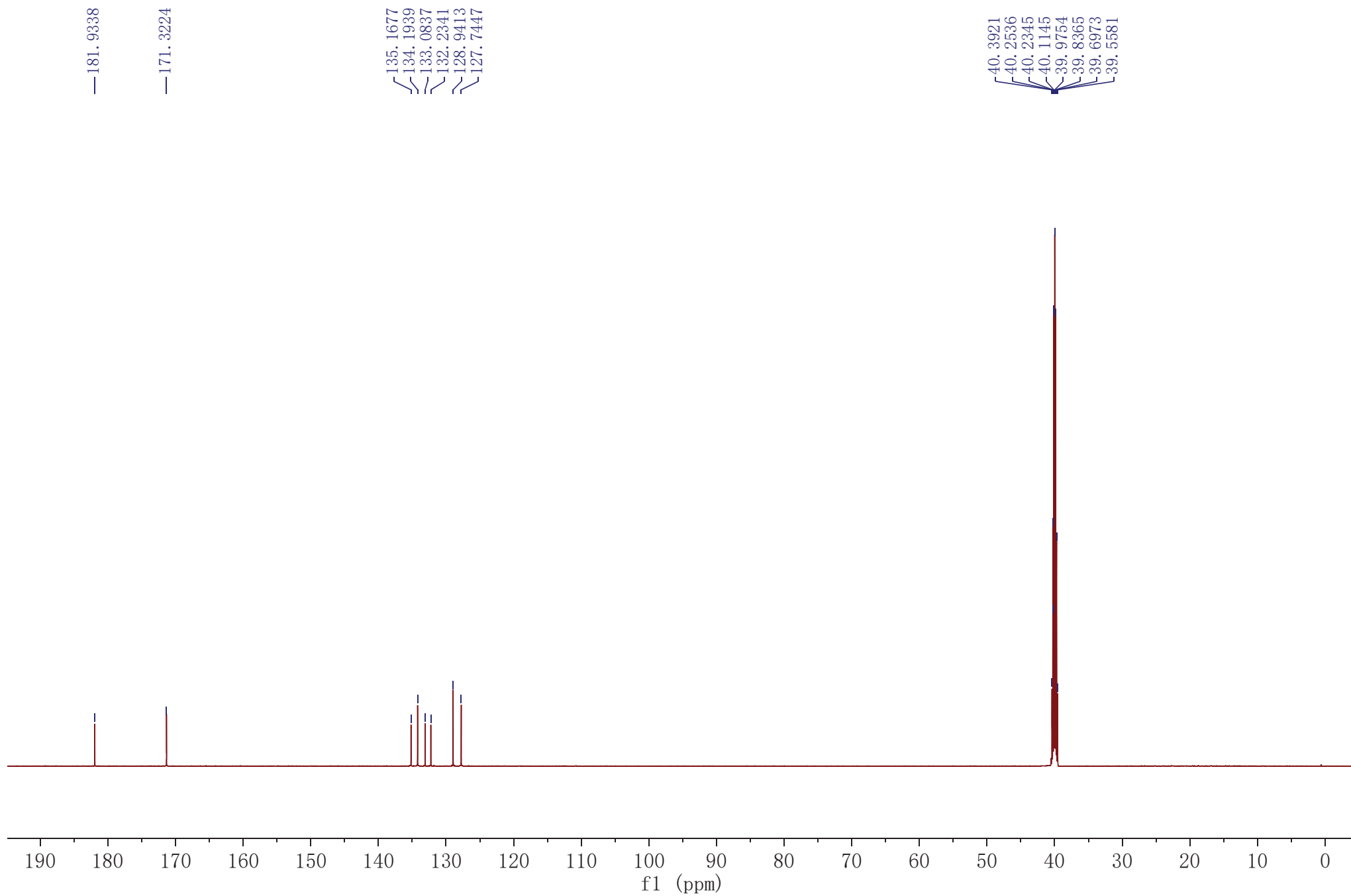

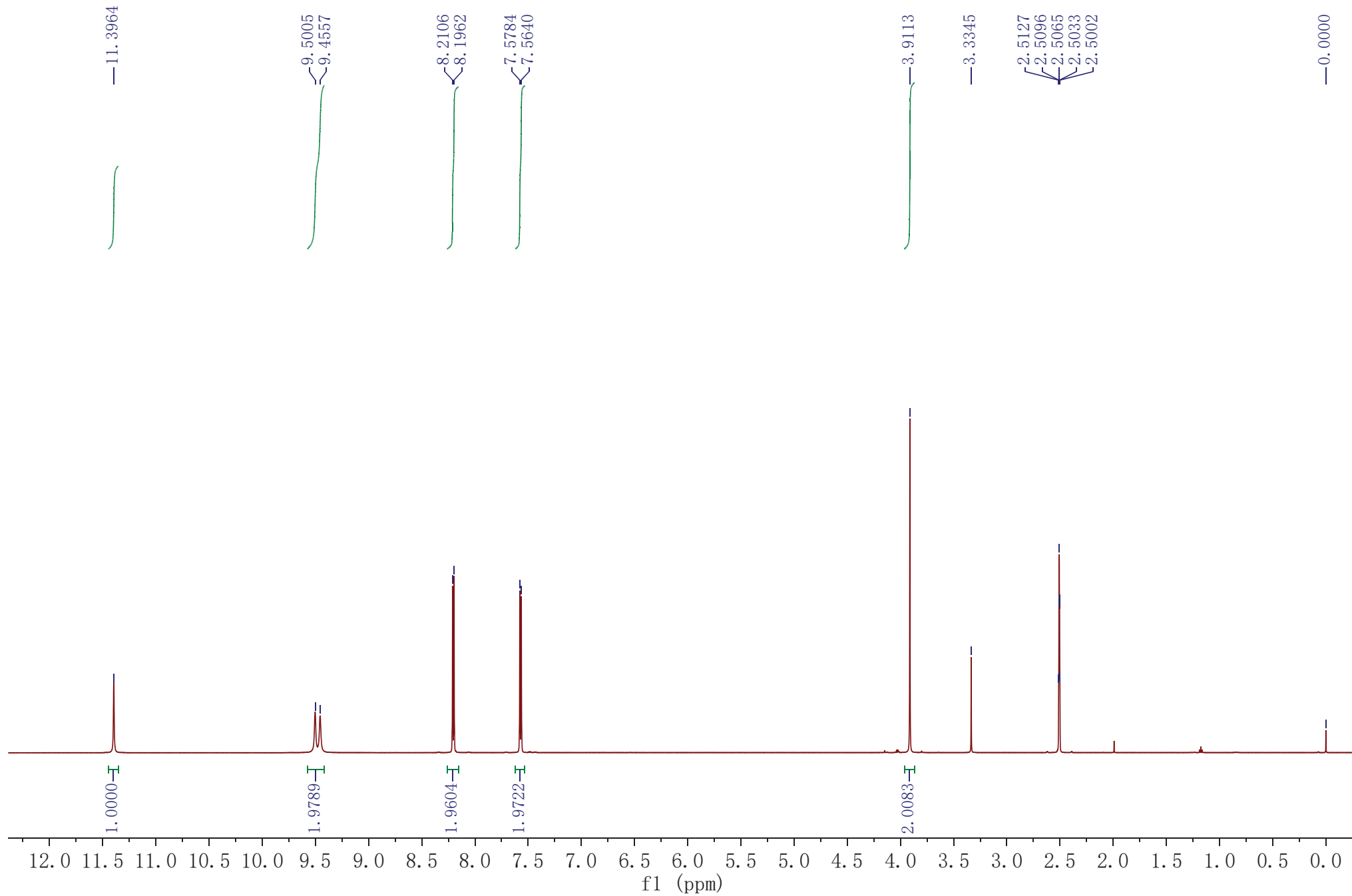

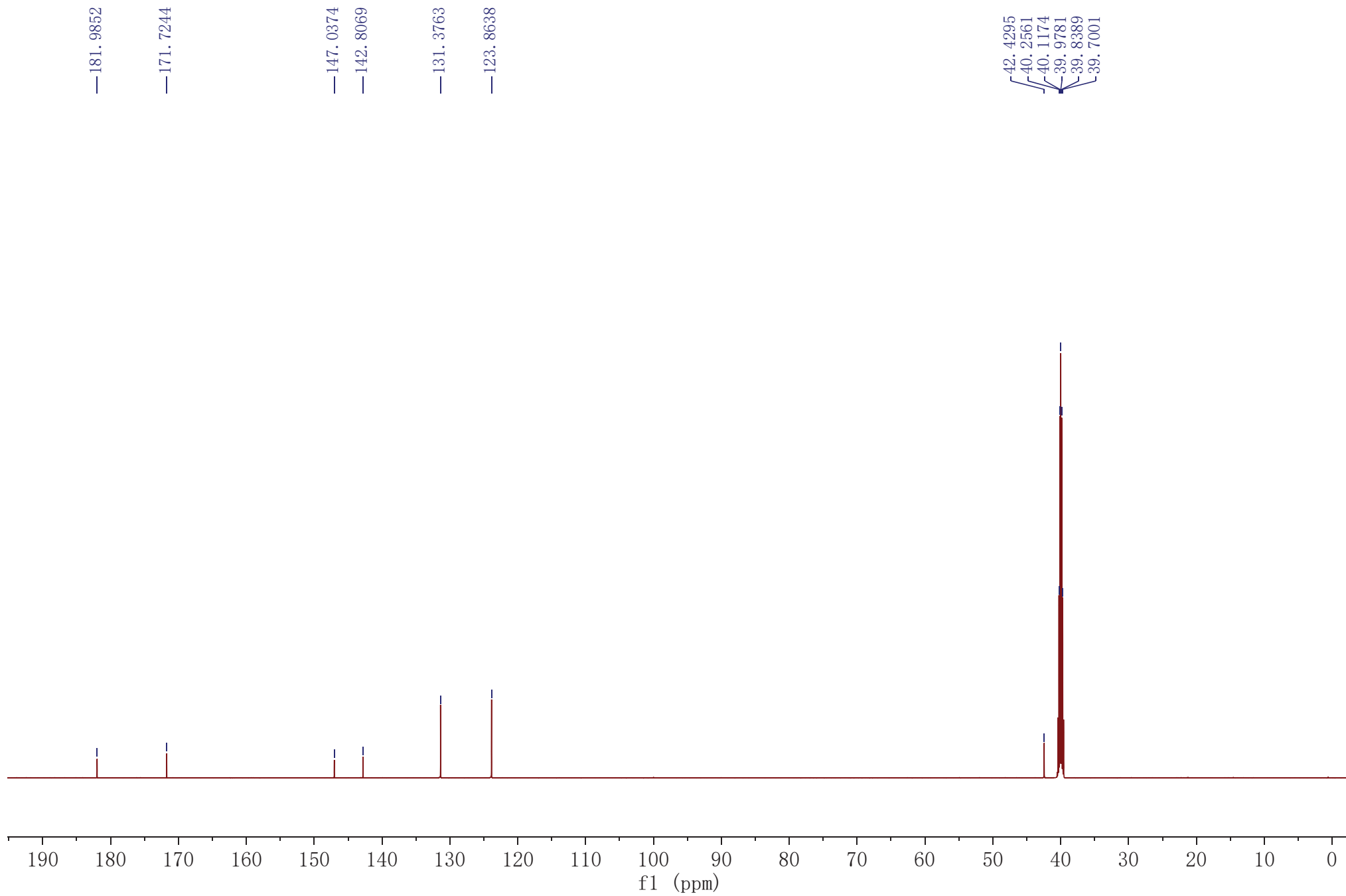

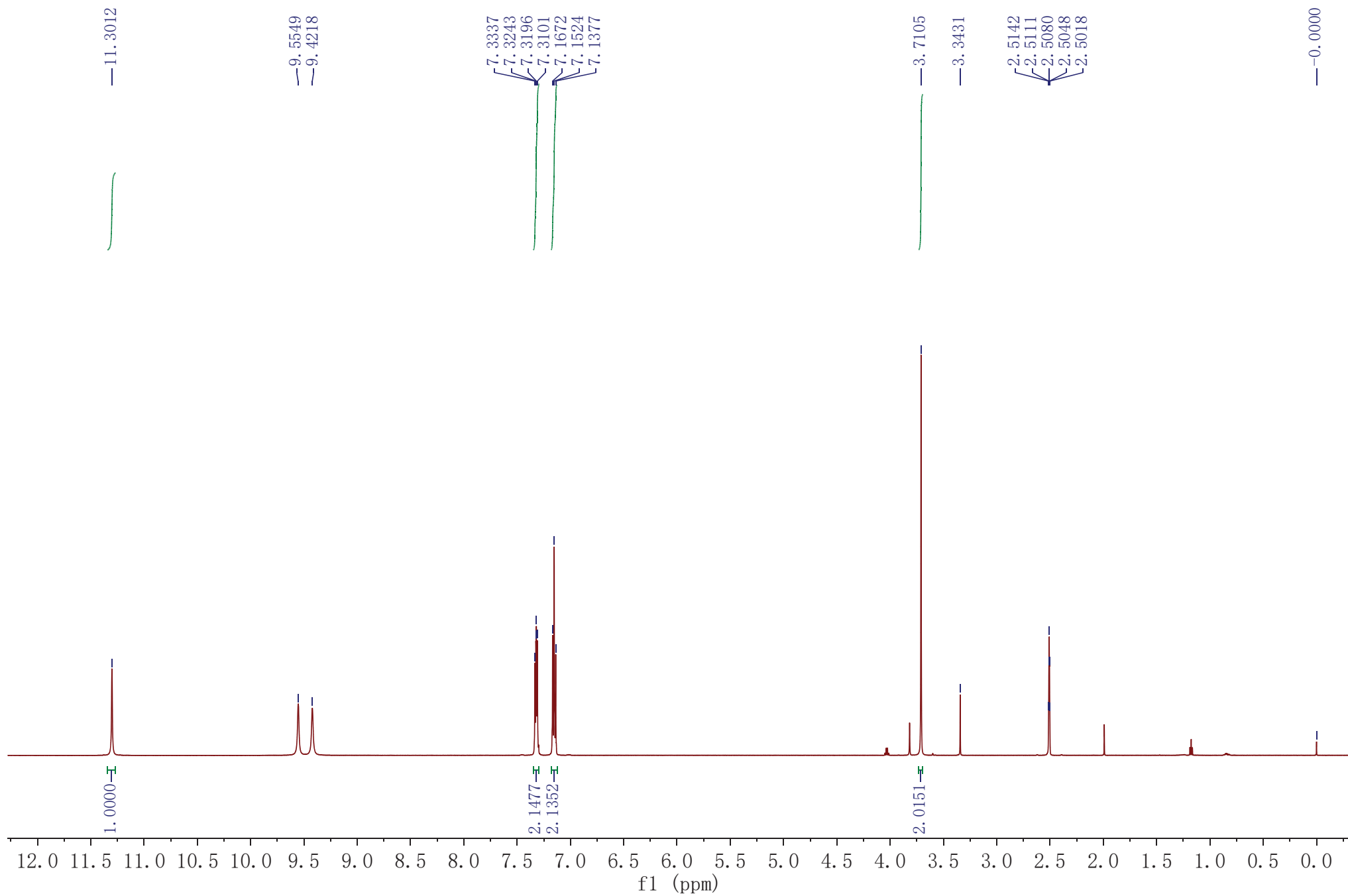

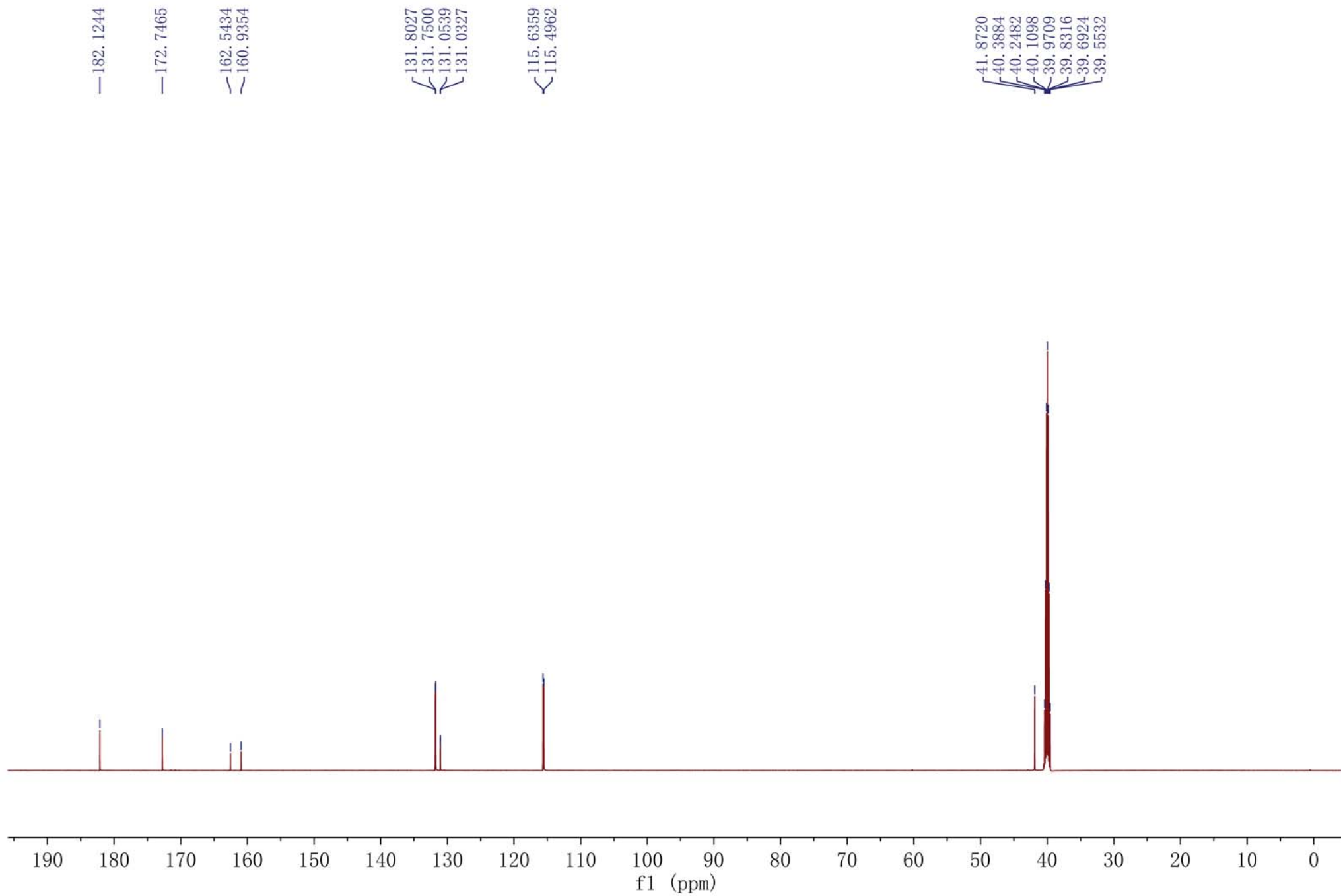

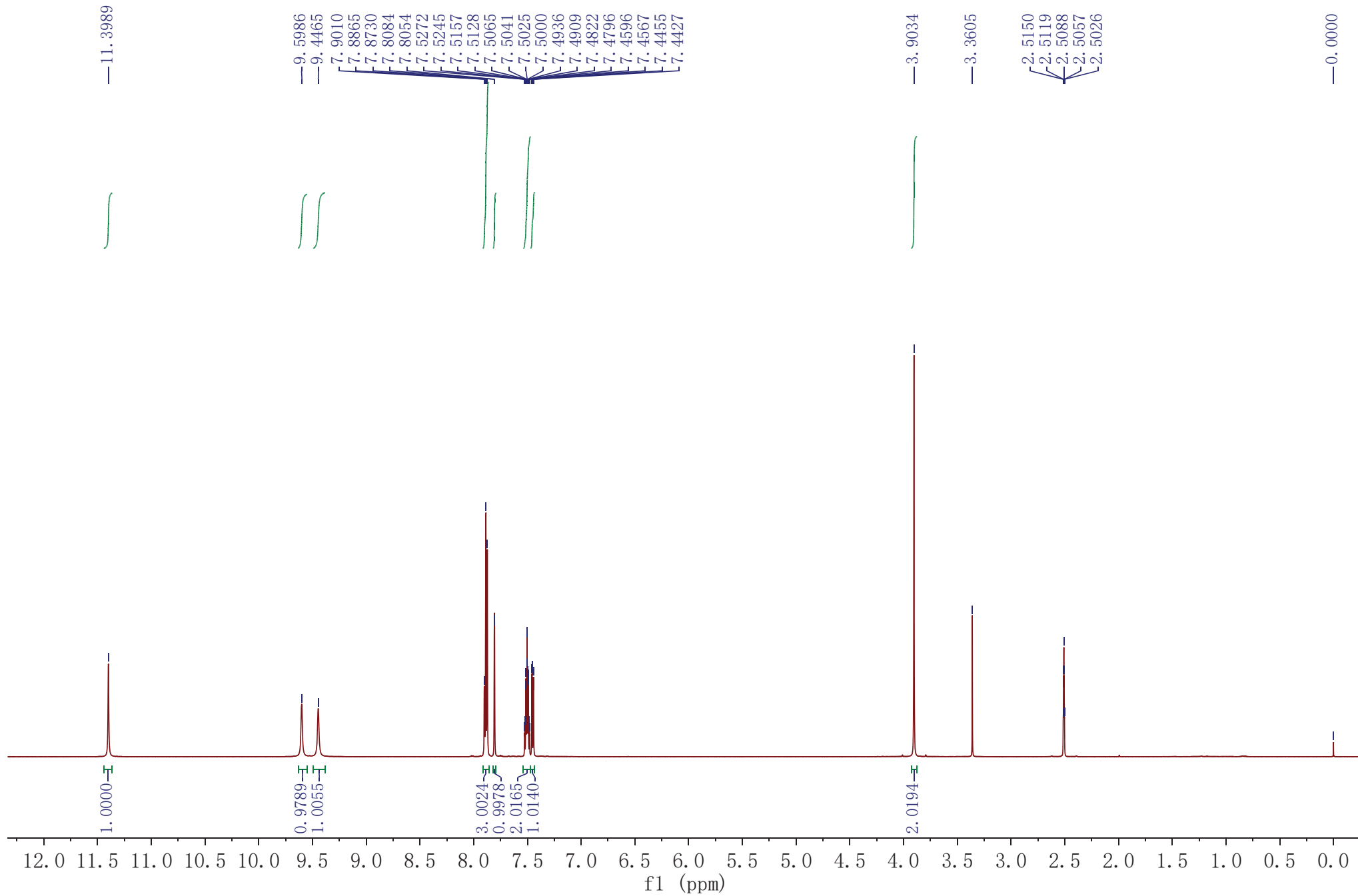

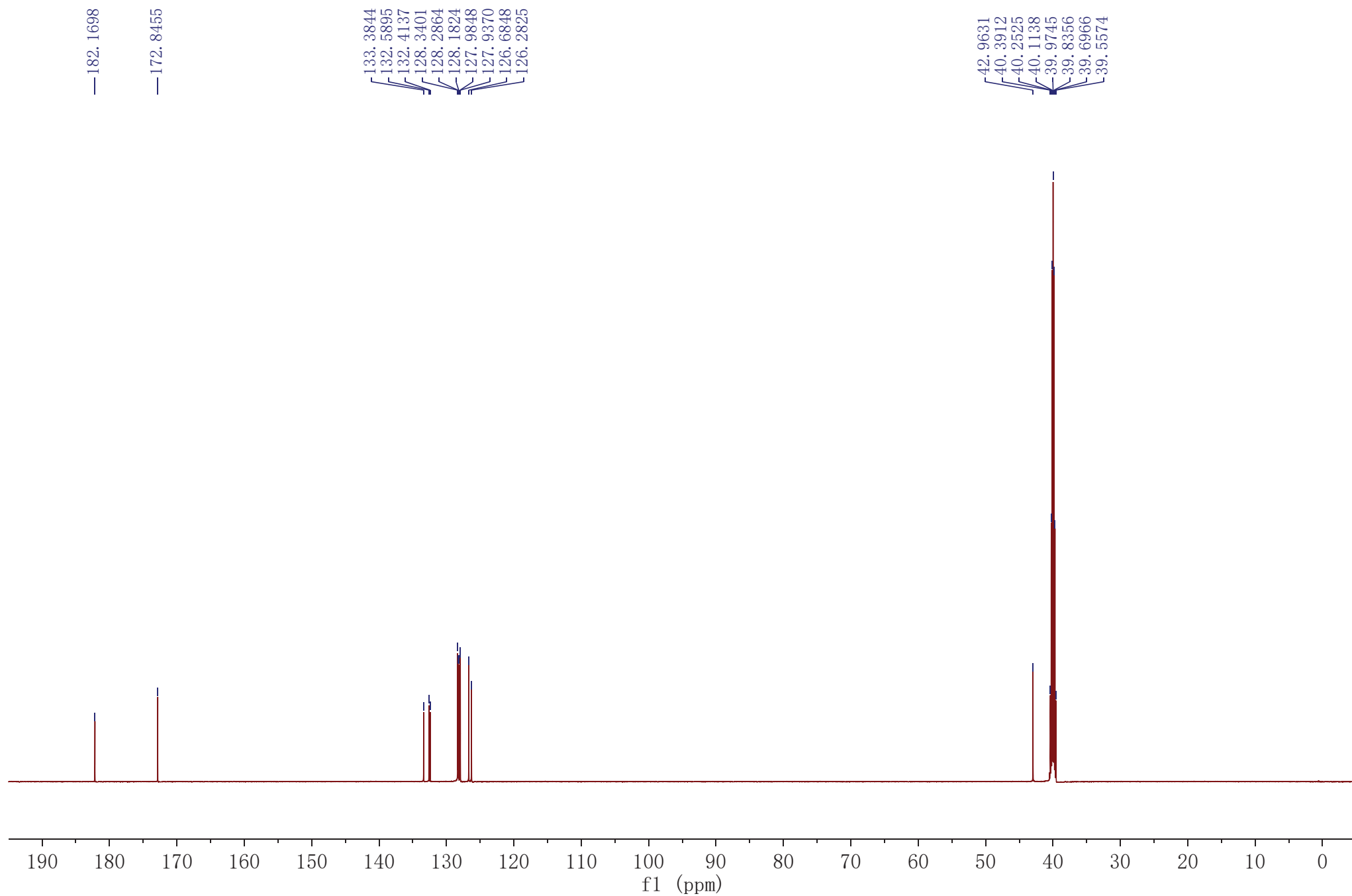

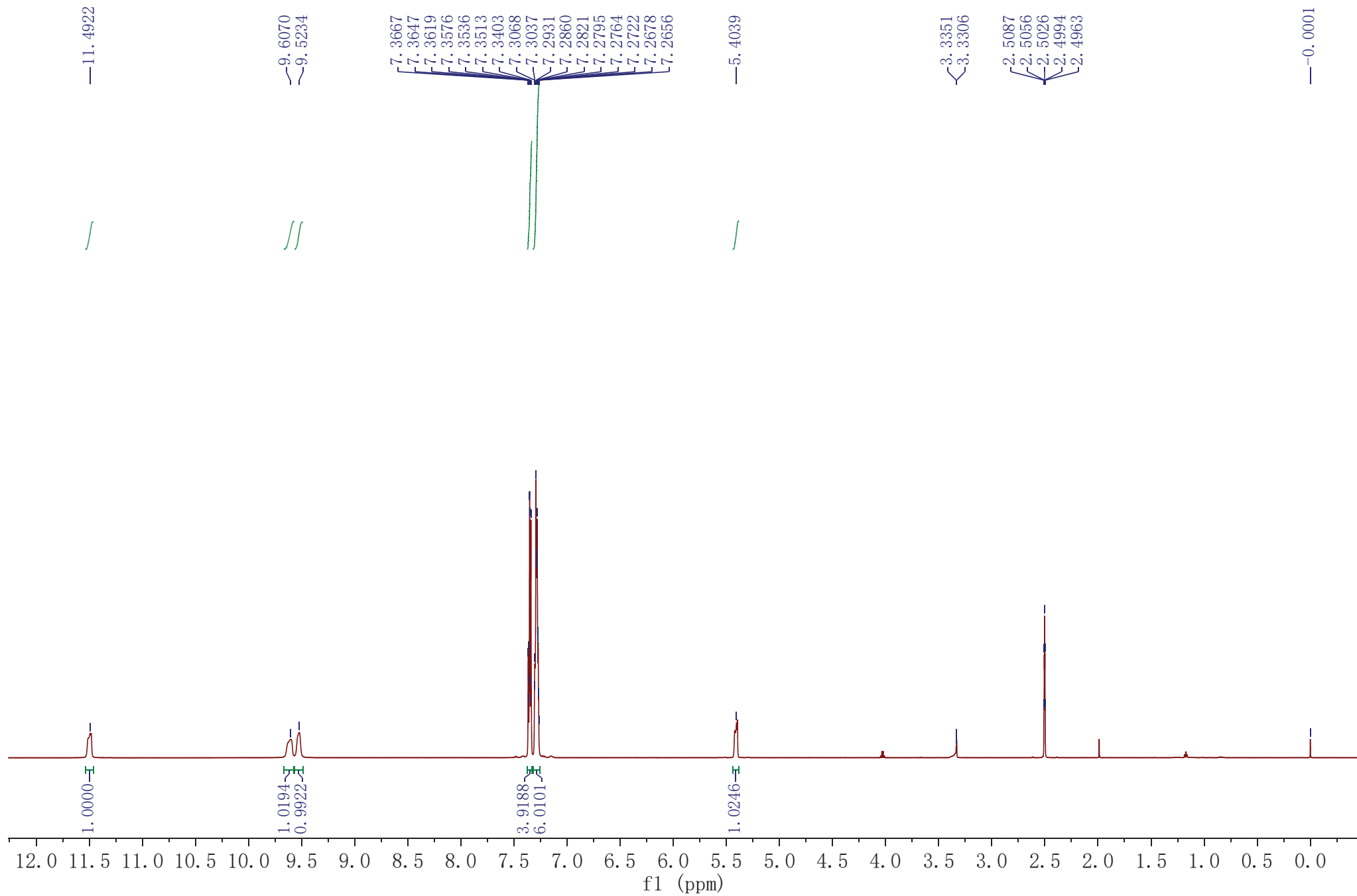

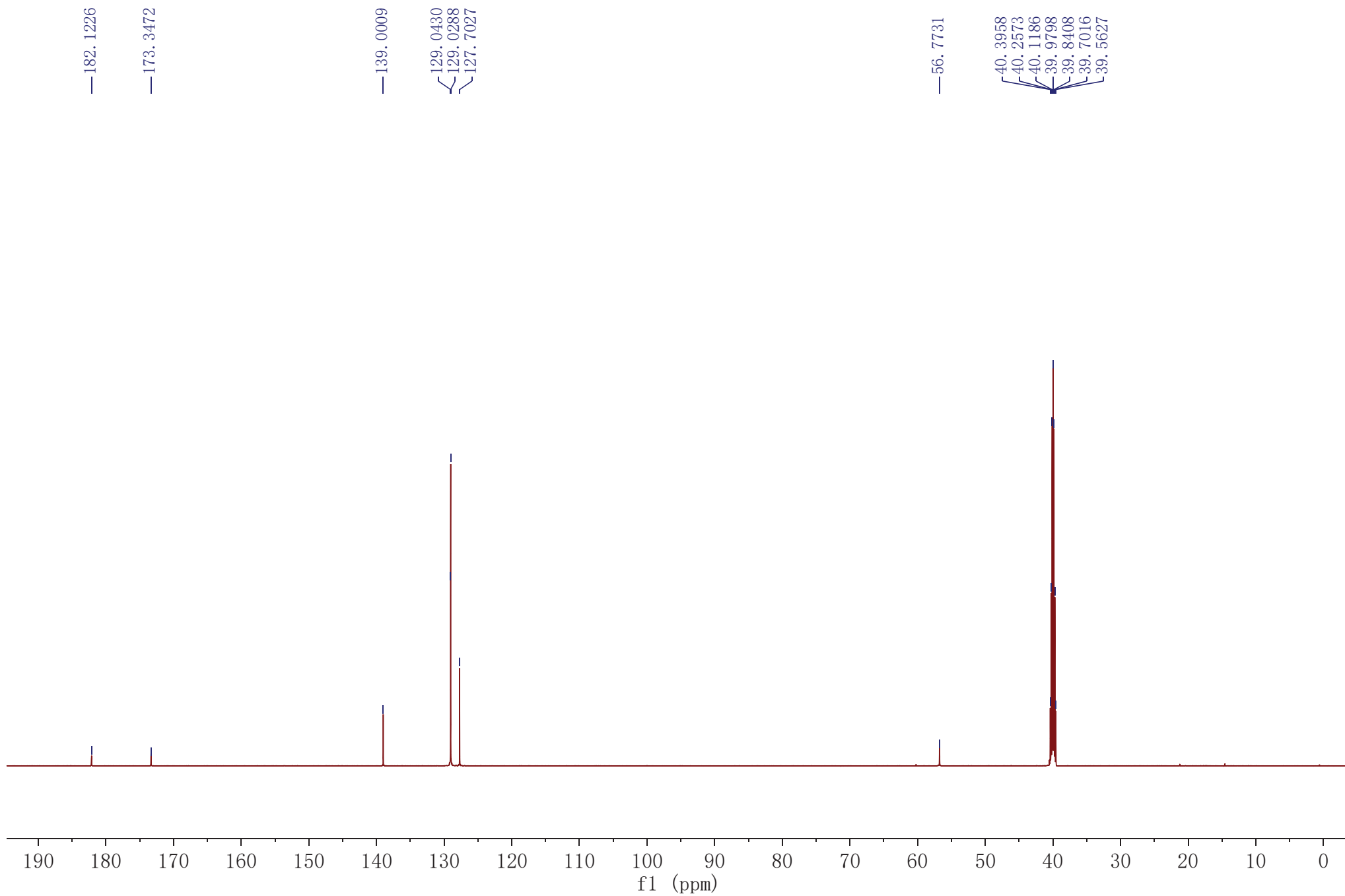

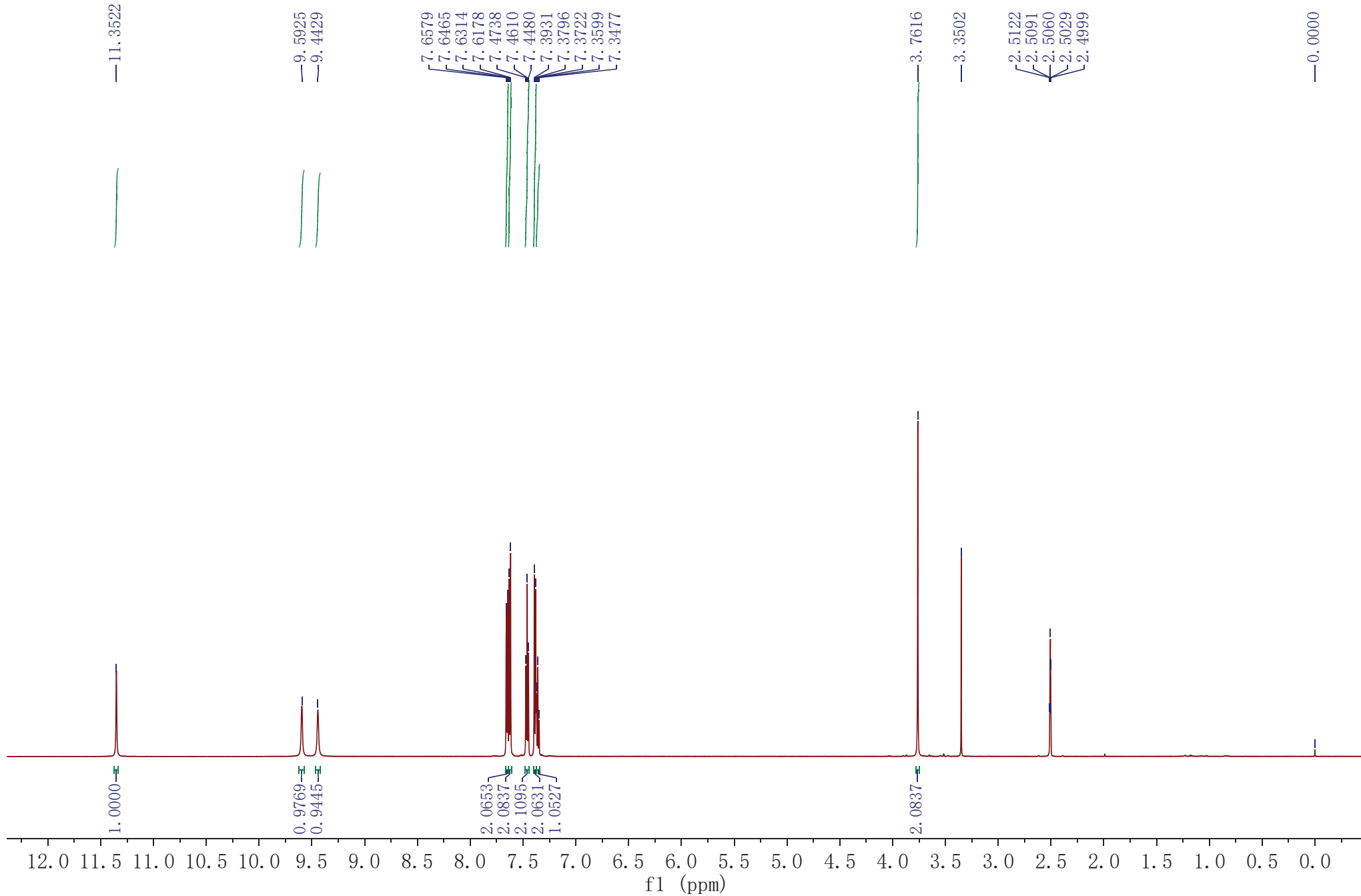

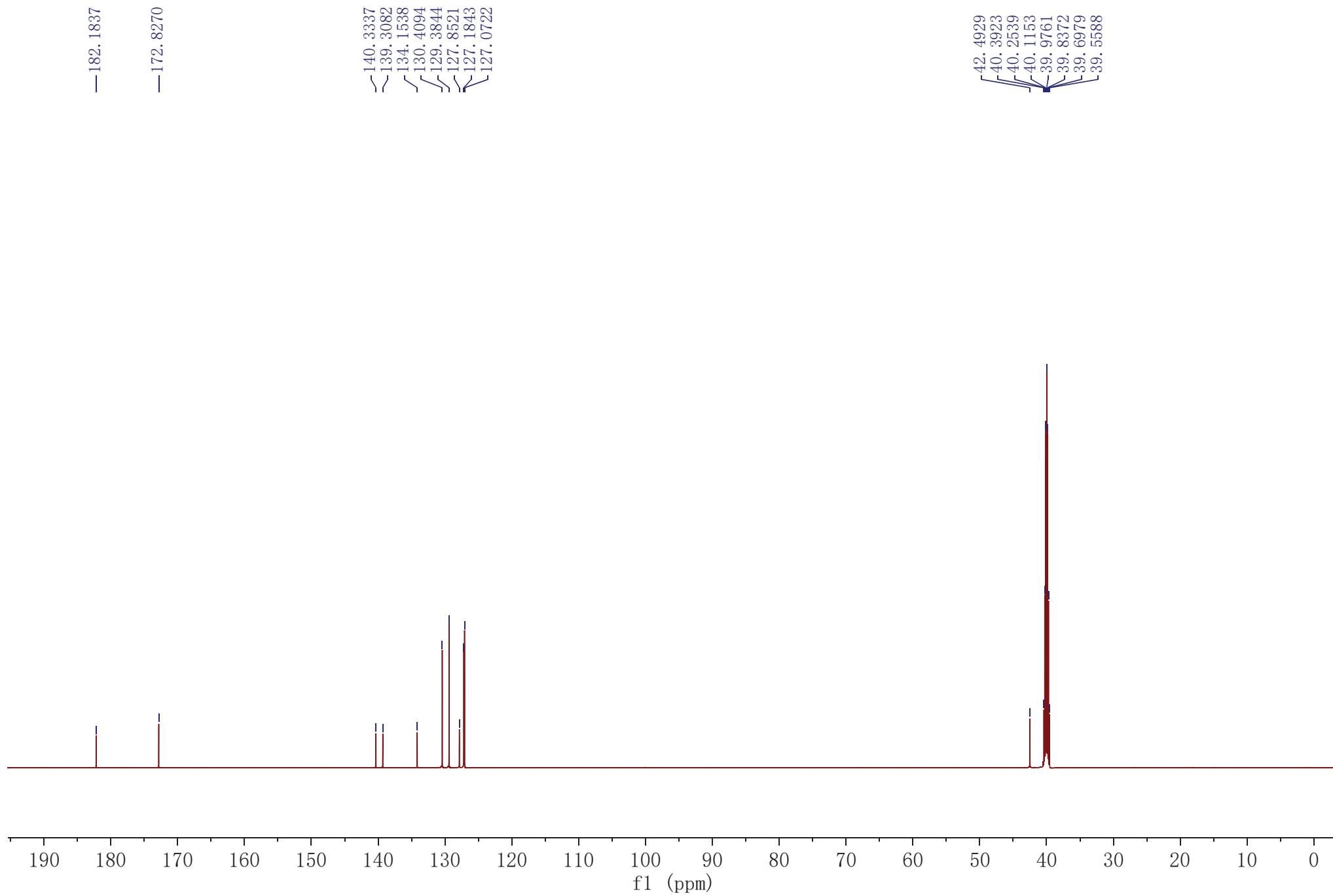

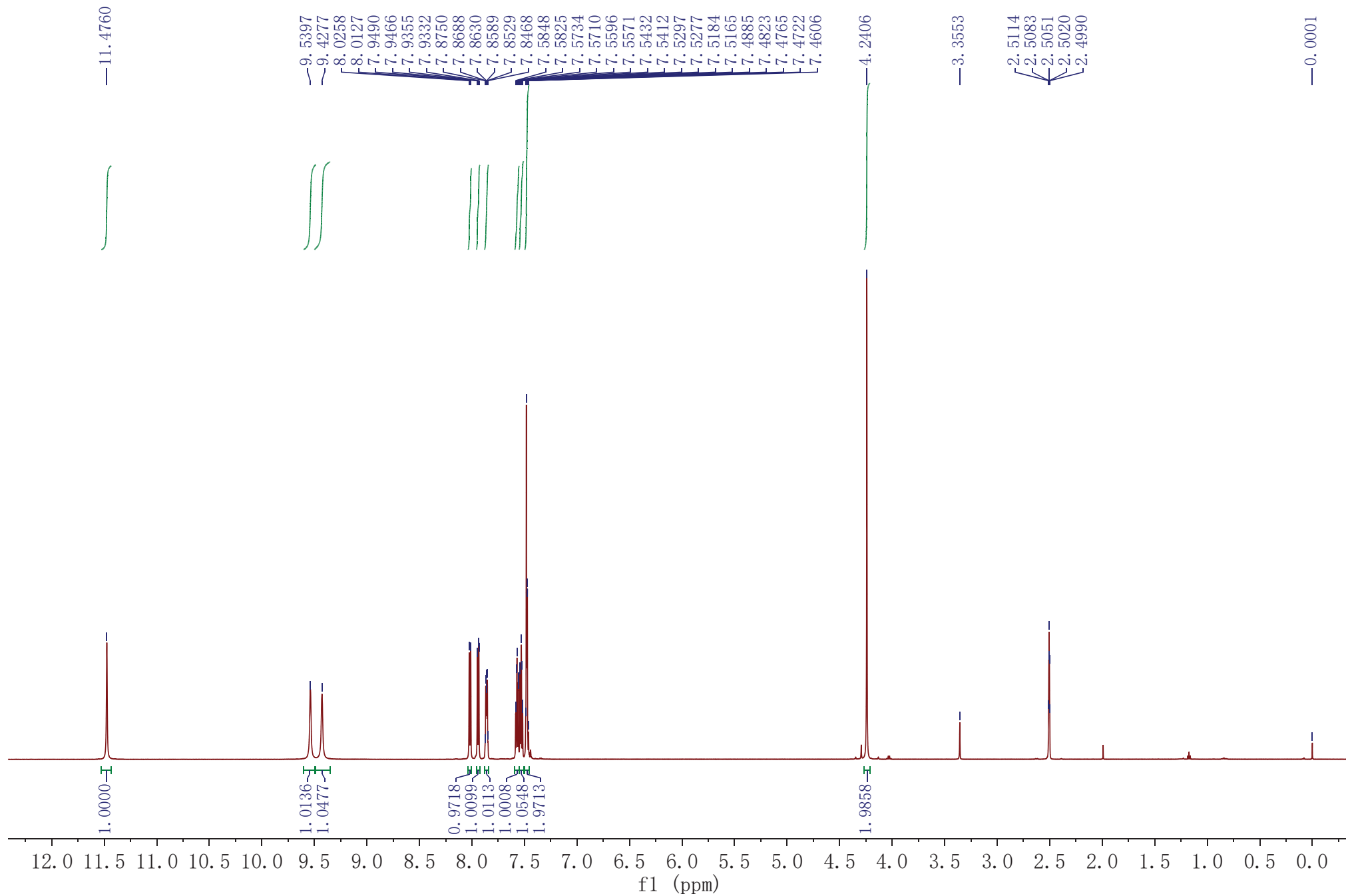

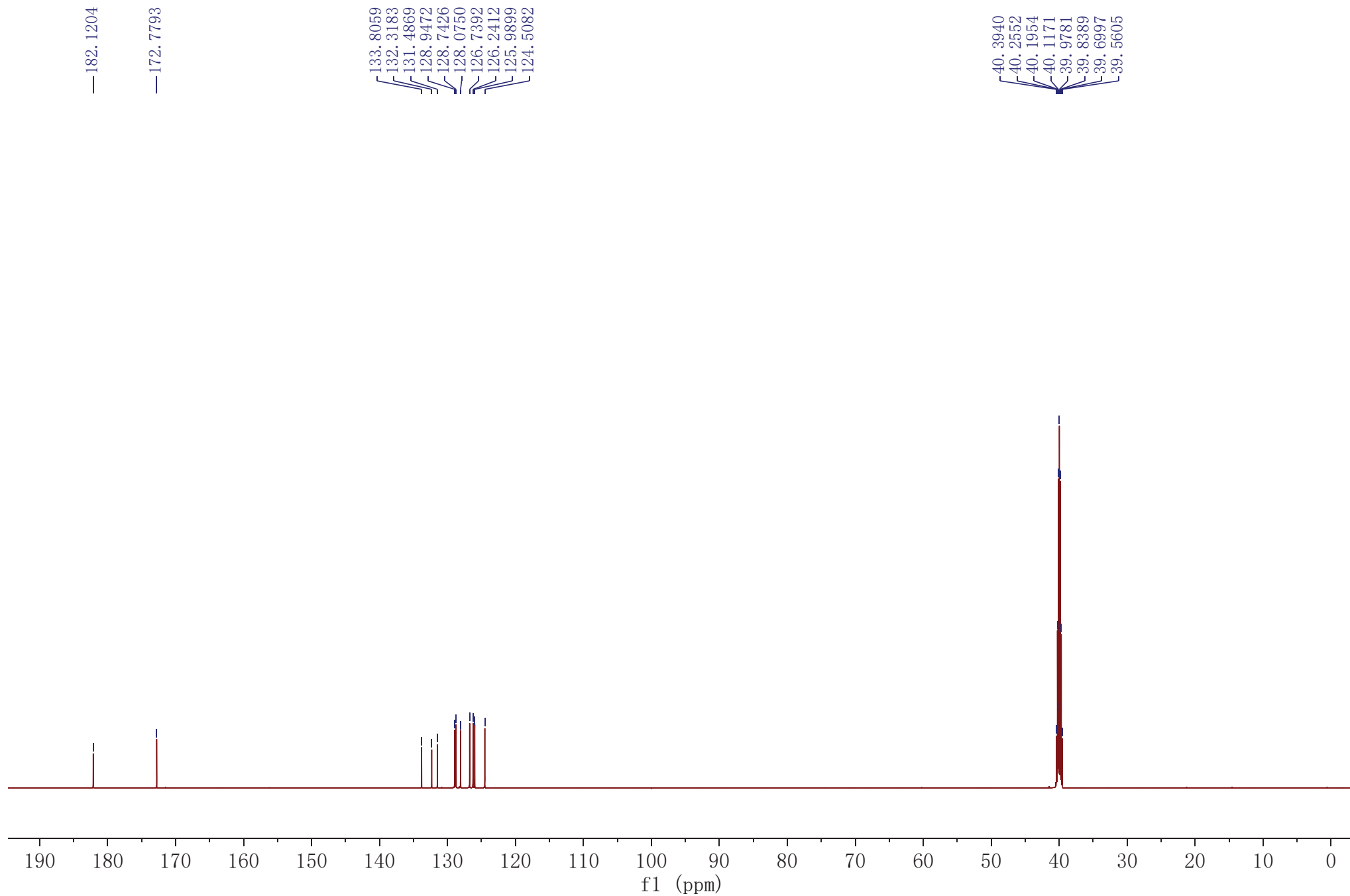

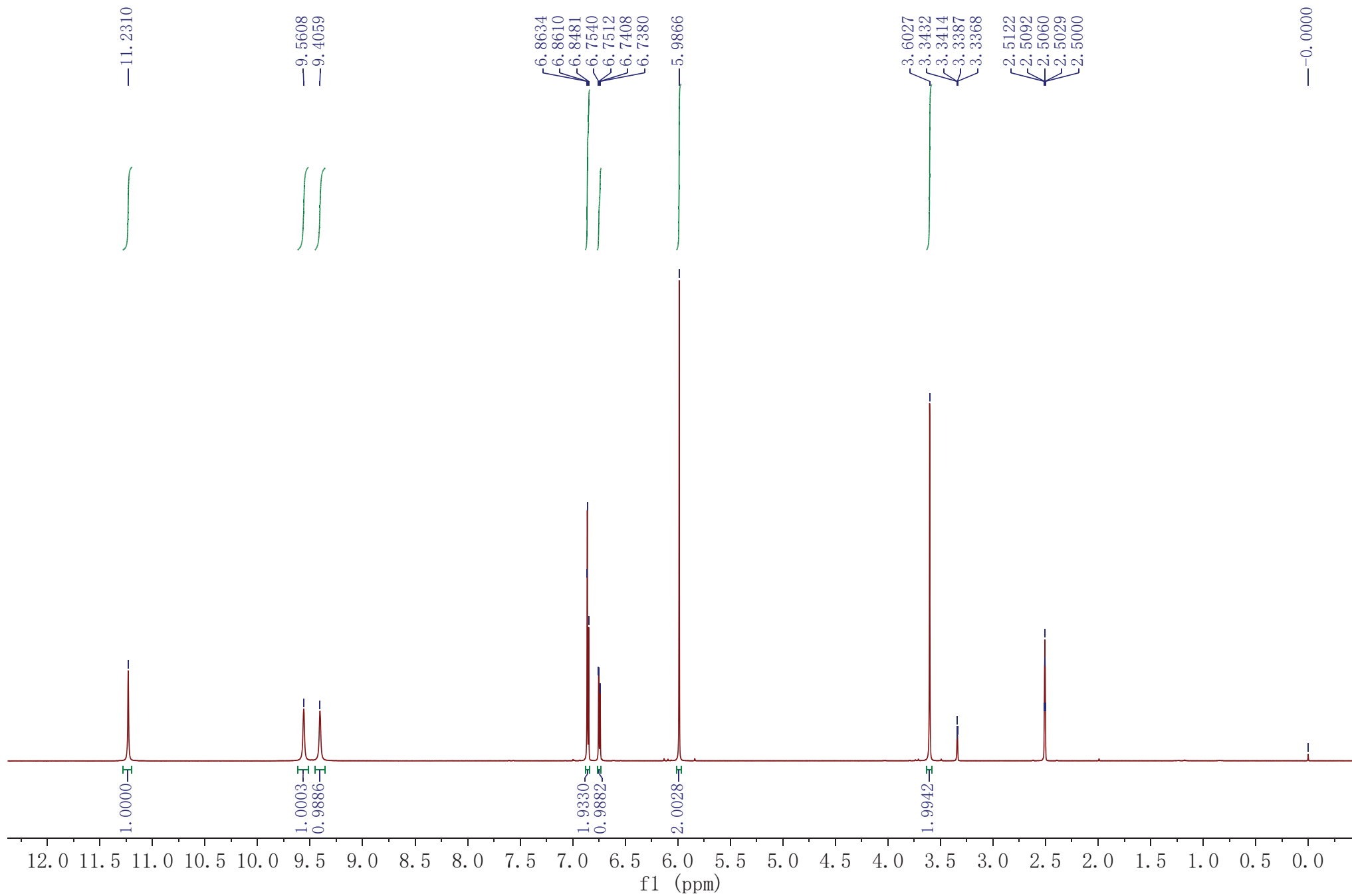

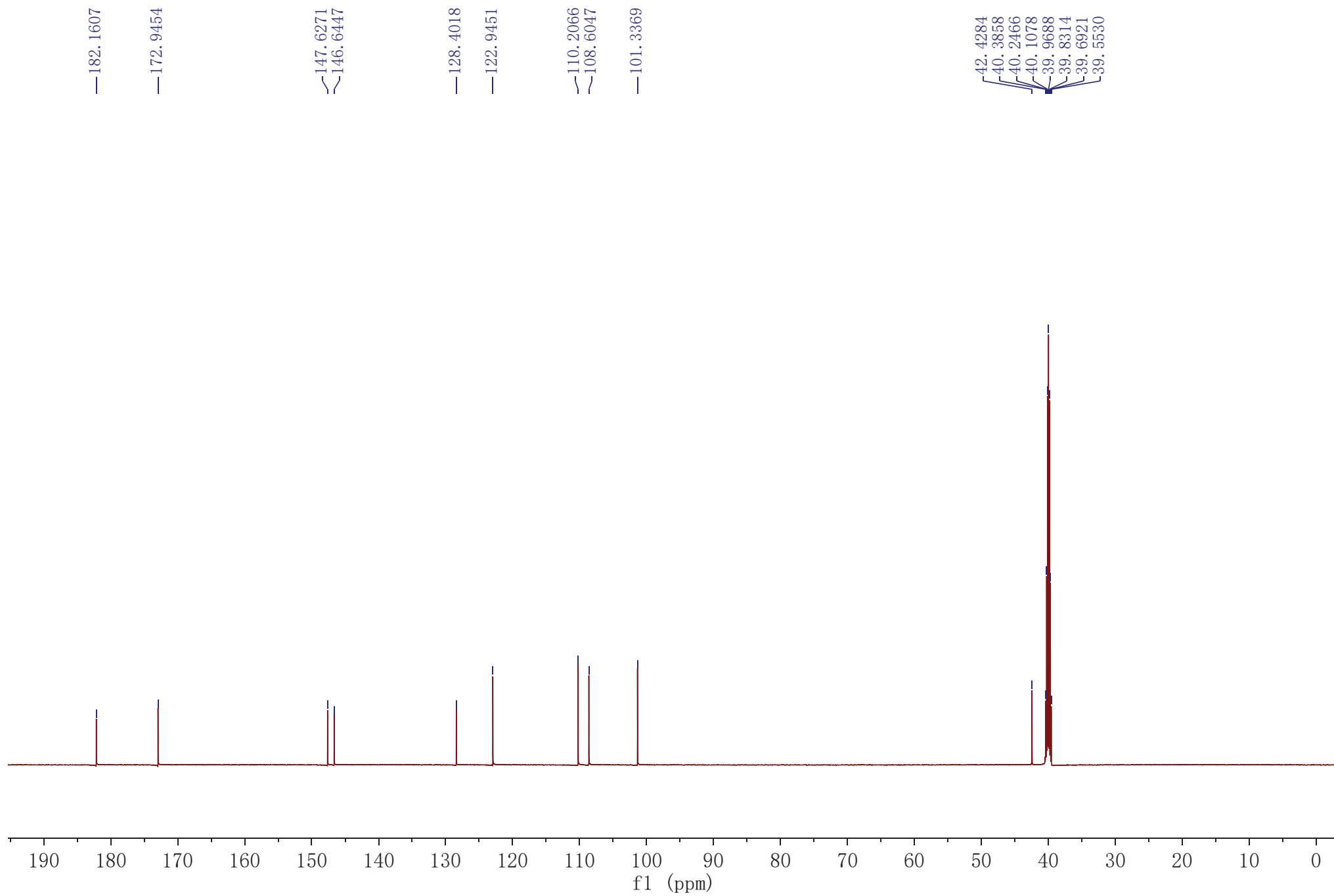

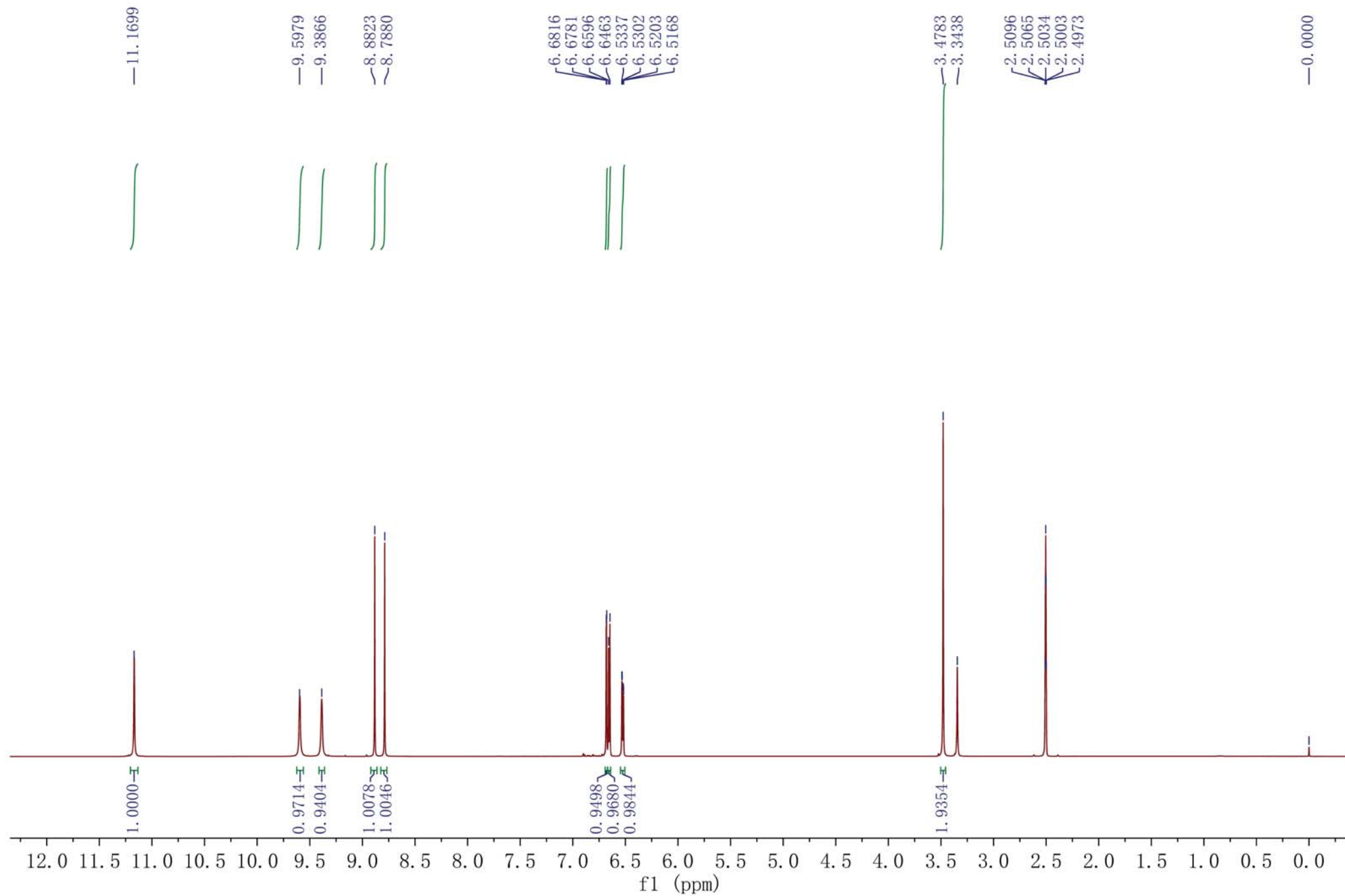

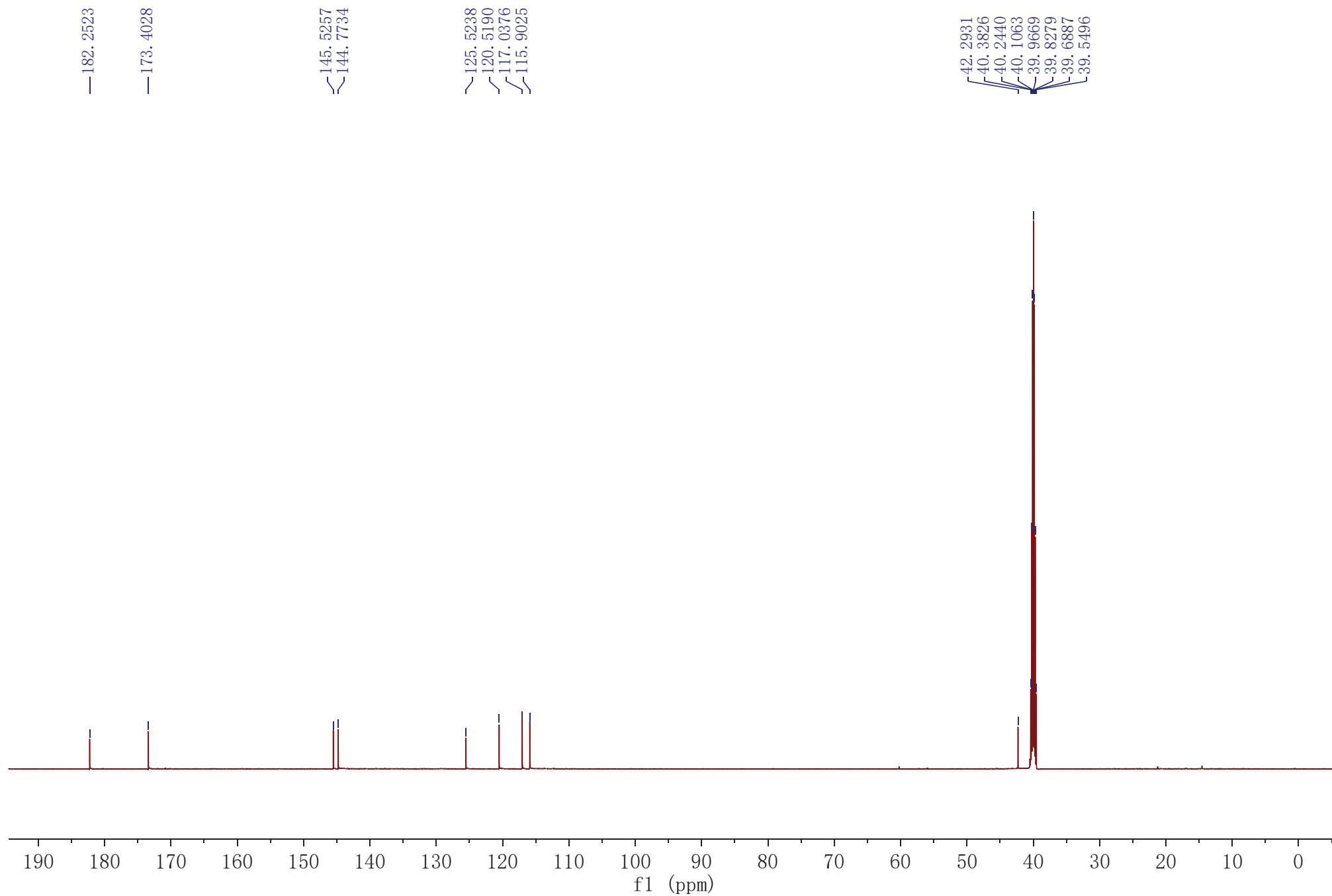

Supplement: Supplemental Material [file IENZ_A_1706503_SM5021.pdf]
